# Projected landscape-scale repercussions of global action for climate and biodiversity protection

**DOI:** 10.1038/s41467-023-38043-1

**Published:** 2023-05-16

**Authors:** Patrick José von Jeetze, Isabelle Weindl, Justin Andrew Johnson, Pasquale Borrelli, Panos Panagos, Edna J. Molina Bacca, Kristine Karstens, Florian Humpenöder, Jan Philipp Dietrich, Sara Minoli, Christoph Müller, Hermann Lotze-Campen, Alexander Popp

**Affiliations:** 1 Potsdam Institute for Climate Impact Research (PIK), Member of the Leibniz Association, PO Box 601203, 14412 Potsdam, Germany; 2 Albrecht Daniel Thaer-Institute of Agricultural and Horticultural Sciences, Humboldt University of Berlin, Berlin, Germany; 3 Department of Applied Economics, University of Minnesota, 1940 Buford Ave, Saint Paul, MN 55105 USA; 4 Department of Environmental Sciences, Environmental Geosciences, University of Basel, Basel, Switzerland; 5 Department of Science, Roma Tre University, Rome, Italy; 6 European Commission, Joint Research Centre (JRC), Ispra (VA), IT-21027 Italy

**Keywords:** Ecosystem services, Biodiversity, Agroecology

## Abstract

Land conservation and increased carbon uptake on land are fundamental to achieving the ambitious targets of the climate and biodiversity conventions. Yet, it remains largely unknown how such ambitions, along with an increasing demand for agricultural products, could drive landscape-scale changes and affect other key regulating nature’s contributions to people (NCP) that sustain land productivity outside conservation priority areas. By using an integrated, globally consistent modelling approach, we show that ambitious carbon-focused land restoration action and the enlargement of protected areas alone may be insufficient to reverse negative trends in landscape heterogeneity, pollination supply, and soil loss. However, we also find that these actions could be combined with dedicated interventions that support critical NCP and biodiversity conservation outside of protected areas. In particular, our models indicate that conserving at least 20% semi-natural habitat within farmed landscapes could primarily be achieved by spatially relocating cropland outside conservation priority areas, without additional carbon losses from land-use change, primary land conversion or reductions in agricultural productivity.

## Abstract (web summary)

Competition between agriculture and land conservation may hinder climate and biodiversity targets. Here, the authors use global models integrating multiple spatial scales to assess how ambitious land conservation action and associated land-use dynamics could drive changes in landscape heterogeneity, pollination supply and soil loss.

## Introduction

Agriculture’s immense impact on the global land system^1,2^ has largely come by two means. The onset of the industrial era, on the one hand, heralded an era of land expansion, with a fivefold increase in agricultural land^3^. On the other hand, intensification and simplification, especially in the last half-century, have fundamentally altered landscapes around the world^4–6^. These transformations have boosted food and material output and enabled agricultural production to keep pace with the growing demand for agricultural commodities. Yet, through habitat destruction and the biotic homogenisation of landscapes that have long been cultured^6,7^, they have also often come at the expense of many regulating nature’s contributions to people (NCP) that have sustained the stable supply of food and material goods^2,8,9^, such as water and climate regulation, pest control, pollination, and soil protection and regeneration.

Land-based climate change mitigation and large-scale biodiversity conservation actions in line with targets formulated in the Paris Agreement and the Kunming-Montreal Global Biodiversity Framework could considerably slow down agricultural land expansion^10,11^. Growing demand for agricultural products and the increased competition for land, however, could further drive intensification in existing agricultural landscapes and widely compound the issue of landscape simplification^12,13^. This, in turn, could cement observed trade-offs between the appropriation of material and many key regulating NCP^2,9,14^. Regulating NCP are not only critical for the sustained productivity of agricultural landscapes^8,15,16^, but also increase their resilience^17^, e.g., in the face of extreme events such as droughts or floods, or against pest and disease outbreaks^18,19^.

Recent work has shown that landscape complexity, which includes compositional and configurational heterogeneity, consistently increases both biodiversity and the supply of key NCP^20^. In particular, the amount of (semi-)natural habitat in agricultural landscapes has shown to be a good predictor of crop pollination^21–23^, as well as pest enemy diversity^24^ and pest control^15,25,26^, while its decline has shown to reduce pollination, pest control and, subsequently, crop yields^8^. In particular, 75% of all crop species cultivated globally depend on biotic pollination. Wild pollinator decline could hence put $235 to $577 billion of crop output at risk^2^, which could mostly affect low-income countries^27,28^. Higher structural diversity between fields has also shown to protect soils and counteract soil loss by wind and water erosion in agricultural settings^4,29–31^. Landscape approaches are therefore an important means to mitigating soil degradation, especially if they are combined with other on-site measures, such as agroforestry practices, cover crops, reduced tillage or plant residues^32–34^.

Historically, integrated land-system models have emphasised global or regional-scale dynamics and have neglected finer-scale drivers of ecosystem change, such as landscape simplification, that have caused sharp declines in biodiversity^35^ and the supply of many important NCP^2,8,36,37^ in agricultural landscapes. The computational challenges to linking coarse model dynamics and outputs to changes at the landscape level have indeed been plenty and applicable fine-scale data was difficult to obtain and process^38,39^. However, this only coarse representation of ecosystem change and the limited spatial representation of impacts in global-scale analyses may cause biased assessments and, in consequence, imbalanced policy frameworks^40^.

**Fig. 1:**
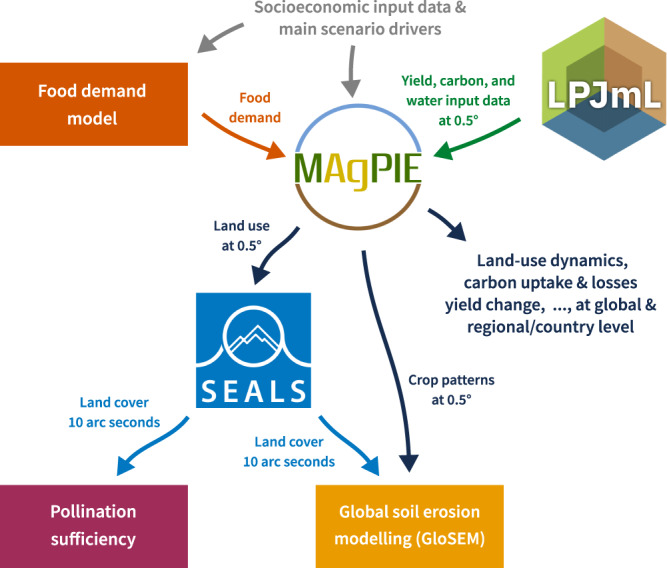
Overview of the MAgPIE-SEALS modelling framework. SEALS is coupled to the MAgPIE model during post processing of scenario model runs. SEALS receives spatially explicit land cover information from MAgPIE to spatially allocate projected land cover changes at 0.5 degrees to a resolution of 10 arc seconds (300 m × 300 m at the equator). NCP-related indicators pollination sufficiency and soil loss are then derived from the high resolution land cover maps. In GloSEM, C-factor values for cropland are estimated based on spatially explicit crop patterns at 0.5 degrees (see Methods).

Here, we apply an integrated and globally-consistent modelling framework to assess how growing land competition and associated global land-use dynamics could drive changes in various indicators of material and key regulating NCP across multiple scales (see Supplementary Table 1). To this end, we have coupled the open-source land-system model MAgPIE^10,41–43^ (Model of Agricultural Production and its Impact on the Environment v4.3.5) with the Spatial Economic Allocation Landscape Simulator^44,45^ (SEALS, see Fig. 1). MAgPIE uses a cost-optimisation approach to simulate global land-system dynamics within the 21st century. It thereby combines a wide range of socio-economic and spatially explicit biophysical information from the dynamic global vegetation, crop and hydrology model LPJmL^46–49^ (see Methods), including yield patterns, water availability and carbon stocks at a spatial resolution 0.5 degrees (55 km × 55 km at the equator). The SEALS model, on the other hand, downscales simulated land-cover changes from MAgPIE to a spatial resolution of 10 arc seconds (300 m ×300 m at the equator), based on adjacency relationships, physical suitability and conversion eligibility.

In this study, we used the coupled MAgPIE-SEALS modelling framework to derive fine-scale changes in pollination sufficiency (NCP 2) and soil loss by water erosion (NCP 8) across four exploratory land-use scenarios. Pollination sufficiency is defined by the amount of semi-natural habitat within foraging distances typically found in wild pollinator communities around cropland^36^. We use this indicator both as a proxy for the supply of wild pollination in cropland areas^22,50^ and for configurational landscape heterogeneity. Configurational landscape heterogeneity is associated with variations in the supply of a range of other regulating NCP, such as biological pest control^2,8^ (NCP 10) and an important driver of biodiversity^20^. Soil loss by water erosion, on the other hand, is a major driver of soil degradation at the global scale, undermining the supply of a wide range of soil-related material and regulating NCP^32,51^. In addition, we complemented our analysis with a range of established global or regional indicators for material production (NCP 11-13) and climate regulation (NCP 4), which were directly derived from the MAgPIE model. The categorisation of the NCP covered in this study is based on the IPBES conceptual framework^2^ (Supplementary Table 1).

In our scenarios, we contrast a ‘business-as-usual‘ (BAU) scenario with three different exploratory land-system interventions that are consistent with the targets formulated in the Paris Agreement and the Kunming-Montreal Global Biodiversity Framework of the Convention on Biological Diversity^52^ (see Methods). The BAU scenario serves as our reference scenario and follows the ‘middle-of-the-road’ shared socioeconomic pathway^53^ (SSP2) for the land-use sector. It features moderate population and income growth, as well as currently implemented national land conservation policies. The alternative scenarios include a scenario of area-based conservation attention that covers about 30% of the land surface in conservation priority areas^54^ by 2030 (PROTECT), to which we successively add ambitious action for carbon-focused land restoration consistent with the Paris Agreement^10,55,56^ (COACTION), and finally a quantitative target to conserve at least 20% (semi-)natural habitat in farmed landscapes to conserve biodiversity and to maintain a stable supply of crucial NCP^37,57^ (MULTI). Table 1 provides an overview of the different interventions and how they are combined across the alternative scenarios.

**Table 1:** Modelled scenarios and description of simulated land-system interventions

Land-system interventions		Modelled scenarios			
Action	Description	BAU	PROTECT	COACTION	MULTI
Area-based conservation	Concerted global area-based conservation action. By 2030, protected land area (currently about 15% of total land area) is enlarged by a total of 1.83 billion ha globally (plus 14.3% of total land area) in biodiversity hotspot areas (BH) and intact forest landscapes (IFL)^54^.	–	**✓**	**✓**	**✓**
Climate policy	Ambitious action for carbon uptake on land (NCP 4) that is consistent with the 1.5° target from the Paris Agreement. It adds nationally determined and carbon price-induced reforestation. We also assume a conservative expansion of bioenergy demand by 7 EJ per year in 2050, following nationally determined contributions (NDC).	–	–	**✓**	**✓**
Landscape policy	Retention and restoration of at least 20% (semi-)natural habitat by 2030 in farmed landscapes, in order to afford a stable supply of multiple important regulating NCP^57^ for sustainable production. (Semi-)natural habitats include forest, non-forest or grassland habitats that can maintain or restore native species diversity.	–	–	–	**✓**

Our scenario set is based on the SSP2 ‘middle-of-the-road’ storyline with moderate population and income growth. A ‘business-as-usual’ (BAU) scenario, including current national policies implemented (NPI), is contrasted with alternative scenarios that successively combine global interventions to promote area-based conservation, carbon uptake on land (‘climate policy’) and a quantitative target for landscape restoration (‘landscape policy’). See Methods for a more detailed account of the different measures. Climate impacts (e.g. on crop yields or water availability) were not considered here, as this was beyond the scope of this analysis.

Based on our modelling framework we find that the enlargement of protected areas and carbon-focused land restoration action alone would not reverse negative trends in landscape heterogeneity and the supply of key regulating NCP such as wild pollination, implying a continued biodiversity decline within cultured landscapes^20^. However, we also find that by spatially relocating cropland outside conservation priority areas, global action for climate and biodiversity conservation could be combined with efforts that promote landscape heterogeneity and key NCP without additional net carbon losses, primary land conversion or reductions in agricultural productivity. While we here assess how different land-system interventions could drive global land-use dynamics across different spatial scales, the underlying social, cultural and political conditions that would enable such action are not considered. Depending on the local context, the modelled interventions could also have important distributional consequences and should therefore not be pursued in isolation but as part of a broader sustainable transformation framework^55,58^.

## Results

### Projected demand for NCP-dependent land-based commodities

**Fig. 2:**
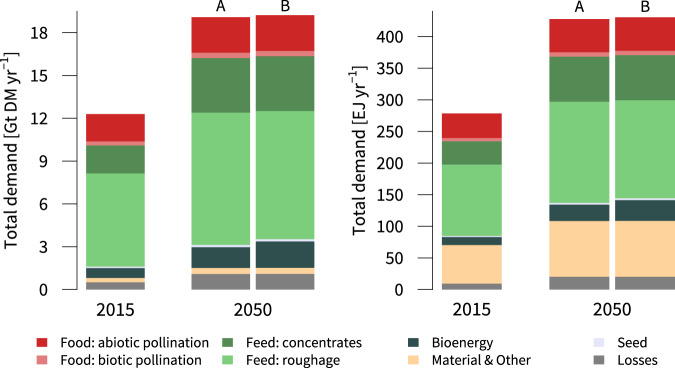
Demand for food, feed and material goods in 2015 and 2050 shown in both dry matter (DM) and energy content (EJ). (A) Future demand for agricultural products without land-based climate action in place (BAU & PROTECT); (B) demand after global measures for terrestrial carbon uptake in scenarios COACTION and MULTI were implemented (Tab. 1). Food and feed demand are calculated within MAgPIE’s internal food demand and livestock modules, respectively, based on socio-economic scenario drivers. Bioenergy demand in (A) is derived from the SSP2 ‘middle-of-the-road’ storyline for the land-use sector, while in (B) we assume a conservative expansion of bioenergy demand in accordance with countries’ nationally determined contributions (NDC).

Across all our scenarios, shifts in dietary patterns and population growth lead to considerable increases in the global demand for land-based products, such as food, feed, bioenergy and material goods, which critically depend on a stable supply of various regulating NCP^2,9^ (Fig. 2). By 2050, total demand for all land-based commodities increases by 54%, which is slightly raised further by the expansion in bioenergy demand in the COACTION and MULTI scenarios.

Global food demand expands by 35% between 2015 and 2050 across all scenarios, with the biggest increases in countries of Sub-Saharan Africa (+123%), Middle East & North Africa (+65%) and India (+56%). Furthermore, our scenarios include notable increases in the demand for food crops reliant on biotic pollination and with high nutritional value such as fruits, vegetables, nuts, and oil crops. Global demand for pollinator-mediated crops increases from 5.2 EJ yr^−1^ to 7.0 EJ yr^−1^ (+34%), with high disproportionate increases in Sub-Saharan Africa (+222.5%), Middle East & North Africa (+84.7%) and India (+62.9%) (Supplementary Fig. 1), which have seen a recent surge in under- and malnutrition^59^. Moreover, the demand for feed concentrates in our scenarios nearly doubles by 2050. Regional feed demand increases are highest in the developing and emerging countries of Sub-Saharan Africa (+172.0%), India (+157.9%), Asian countries excluding China and Japan (+87.5) and Middle East & North Africa (+69.4).

### Land-use and productivity changes (NCP 1 & 11–13)

Across all scenarios, we find considerable land-use transitions as a result of the growing demand for food crops, material goods and cost-effective carbon uptake on land. Increases in agricultural production are attained both by cropland expansion and investment into yield-increasing technologies (see Supplementary Methods), though with varying emphases across the scenarios. Cropland area in the BAU scenario increases by 439 Mha, while land-based climate mitigation in the COACTION and MULTI scenarios reduces cropland expansion into natural land by more than two thirds as compared to the BAU scenario. By contrast, the considerable enlargement of protected areas in the PROTECT scenario only slightly slows down cropland expansion at the global scale.

Similarly, variations in crop yield changes between the scenarios are a result of differing incentives to invest in yield-increasing technologies with and without climate action. However, future annual average crop yield increases across all scenarios never exceed the rate of yield change observed between 1995 and 2015 (1.6%). Annual average crop yield increases drop to 0.8% between 2015 and 2050 in the BAU and PROTECT scenarios and to 1.3% in the COACTION and MULTI scenarios (Supplementary Fig. 9). Even though we find that our landscape policy itself leads to a spatial relocation of cropland areas outside conservation priority areas, it does not cause additional cropland expansion at the global scale nor does it incentivise further yield increases—an outcome that is robust to variations in important exogenous model parameters, such as trade liberalisation, investment costs for increasing agricultural productivity and carbon price (see Supplementary Fig. 18).

**Fig. 3:**
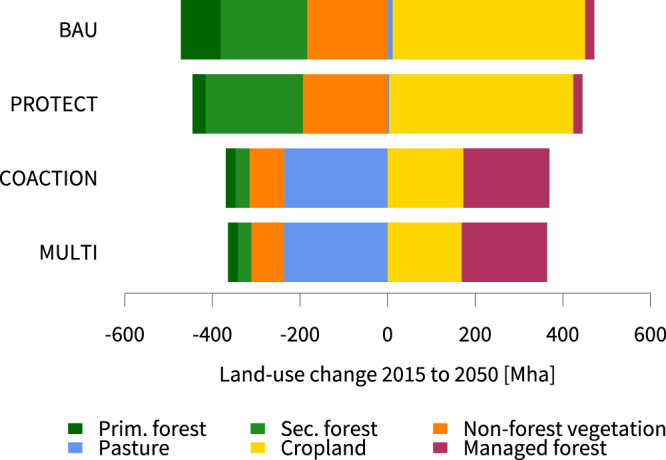
Global projections of land-use change between 2015 and 2050. For the reference year 2015, global land cover includes 1654 Mha of cropland (food, feed and bioenergy crops), 3202 Mha of pasture area, 3979 Mha of forest and 3930 Mha of non-forest vegetation. Managed forest features forest restoration based on NDCs and carbon price-induced reforestation, as well as timber plantations. For NDC and carbon price-induced reforestation we employ the same growth curves and carbon densities as for native ecosystems (see Methods and Supplementary Methods).

Losses of primary and secondary forest, as well as non-forest ecosystems (other land) are substantial in the scenarios without climate action (Fig. 3). These losses are mainly due to cropland expansion in Sub-Saharan Africa and Latin America. Natural land conversion is only marginally reduced in the PROTECT scenario as compared to the BAU scenario. We also find that in PROTECT reduced primary forest losses (30 Mha as compared to 91 Mha in the BAU scenario) are compensated by higher losses in secondary forest and other land, as a result of leakage effects in Latin America and Sub-Saharan Africa. In contrast, expansion into all natural lands is drastically curbed in the COACTION and MULTI scenarios and the loss of primary forest area is halted at 22 Mha after 2030 in both scenarios. Hence, spatial cropland relocation as a result of our landscape policy would not lead to additional losses of primary forest land. In the MULTI scenario, we even find slightly reduced global losses in secondary forest (−1 Mha as compared to COACTION) and other land areas (−5 Mha as compared to COACTION) (Supplementary Figs. 15,17). The marked reductions in the conversion of natural land in the COACTION and MULTI scenarios, however, come at the expense of significant reductions in pasture and rangeland areas in China, Sub-Saharan Africa and Latin America (Supplementary Fig. 16). Yet, these reductions in pasture and rangeland area are not only a result of cropland expansion, but also driven by a marked growth in restored forest area for land-based carbon uptake. The required areas for forest restoration in the COACTION (196 Mha) and MULTI (195 Mha) scenarios, however, still remain well below the estimated feasible boundary of 500 Mha for global forest expansion^60^.

### Carbon uptake and losses from land-use change (NCP 4)

**Fig. 4:**
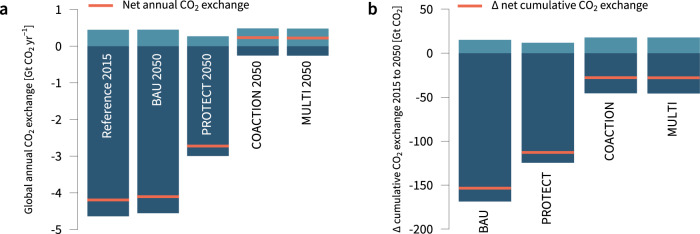
Projected carbon uptake and losses from land-use change. **a** Global annual CO_2_ exchange in 2015 as well as 2050 and **b** cumulative CO_2_ exchange caused by land-use change between 2015 and 2050. Negative values (dark blue) indicate global carbon losses to the atmosphere in terms of CO_2_ from the conversion of pasture, forest and non-forest ecosystems, while positive values (light blue) show carbon uptake from regrowth due to land abandonment and reforestation. Orange lines depict the net CO_2_ exchange between 2015 and 2050.

Regarding carbon uptake and losses, we focus on net CO_2_ exchange from land-use change, which includes carbon losses to the atmosphere (soil, litter, vegetation) from the conversion of natural land, and carbon uptake from reforestation and vegetation regrowth on abandoned agricultural land (see Methods & Supplementary Methods). We find the largest differences in land-use change-related CO_2_ exchange between the BAU, PROTECT and COACTION scenarios (Fig. 4). These differences largely reflect the considerable variation in the loss of (carbon-rich) forest and non-forest ecosystems and in the amount of climate policy-driven reforestation between the scenarios. In the BAU scenario, annual net carbon losses barely change between 2015 and 2050 (−2%). Biodiversity-focused area-based conservation leads to a drop of 34% in carbon losses as compared to the BAU scenario. This drop is significant considering that the difference in the loss of natural land area between the BAU and PROTECT scenarios is only 6%, underlining important synergies between the protection of biodiversity-rich ecosystems and climate change mitigation. In the COACTION scenario, the land system is converted to a net carbon sink due to drastic reductions in cropland expansion and substantial reforestation. Despite shifts in spatial land-use patterns as a result of our landscape policy, we also find that additional carbon losses in the MULTI scenario are insignificant. Net carbon uptake in 2050 is only marginally higher in the COACTION scenario (233 Mt CO_2_ yr^−1^), as compared to the MULTI scenario (223 Mt CO_2_ yr^−1^). This outcome also holds against variations in crucial exogenous model parameters (see Supplementary Fig. 19).

### Repercussions at the landscape scale (NCP 1, 2, 8, and 10)

In this study, we consider one meso-scale (55 km ×55 km at the equator) and two field-scale (300 m × 300 m at the equator) indicators for changes in the supply of key regulating NCP in farmed landscapes. At the meso-scale we report cropland fractions in terms of total available potential cropland to assess changes in compositional landscape heterogeneity. At field-scale, we derive pollination sufficiency scores^36^ based on the area of (semi-)natural habitat surrounding every agricultural pixel, which in a range of studies has shown to reliably predict wild pollination^22,50,61,62^. These serve both as a direct global proxy for changes in wild pollination supply and in configurational landscape heterogeneity, which, beyond pollination supply alone, is associated with benefits for other important NCP^8,63,64^ and biodiversity conservation^20,37^. In addition, we estimate field-scale changes in soil loss by water erosion as a measure for soil degradation^65^, based on the Global Soil Erosion Modelling (GloSEM) platform^66,67^. For further information on how the indicators are derived, see Methods.

#### Compositional landscape heterogeneity

**Fig. 5:**
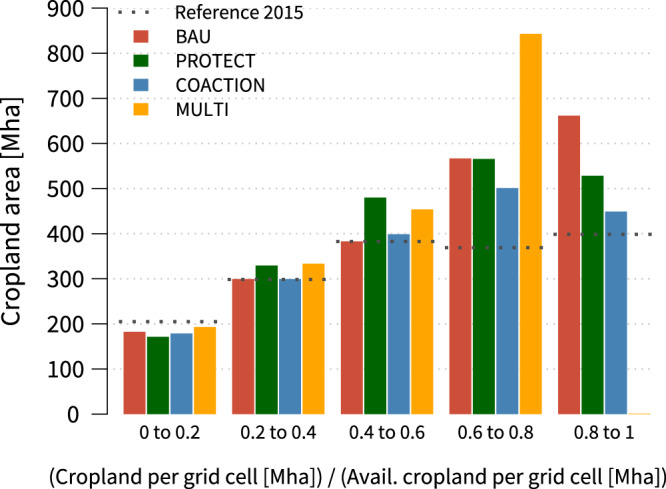
Projected cropland fractions in terms of available potential cropland at the 0.5 degree grid-cell level. We applied a conservative upper default constraint to cropland expansion per grid-cell of 90% of the theoretically available cropland, as commonly remaining areas are lost to field margins, roads etc.. Cropland shares in the input data could still in some cases be higher.

Our results indicate that in 2015, 399 Mha (24%) of global cropland are situated in comparably homogeneous grid-cells with high cropland fractions between 0.8 to 1 (Fig. 5). In 2050, landscape homogenisation further increases in the BAU, PROTECT and COACTION scenarios, though to varying degrees. The BAU scenario shows the strongest reductions in compositional heterogeneity across all scenarios, as cropland area in homogenised grid-cells increases to 662 Mha (+66%). Changes in compositional heterogeneity, by contrast, are halved in the PROTECT scenario (+33%) and are substantially lower in the COACTION scenario (+13%). In the MULTI scenario cropland fractions at the grid-cell level remain at less or equal 0.8, because of our landscape policy intervention.

#### Pollination supply and configurational landscape heterogeneity

**Fig. 6:**
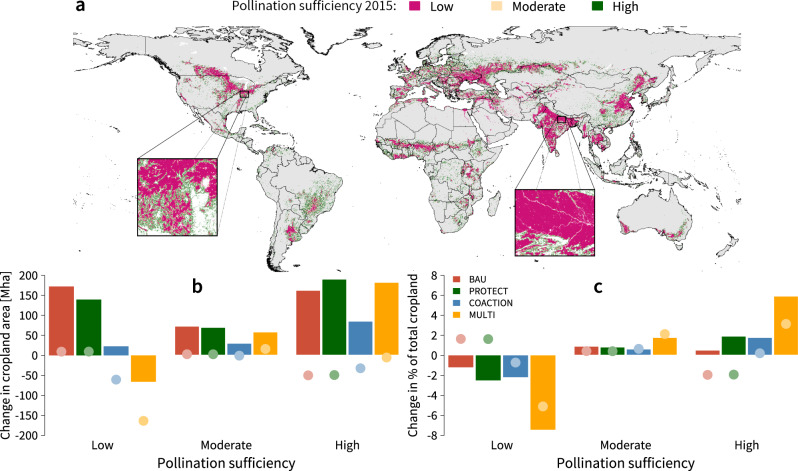
Pollination sufficiency estimates and global changes across modelled scenarios. **a** Spatial representation of estimated pollination sufficiency values (see Methods) at field-scale for the reference year 2015. ‘High’ indicates that there is sufficient pollinator habitat available within the 2 km flight radius around cropland pixels to ensure a stable pollination supply, while ‘moderate’ and ‘low‘ indicate deficient or insufficient availability of pollinator habitat around cropland. Grey pixels denote non-cropland areas. **b** Global changes in cropland area with ‘high’, ‘moderate’ and ‘low’ pollination sufficiency scores between 2015 and 2050. Bars represent overall changes in cropland area, while shaded dots illustrate pollination sufficiency changes in historic cropland areas only (pixels classified as cropland in 2015). **c** Global changes across pollination sufficiency thresholds expressed as percentage of total cropland. See Supplementary Fig. 4 for changes in absolute values.

To facilitate the presentation of our results, we have grouped all pollination sufficiency scores into three categories of ‘low’ (0–0.33), ‘moderate’ (0.33–0.67) and ‘high’ (0.67–1) values. We find that in 2015 nearly half of total cropland (915 Mha) only reaches low pollination sufficiency scores, while merely about a third (687 Mha) of all cropland area attains high pollination sufficiency (Fig. 6a, Supplementary Fig. 4). By 2050, cropland area within simplified landscapes and low pollination sufficiency scores further increases across the BAU, PROTECT and COACTION scenarios. However, we find that these increases are considerably smaller in the COACTION scenario. As a result of our landscape policy, the MULTI scenario is the only scenario, in which we find a clear reduction in cropland areas with low pollination sufficiency scores over time (Fig. 6b). Yet, even in the MULTI scenario 849 Mha of cropland are still characterised by low pollination sufficiency scores (Supplementary Fig. 4). Cropland area with high pollination sufficiency scores, on the other hand, increases across all scenarios. However, in the BAU, PROTECT and COACTION scenarios, these increases are largely attained by the expansion of cropland into pasture and natural land areas at the agricultural frontier, which drives carbon and soil losses (see below). This is further underlined by pollination sufficiency changes in historic cropland areas (Fig. 6b). In fact, for the BAU, PROTECT and COACTION scenarios, 51, 50 and 34 Mha of historic cropland areas drop out of the highest pollination sufficiency class (Fig. 6b, shaded dots). In the MULTI scenario, by contrast, pollination sufficiency values in historic cropland areas only show a marginal net reduction. The overall dynamics of our pollination sufficiency estimates are also robust to the omission of grassland areas from the definition of semi-natural habitats (Supplementary Fig. 20).

#### Soil loss by water erosion

**Fig. 7:**
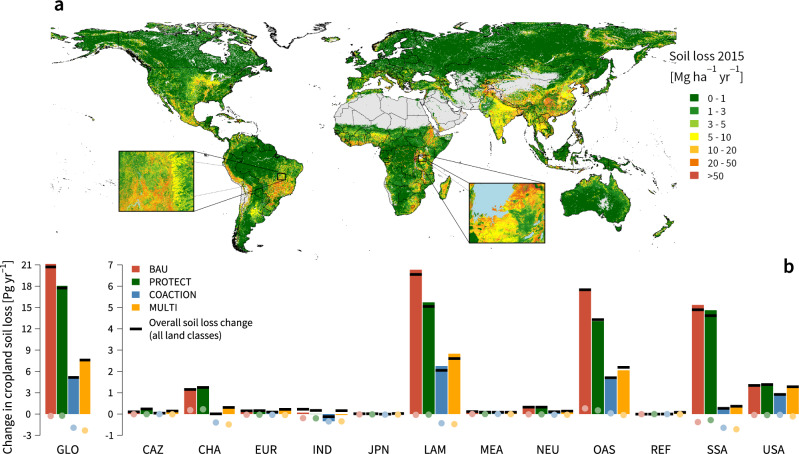
Global estimated rates of soil loss by water erosion and global changes across modelled scenarios. Panel **a** depicts spatial estimates of global soil loss (see Methods) divided into seven classes according to the European Soil Bureau classification. The colour gradient denotes the intensity of soil loss from low (green) to high (red) rates of soil loss. Grey areas are not covered by our model due to the lack of data. **b** Projected changes of soil loss on cropland as compared to 2015 aggregated to global (left) and regional values (right). Bars show overall soil-loss changes, while shaded dots only show soil-loss changes in historic cropland areas. Overall soil-loss change (black line) between 2015 and 2050 closely follows soil loss on cropland. GLO: Global; CAZ: Canada, Australia and New Zealand; CHA: China; EUR: European Union; IND: India; JPN: Japan; LAM: Latin America; MEA: Middle East and Northern Africa; NEU: non-EU member states; OAS: other Asia; REF: reforming countries; SSA: Sub-Saharan Africa; USA: United States. See Supplementary Fig. 5 and Supplementary Fig. 6 for values of absolute soil loss.

Estimated soil loss changes in this study are solely derived from land-use/land-cover changes. Other factors that might vary over time and impact soil loss, such as climate-change induced rainfall changes or soil conservation practices were held constant. For our reference year 2015, we estimate that total global soil loss by water erosion amounts to 44 Pg yr^−1^, of which about half comes from soil loss in cropland areas (Supplementary Figs. 5, 6; Supplementary Table 2). These estimates are well in line with other studies that used the same methodology, but alternative land cover and vegetation cover data^66,67^. Soil loss changes in our future scenarios are strongly driven by the divergent patterns of cropland expansion. Hence, the BAU scenario shows the highest increases in soil loss by 2050 (+21 Pg yr^−1^; Fig. 7b). In terms of the land-system interventions, our model results reveal strong synergies between ambitious climate action and soil protection at the global scale (−16 Pg yr^−1^ as compared to BAU). The reason is both a considerable smaller total cropland area and a higher share of second-generation bioenergy crops that provide increased soil cover within croplands, such as bioenergy grasses and short rotation coppice. Synergies with area-based conservation action are far less pronounced and mainly driven by a reduced cropland expansion in tropical areas sensitive to water erosion (−3 Pg yr^−1^ as compared to BAU). However, our results also show that our landscape policy intervention could lead to higher overall soil loss as compared to the COACTION scenario, despite clearly reducing soil loss in historical cropland landscapes,. The reason is that cropland areas shift to areas with a higher susceptibility to water erosion, as a result of the restrictions at the landscape scale. Regional soil-loss changes are closely linked with regional shifts in cropland area. We find the highest soil-loss changes in Latin America, Asia, Sub-Saharan Africa and the United States (Fig.5b; Supplementary Fig. 8). However, there also are high synergies between soil protection and climate action in Sub-Saharan Africa and China, where in 2050 soil loss only marginally increases in the COACTION and MULTI scenarios (Fig. 7b).

## Discussion

This study provides an integrated and globally-consistent perspective on how, over the coming three decades, increased demand for food and material goods, as well as large-scale actions targeted at climate and biodiversity protection, could drive changes in landscape heterogeneity and key regulating NCP that sustain land productivity. Consistent with historic trends and previous studies^53,68^, we here find considerable global increases in the appropriation of material NCP to meet the growing demand for food and other material goods. Sustainably fulfilling this demand in the future will depend on intact (agro-)ecosystems and a stable supply of a broad range of critical regulating NCP within agricultural landscapes^2,18,20,37^, such as pest control, pollination, climate regulation, or soil protection and regeneration. Yet our results indicate that, mediated through land conversion and landscape-scale changes, the unilateral expansion in the appropriation of material NCP could have significant repercussions on such crucial regulating NCP and reinforce historic^2,4,9^ negative trends. In particular, we find that without dedicated interventions, both landscape simplification and soil degradation could continue in the future with negative implications for the long-term productivity and resilience of farmed landscapes. Noticeable increases in pollination supply and landscape heterogeneity also found across the BAU, PROTECT and COACTION scenarios were realised through cropland expansion at the cost of pasture and natural ecosystems at the agricultural frontier, increased carbon losses, and higher rates of soil loss.

Our findings also show that interventions, such as the considerable enlargement of protected areas and ambitious carbon policies in the land sector, could be augmented by interventions that promote landscape heterogeneity to provide co-benefits beyond climate regulation and protection of biodiversity in conservation priority areas. Notably, our models indicate that conserving 20% of (semi-)natural habitat in farmed landscapes globally does not generate trade-offs of additional conversion of forests and other natural vegetation, carbon losses from land-use change or additional investment into yield-increasing technologies. While our results show that promoting landscape heterogeneity does not lead to changes in the overall amount of cropland at the global scale, we find that it does require cropland relocation. Therefore interventions that increase landscape heterogeneity must be, on the one hand, embedded in concerted efforts that prevent potential cropland expansion into ecologically sensitive areas and, on the other hand, accompanied by policies that compensate for the distributional consequences of such measures, including framework conditions that allow for dedicated investments into the management of natural capital and the conservation of crucial NCP. Such structural changes could also help local communities to directly benefit from conservation and reforestation actions^69,70^. Changes in spatial cropland patterns that result from our landscape policy intervention could, moreover, lead to slightly higher overall soil losses by water erosion that are not fully compensated by the considerable reduction of soil loss in historical cropland areas as a result of the intervention. The widespread implementation of soil conservation practices could alleviate some of these side-effects^66^ and address other aspects of soil degradation not covered in this study, such as soil organic matter depletion, soil compaction or wind erosion, to sustain the capacity of soils to provide essential NCP^71^.

Similar to previous studies, we also find that measures to curb expansion into natural land for carbon and biodiversity protection would require productivity increases in remaining cropland areas^10,12^. Closing yield gaps^44,72^, more efficient land management^73^ and ecological intensification^74^ are potential routes for achieving productivity increases. However, these measures demand a careful consideration of their repercussions regarding people, biodiversity and NCP in different landscape contexts^75–78^, which could not be examined here. In the past, the shift from low-input farming to intensive farming and concurrent landscape transitions have caused sharp species declines^35,79,80^ and undermined essential NCP^2,4,8,9,13^. Promoting off-farm ecosystem management and multifunctionality within farmed landscapes therefore could not only offer decisive means for counteracting these negative trends^18,37,57,81,82^, but also reduce the use of external inputs in agricultural production, such as pesticides^83,84^. Increased landscape heterogeneity would also provide co-benefits for biodiversity at larger scales^20,37^ and increase the effectiveness of protected areas (PAs). Globally, only 10% of terrestrial PAs are currently structurally connected^85^ and this lack of connectivity in an increasingly hostile ‘matrix’ could lead to further species declines, even within PAs^86,87^. Climate-change impacts on terrestrial ecosystems could further expose this lack of connectivity, as species will find it harder to migrate along environmental gradients^88,89^.

Conserving higher amounts of (semi-)natural habitats in farmed landscapes provides multiple benefits and supports biodiversity conservation, but the quality and the effectiveness of this measure strongly depend on the local context. The management of these habitats, therefore, should depend on stakeholder preferences and adaptive ecosystem management within different landscape contexts. This could also entail dedicated management to support the conservation of locally rare or threatened species rather than a sole focus on NCP supply and open up opportunities to conserve traditional land-use practices, such as extensive grazing^57^.

Meeting the ambitious climate and biodiversity protection targets at the global scale could entail considerable land-system transformations, while the ramifications can vary regionally. For example, large-scale forest restoration efforts in line with the targets of the Paris Agreement would require to reduce the global pasture and rangeland area by 7.4% as compared to 2015, particularly in China, Latin America and Sub-Saharan Africa. These transformations, however, could have important local socio-economic and cultural consequences, which should be addressed by wider sustainable development and structural change policies^55,58^. Extensively managed grasslands can also harbour distinct communities of species and the loss of ancient grassland areas could not only impact local biodiversity but also flyways of migratory birds^90,91^. Reforestation should therefore prioritise areas first where it is in line with other land conservation targets, such as restoring tropical forests.

Our modelling framework depends on a wide range of socio-economic and biophysical information across multiple spatial scales and the uncertainty of different fine-scale global data products, in particular, has recently been scrutinised^92^. However, the dynamic land-allocation processes that drive our scenario results have shown to be robust to changes in central model assumptions both in this study (see Supplementary Figures) as well as in previous studies^58,93^. This is also confirmed by our soil loss estimates, which match well with earlier studies that have applied the GloSEM platform despite using alternative land cover and vegetation cover data sets^66,67^. The models employed in this study have, moreover, been intensely validated. MAgPIE results, for example, are evaluated at regional and global scale using a validation database that includes historical data for most model outputs^41,94^ (see Supplementary Figures), while the SEALS model is empirically calibrated using a loss function that compares modelled outputs with observed fine-scale land-use patterns^28^. The GloSEM platform, furthermore, has been carefully evaluated using a cross-comparison with empirical data combined with a sensitivity analysis^66^.

Assessing processes like pollen transfer at the global scale obviously involves broad simplifications, bypassing different local contexts. However, parsimonious approaches for estimating pollination supply have not only proven to be useful across different spatial scales, but also effective in capturing different plant-pollinator relations^36,61^. A range of earlier studies have consistently found a positive relationship between the amount of non-cropland habitat and pollination supply in agricultural landscapes at the global scale^8,20,50^. This is because, by and large, wild pollination is provided by generalist species that can adapt to a wide range of environmental conditions^95^. In addition, reductions in non-cropland habitat have also shown to negatively affect pollinator richness more widely, which, independently of the dominance-effect, has also been found to drive pollination supply across various landscape contexts^8^. This empirical basis supports the reliability of our outcomes regarding pollination supply in different landscape contexts.

While our global modelling framework takes a step forward by integrating different spatial scales and assessing multiple NCP dimensions in face of important sustainability questions, it is subject to several limitations.

First, land-cover allocation at field-scale was largely driven by current land-use patterns. Therefore, our estimates with regard to the provisioning of crucial NCP may constitute a lower bound and the effects for both NCP supply and biodiversity could be even further improved by targeted ecosystem management and integrated spatial planning that considers areas that matter most, e.g. due to their ecological importance or due to their proximity to production sites that depend on NCP supply^63^.

Secondly, in this study the quality of semi-natural habitats in agricultural landscapes is only characterised in a simplified manner (grassland, forest and non-forest vegetation). However, the amount of carbon stored in patches of semi-natural vegetation might vary depending on different management practices and spatial arrangement. In particular, we did not consider how edge effects might influence carbon stocks in patches of semi-natural vegetation^96^. Yet, evidence suggests that carbon stored in semi-natural vegetation, such as hedgerows, is on average comparable with forest vegetation^97^.

Thirdly, the effects of introducing second-generation bioenergy crops could only be considered in our soil loss estimates, but not with regard to landscape structure and pollination, because of model limitations. Yet there is evidence that these could generate significant co-benefits in farmed landscapes^98^.

Lastly, we could not consider feedbacks related to changes in NCP supply here, such as crop productivity changes as a result of mismatches between pollination demand and supply or due to soil loss. Even though this has already been exemplified in other contexts^28,99^, representing those feedbacks in modelling frameworks with a dynamic decision-making process remains challenging, opening up promising opportunities for future research.

Despite these limitations, the results of our study emphasise the importance of integrating multiple spatial scales in global-scale analyses. The landscape perspective, which we have added in this study, is particularly important for understanding how future land-use changes could affect the supply of crucial regulating NCP, which sustain land productivity, the resilience of (agro-)ecosystems and a good quality of life. Reconciling different spatial scales also strengthens decision-making in the land sector at national and international levels and allows for improved outcomes of global-scale actions targeted at climate-change mitigation and tackling the biodiversity crisis.

## Methods

### Model descriptions

#### MAgPIE

The Model of Agricultural Production and its Impact on the Environment (MAgPIE) is a global, multiregional land-system model, developed to assess global land-use dynamics and their associated consequences for sustainable development until the end of the 21st century^41,43^. It uses a recursive dynamic cost optimisation approach under biophysical and socio-economic constraints to spatially allocate production to meet the demand for food, feed, bioenergy and biomass for other material uses. Dominant cost types include factor requirement costs (capital, labour, fertiliser etc.), land conversion costs, transportation costs to the closest market, investment costs for increasing agricultural productivity (technological change) and costs for irrigation. Spatially explicit biophysical constraints are derived from simulated data from the dynamic global vegetation, crop and hydrology model LPJmL at a spatial resolution of 0.5 degrees (latitude/longitude) driven by a historical climate data time series^100^. Carbon stocks (vegetation, litter and soil) and natural water availability are simulated using LPJmL4^48,49^ whereas crop irrigation water requirements and yield patterns (crop and grassland) are derived using LPJmL5 simulations^46,47^ with unlimited N supply. The input data at 0.5 degree resolution are clustered following the approach described by Dietrich et al.^101^ and averaged using a smooth spline over the simulated time series^102^. To match crop-specific FAO production levels at the regional scale, averaged yield patterns are calibrated at the cluster scale. The area potentially suitable for cropland was derived from Zabel et al.^103^ and adjusted by excluding the lowest tertile (suitability index <13) of marginal land (suitability index between 0 and 33). Socio-economic constraints such as trade patterns and interest rates are defined at the scale of twelve model regions, in which large economies are resolved individually, while smaller economies are grouped together. MAgPIE represents all major crop and livestock types, non-food agricultural commodities, as well as supply chain losses. Land competition is based on cost-effectiveness between crop and livestock production and land-based climate-change mitigation options. Food demand projections are estimated based on population growth, change of demographic structure and per-capita income. The food demand model^104^ (Fig. 1) draws on anthropometric and econometric approaches to determine the distribution of undernutrition, overnutrition and obesity, as well as body height by country, age-cohort, and sex. It also separates food waste and food intake of four major food items: staple calories, animal-source calories, calories from fruits, vegetables and nuts, as well as empty calories. All elasticity parameters of the food demand model are derived from past observed data. Greenhouse gas emissions from land use include carbon and nitrogen-related emissions from managed soils and animal waste management. Nitrogen-related emissions are estimated based on a nitrogen budget approach^105^, while carbon exchange with the atmosphere is calculated as the difference in carbon stocks due to land-cover changes between simulated time steps. This model version also accounts for depreciation and new investments in capital stocks for crop production in each time step. This setting favours locations where crops have historically been grown, improving spatially-explicit crop-related outputs and slowing down the free relocation of production to locations with better climatic conditions but with no existing infrastructure.

#### SEALS

We applied the SEALS (Spatial Economic Allocation Landscape Simulator) model to spatially allocate projected land cover changes in MAgPIE at 0.5 degrees to a resolution of 10 arc seconds (field-scale), based on adjacency relationships, physical suitability and conversion eligibility. The starting condition of the model landscape is defined by a high-resolution land use/land cover (LULC) map from the European Space Agency’s Climate Change Initiative (ESA-CCI) for the year 2015. The 37 ESA-CCI land-cover classes were grouped into seven functional types, including cropland, grassland, forest, non-forest vegetation (e.g. shrub land or herbaceous cover), urban, barren land, and water. We then generate maps that describe the strength of the spatial adjacency for each functional type on nearby land cover pixels, accounting for both distance and the agglomeration of pixels (see Supplementary Methods). Physical suitability for land-cover allocation is determined by combining high-resolution soil organic carbon (SOC) data from Hengl et al.^106^ and topographic information. The topographic information is generated by processing data from a digital elevation model (DEM) to obtain a terrain roughness index (TRI)^107^. We also apply allocation constraints, in order to make sure that no further cropland expansion can occur, e.g. if pixels are water or urban land. Adjacency relations, physical suitability, and conversion eligibility are then combined into an overall suitability map and all pixels are ranked depending on their suitability. Land-cover changes are finally allocated in an iterative process to the highest ranked grid cells, until all projected land-cover changes from MAgPIE between the 2015 and 2050 are allocated, in order to prepare high-resolution land-cover maps for each of the modelled scenarios. The specific coefficients for the different adjacency relationships and physical suitability were found by iteratively applying the above allocation algorithm on a time-series (2000–2010) of LULC maps from ESA-CCI and selecting the coefficients (Supplementary Table 7) that were most predictive on withheld data (2011–2015).

### Fine-scale NCP indicators

#### Pollination sufficiency

The presence of pollinator habitat around farmland has shown to be a reliable indicator for wild pollination of crops^8,22,50^. We therefore determine the area of pollinator habitat within foraging distance of cropland areas to derive pollination sufficiency values, following Chaplin-Kramer et al.^36^. We define pollinator habitat as any (semi-)natural land cover in farmed landscapes, i.e. forest, non-forest and grassland vegetation cover^57^. Pollination sufficiency is then defined by two factors: i) the proportion of pollinator habitat within a 2 km flight radius of every cropland pixel, which corresponds well to the foraging distance commonly found in wild pollinator communities^50,62^ and ii) a sufficiency threshold of 30% pollinator habitat to assess whether there is sufficient pollinator habitat within the 2 km flight radius to sustain a stable wild pollination supply. The threshold is derived from a range of empirical studies that have shown that pollination supply can be reliably predicted from the area of (semi-)natural habitat around cropland^22,50,61,62^. Based on these criteria we rank all cropland pixels to values between 0 and 1, where 1 indicates a share of >30% pollinator habitat within the 2 km radius around cropland pixels and values between 0 and 1 relate to a proportional area between 0 and 30%. All fine-scale spatial data analysis was performed in R^108^ by employing the packages ‘terra’^109^, ‘exactextractr’^110^, ‘foreach’^111^ and ‘doParallel’^112^.

#### Soil erosion

Our methodology to estimate land-use induced soil loss builds upon the Global Soil Erosion Modelling (GloSEM) platform established and validated by Borelli et al.^66,67^. GloSEM is based on a large-scale Geographical Information System (GIS) version of the empirical, detachment limited Revised Universal Soil Loss Equation (RUSLE) model. We use GloSEM to estimate the long-term annual soil erosion rates at field-scale (300 m × 300 m at the equator), expressed as a mass of soil lost per unit area and time (Mg ha^−1^ yr^−1^). As compared to more complex process-based models, which require fine-scale input data that are not yet consistently available at the global scale, RUSLE-type modelling approaches provide a simple yet physically plausible means to predict soil erosion resulting from sheet and drill erosion processes at large scales. RUSLE type models have generally shown to produce reasonably accurate estimates of soil loss for most practical purposes and policy applications. GloSEM applies the same principles as other RUSLE-type models and includes a driving force (rainfall erosivity), a resistance term (erodibility of the soil), as well as topographical and land cover information. Global rainfall erosivity (R) values were derived from the Global Rainfall Erosivity Database (GloREDa) and interpolated by employing a Gaussian process regression (GPR) approach using covariates from the WorldClim database^113^, as detailed in Panagos et al.^114^ and Borelli et al.^66^. Soil erodibility (K) was estimated by algebraic approximation^115^, while the soil properties were derived from the ISRIC SoilGrids database^116^ and following Borrelli et al.^66^. The topographical parameter (LS) was calculated by processing DEM data following the two-dimensional GIS-based approach presented by Desmet and Govers^117^. In order to estimate the land cover and management factor (C-factor), we employed differing approaches for cropland, forest and non-forest vegetation cover. For cropland we used spatial cropping patterns from MAgPIE at 0.5 degree level and assigned C-factor values to the 20 crop groups (Supplementary Table 8) represented in MAgPIE according to literature values. We then calculated an area weighted mean between all crop groups in each 0.5 degree grid cell to derive overall C-factor values, which were disaggregated to 10 Arc seconds. C-factor values in non-cropland areas were estimated by combining C-factor values for forested and non-forested areas from the literature with information on annual vegetation and forest cover for each land cover pixel. In order to obtain global mappings of annual potential vegetation and forest cover, which also cover degraded pixels in the initial time step, we used separate random forest models (R package ‘ranger’^118^) based on global FCOVER^119^ (fraction of green vegetation cover) data from the Copernicus Global Land Service^120^ and tree cover data from Hansen et al.^121^ (Supplementary Figs. 34 & 35). The covariates for these mappings were derived from the WorldClim data base^113^. Further specifics on how the C-factor has been derived are given in the Supplementary Mtehods.

### Scenario set-up

The guiding principle of our scenario design is the assessment of landscape-scale effects of global efforts targeted at biodiversity, climate and landscape protection. We therefore contrast a business-as-usual (BAU) scenario with an alternative scenario set that successively combines global targets for the enlargement of protected areas and land-based climate mitigation, as well as a quantitative target to sustain at least 20% of (semi-)natural habitat at the landscape scale (Table 1). Our scenario design rests upon the SSP2 ‘middle-of-the-road’ storyline for the land-use sector^53^ with continued trends of moderate population and income growth and a representation of current national policies implemented (NPI). In the following, we offer a more detailed description of the biodiversity, climate and landscape protection measures:

- *Area-based conservation*. Our template for area-based conservation focuses both on reactive and proactive conservation. The reactive component includes areas that are highly vulnerable to land expansion and require rapid conservation action. In order to map this reactive component, we use information on biodiversity hotspots from Conservation International (CI). Biodiversity hotspots harbour nearly 43% of the world’s bird, mammal, reptile and amphibian species and more than half of the world’s plant species as endemics, while, at the same time, they are characterised by a loss of native habitat by >70%^122^. Focal areas in Latin America are the Atlantic Forest, the Cerrado, large parts of Mesoamerica, the Andes as well as the Chilean Forests, while in Africa they cover the Horn of Africa, Madagascar and the Guinean Forests of West Africa. The proactive conservation template, on the other hand, identifies large (>500 km²) areas of intact forest vegetation, that have so far evaded marked human alteration. The mapping of this component is based on the Intact Forest Landscapes (IFL) data^123^. Intact forest landscapes cover the Amazon and Congo basin in Latin America and Africa as well as a considerable share of the boreal forests in North America and Asia. We combined the reactive and proactive conservation templates into an overall spatial conservation template (Supplementary Fig. 23). This conservation template was gradually put into effect during the time steps between 2020 and 2030, so that full protection was reached after 2030 and no further land conversion could occur within areas covered by the conservation template. Remnant global primary forest areas already saw full protection by 2025.

- *Climate policy*. Climate policy in our study is aimed at ambitious climate change mitigation in the land system and formulated as such that it is broadly consistent with the 1.5 °C target from the Paris Agreement. It pertains to a universal carbon price, reforestation and a moderate expansion of bioenergy demand. Carbon uptake and losses from land-use change are multiplied with the carbon tax to determine the carbon emission costs, which are included in the objective function (cost-minimisation) of MAgPIE. It therefore becomes less attractive for the model to convert carbon rich forest or non-forest ecosystems^10^. Moreover, reforestation is rewarded by multiplying the expected carbon dioxide removal over a 50-year planning horizon with the future carbon price, discounted to present value and adjusted by an annuity factor to obtain average annual rewards. Forest growth in MAgPIE is modelled following the approach of Humpenöder et al.^124^ but with updated parameters for the Chapman-Richards growth function for native vegetation and timber plantations from Braakhekke et al.^125^ (see also Strefler et al.^126^). For this study we chose to use the same growth curve for NDC and carbon-price induced reforestation as for native vegetation, as opposed to plantation growth curves that would reflect assisted regrowth with non-native species. We also apply the same carbon density for NDC and carbon-price induced reforestation as for native ecosystems (see Supplementary Methods). Effectively, reforestation sites are therefore assumed to have the same carbon density and consist of the same species that could be found during the succession of native vegetation. Carbon-price induced reforestation is moreover constrained to areas outside the boreal zone and where the carbon density of the potential natural vegetation is >20 t C ha^−1^. This restoration-based approach to land-based climate change mitigation thus provides important synergies regarding the conservation of local biodiversity. Here, we therefore consistently use the term reforestation, although afforestation and reforestation have often been used interchangeably. The carbon tax starts at a level of 109.8 USD per ton of CO2 in 2025 and nonlinearly increases to 371.8 USD per ton of CO2 in 2050 at an annual rate of 5%. The carbon price trajectory was derived from coupled REMIND-MAgPIE runs^127,128^ (Supplementary Fig. 27). REMIND is a global economy and energy system model, which can be run in an iterative soft-coupled mode with MAgPIE to estimate consistent bioenergy demand and carbon prices across the global economy, energy and land-use system^55^. In addition to carbon price driven reforestation, we also include reforestation schemes and the expansion of bioenergy demand by 7 EJ per year in 2050, as specified in nationally determined contributions (NDC).
- *Landscape policy*. In order to address landscape simplification in the global land system and to sustain critical regulating NCP in farmed landscapes, we constrain cropland expansion in MAgPIE to a maximum of 90% of the actually available cropland per grid-cell in 2025 and to maximum of 80% by 2030 (linear decrease). This threshold corresponds to evidence that integrating at least 20% permanent (semi-)natural habitat into farmed landscapes promotes resource diversification, connectivity and stability of population dynamics and is therefore crucial for safeguarding biodiversity and a stable supply of a wide range of important regulating NCP such as pollination, pest control, hydrology, climate and air quality regulation^37,57^.

### Reporting summary

Further information on research design is available in the Nature Portfolio Reporting Summary linked to this article.

## Supplementary information

Supplementary Information

Peer Review File

Reporting Summary

## Data Availability

All model results presented in this paper have been archived via Zenodo and can be accessed under 10.5281/zenodo.7804740^129^.

## Code availability

The model code of the MAgPIE model is openly available under the GNU Affero General Public License, version 3 (AGPLv3) and accessible via GitHub (https://github.com/magpiemodel/magpie). The release version (MAgPIE 4.3.5.), on which this study is based, has been archived via Zenodo (10.5281/zenodo.5394196^130^). MAgPIE 4.3.5. is accompanied by a technical model documentation (https://rse.pik-potsdam.de/doc/magpie/4.3.5/), which has been compiled with the GAMS code documentation toolkit goxygen^131^. The SEALS code has also been archived via Zenodo and can be accessed via 10.5281/zenodo.7795957.
