# Supplementary material for: Projected landscape-scale repercussions of global action for climate and biodiversity protection
Source: Nat Commun. 2023 May 16;14:2515. doi: 10.1038/s41467-023-38043-1 (PMC10188494; doi:10.1038/s41467-023-38043-1)
Supplement: Supplementary file 1 — Supplementary Information [file 41467_2023_38043_MOESM1_ESM.pdf]

Supplementary Information for

Projected landscape-scale repercussions of global action for climate and biodiversity protection

Patrick von Jeetze[1,2,*], Isabelle Weindl[1], Justin Andrew Johnson[3], Pasquale Borrelli[4,5], Panos Panagos[6], Edna J. Molina Bacca[1,2], Kristine Karstens[1,2], Florian Humpenöder[1], Jan Philipp Dietrich[1], Sara Minoli[1], Christoph Müller[1], Hermann Lotze-Campen[1,2], Alexander Popp[1]

[1]Potsdam Institute for Climate Impact Research (PIK), Member of the Leibniz Association, PO Box 601203, 14412 Potsdam, Germany

[2]Albrecht Daniel Thaer-Institute of Agricultural and Horticultural Sciences, Humboldt University of Berlin, Germany

[3]Department of Applied Economics, University of Minnesota, 1940 Buford Ave, Saint Paul, MN 55105 USA

[4]Department of Environmental Sciences, Environmental Geosciences, University of Basel, Basel, Switzerland

[5]Department of Science, Roma Tre University, Rome, Italy

[6]European Commission, Joint Research Centre (JRC), Ispra (VA), IT-21027, Italy

*Corresponding author. E-mail: vjeetze@pik-potsdam.de

Table of Contents

1 Supplementary Table 2
2 Supplementary Figures 4
2.1 Sensitivity Analysis 20
3 Supplementary Methods 24
3.1 Model of Agricultural Production and its Impact on the Environment (MAgPIE) 24
3.1.1 Spatial Resolution and Internal Downscaling 24
3.1.2 Land Pools 27
3.1.3 Area Potentially Suitable for Cropland 28
3.1.4 Yields 29
3.1.5 Carbon 30
3.1.6 Scenario Inputs 31
3.2 Spatial Economic Allocation Landscape Simulator (SEALS) 32
3.2.1 Allocation Algorithm 32
3.2.2 Model Calibration 34
3.2.3 Calibrated Parameters 34
3.2.4 Current Limitations 36
3.3 Global Soil Erosion Modelling (GloSEM) 40
3.3.1 Revised Universal Soil Loss Equation (RUSLE) 40
3.3.2 GloSEM Implementation 40
3.3.3 Land Cover and Management Factor (C) 40
4 Supplementary References 45

1 Supplementary Table

Supplementary Table 1: Nature's contributions to people as defined in IPBES (2019) and indicators covered in this study.

Nature's contribution to people (NCP)		Indicators	Scale of assessment
Regulating NCP	1 Habitat creation and maintenance	• Extent of natural land (forest and non-forest ecosystems)<br>• Extent of (semi-)natural habitat in agricultural landscapes	Global/regional & Field-scale
	2 Pollination and dispersal of seeds and other propagules	• Pollination sufficiency (amount of pollinator habitat in foraging distance of agricultural land)	Field-scale
	3 Regulation of air quality	-	-
	4 Regulation of climate	• Carbon uptake and losses from land-use change<br>• Carbon price-induced afforestation area	Global/regional
	5 Regulation of ocean acidification	-	-
	6 Regulation of freshwater quantity, location and timing	-	-
	7 Regulation of freshwater and coastal water quality	-	-
	8 Formation, protection, and decontamination of soils and sediments	• Soil loss by water erosion	Field-scale
	9 Regulation of hazards and extreme events	-	-
	10 Regulation of detrimental organisms and biological processes	• Extent of (semi-)natural habitat in agricultural landscapes	Field-scale

Material NCP	11 Energy	• Extent of agricultural land (bioenergy production)	Global/regional
	12 Food and feed	• Extent of agricultural land (food and feed concentrates production)	Global/regional
	13 Materials and assistance	• Extent of agricultural land (material production)	Global/regional
	14 Medicinal, biochemical, and genetic resources	-	-
Non-mat. NCP	15 Learning and inspiration	-	-
	16 Physical and psychological experience	-	-
	17 Supporting identities	-	-
	18 Maintenance of options	-	-

2 Supplementary Figures

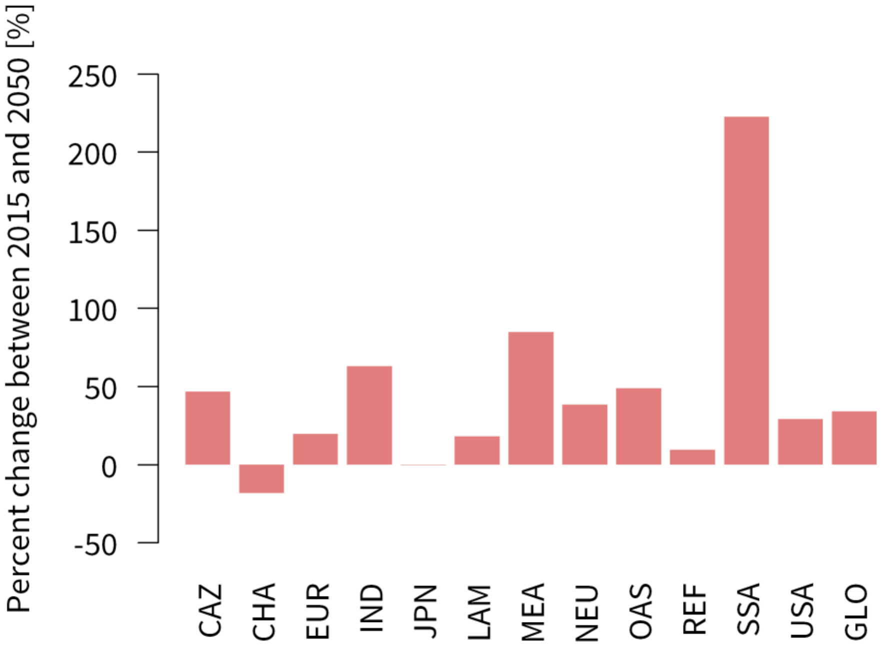

Supplementary Fig. 1: Regional demand changes for food crops reliant on biotic pollination between 2015 and 2050.

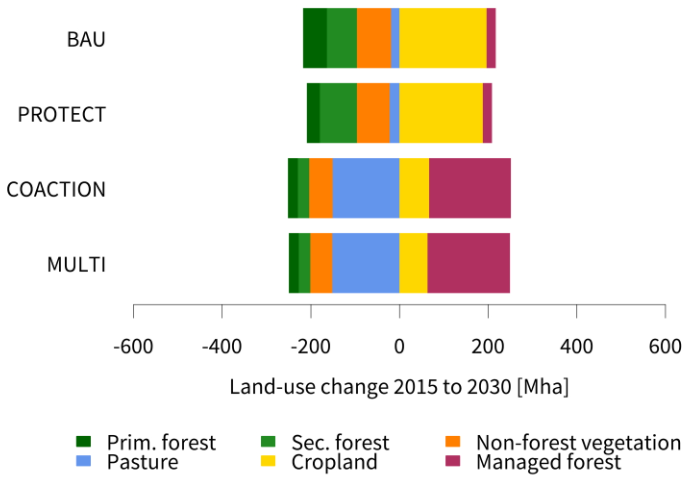

Supplementary Fig. 2: Global projections of land-use change between 2015 and 2030. For the reference year 2015, global land cover includes 1654.04 Mha of cropland (food, feed and bioenergy crops), 3202.36 Mha of pasture area, 3979.18 Mha of forest and 3929.85 Mha of non-forest vegetation. Forestry features afforestation based on NDCs and carbon price-induce afforestation, as well as plantations.

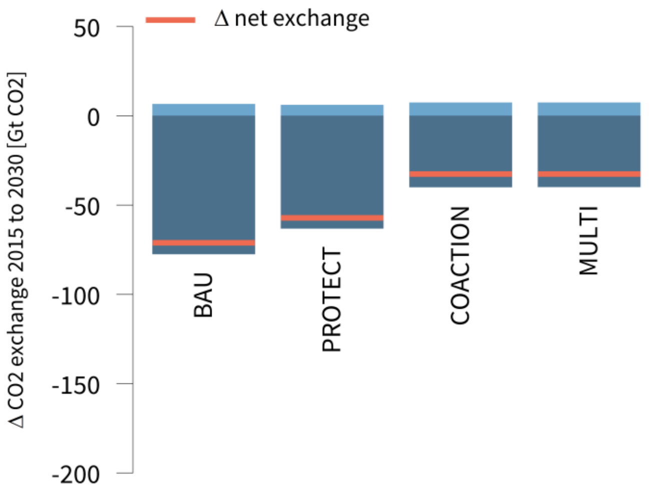

Supplementary Fig. 3: CO_2 exchange caused by land-use change between 2015 and 2030. Negative values (dark blue) indicate global carbon losses in terms of CO_2 from the conversion of pasture, forest and non-forest ecosystems, while positive values (light blue) show carbon uptake from regrowth due to land abandonment and afforestation. Orange lines depict the net CO_2 exchange between 2015 and 2030.

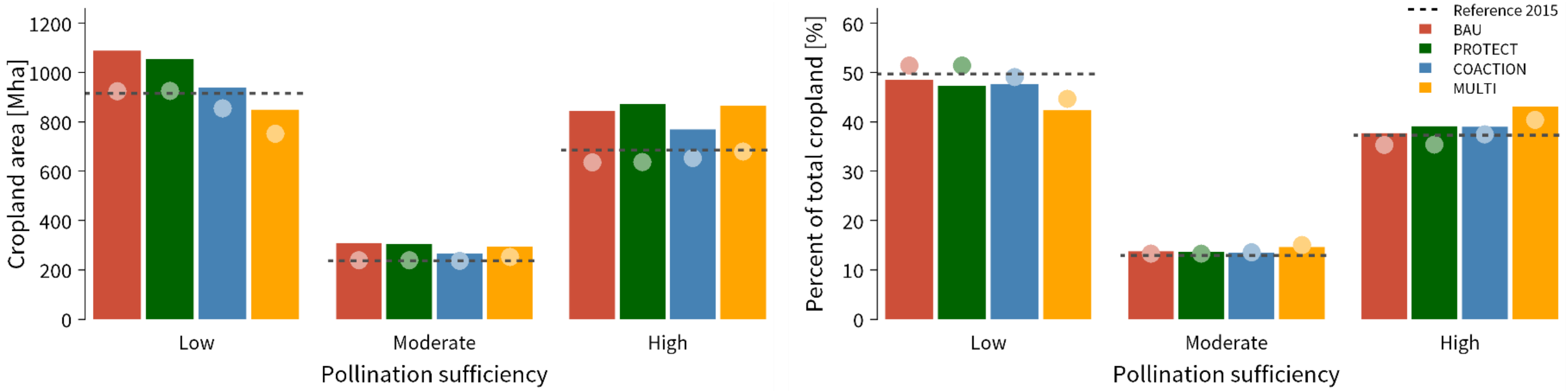

Supplementary Fig. 4: Global cropland area categorised according to pollination sufficiency values. The left panel depicts total cropland area in each pollination sufficiency class, while the right panel shows the area percentages in each class. Bars represent overall values, while shaded dots illustrate 2050 pollination sufficiency values in historic cropland areas only. Dashed lines denote global values for 2015.

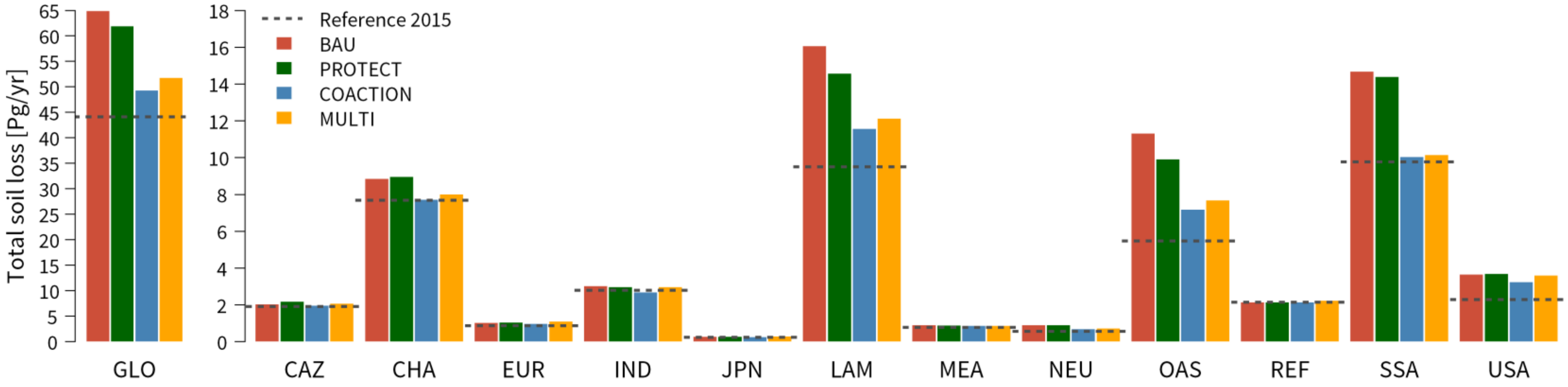

Supplementary Fig. 5: Estimated total global and regional soil loss by water erosion. Dashed lines show total soil loss in 2015. GLO: Global; CAZ: Canada, Australia and New Zealand; CHA: China; EUR: European Union; IND: India; JPN: Japan; LAM: Latin America; MEA: Middle East and north Africa; NEU: non-EU member states; OAS: other Asia; REF: reforming countries; SSA: Sub-Saharan Africa; USA: United States.

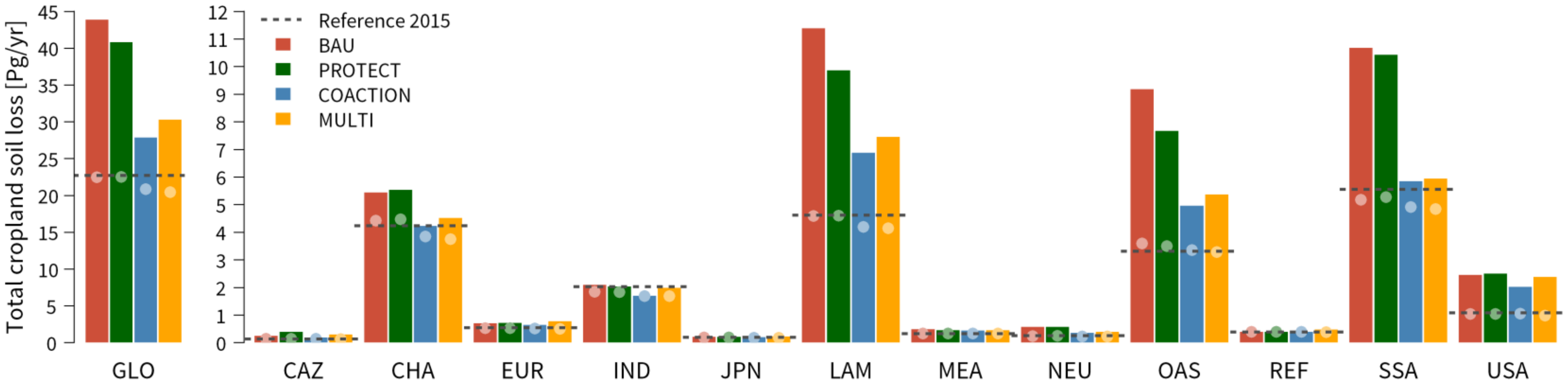

Supplementary Fig. 6: Estimated total global and regional soil loss by water erosion in cropland areas. Bars represent overall changes, while shaded dots illustrate 2050 rates of soil loss in historic cropland areas only. Dashed line show cropland soil loss in 2015. GLO: Global; CAZ: Canada, Australia and New Zealand; CHA: China; EUR: European Union; IND: India; JPN: Japan; LAM: Latin America; MEA: Middle East and north Africa; NEU: non-EU member states; OAS: other Asia; REF: reforming countries; SSA: Sub-Saharan Africa; USA: United States.

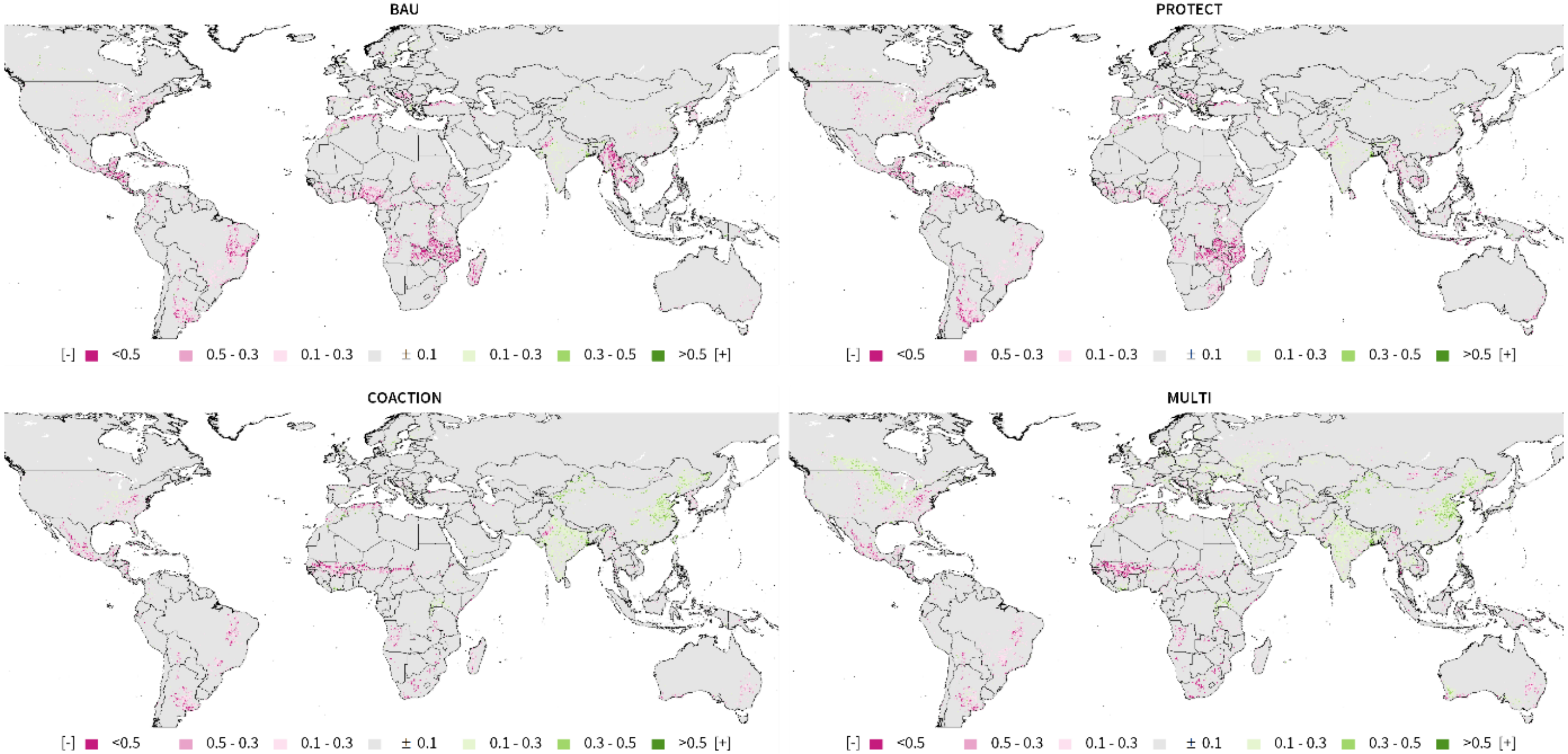

Supplementary Fig. 7: Pollination sufficiency changes between 2015 and 2050 across modelled scenarios.

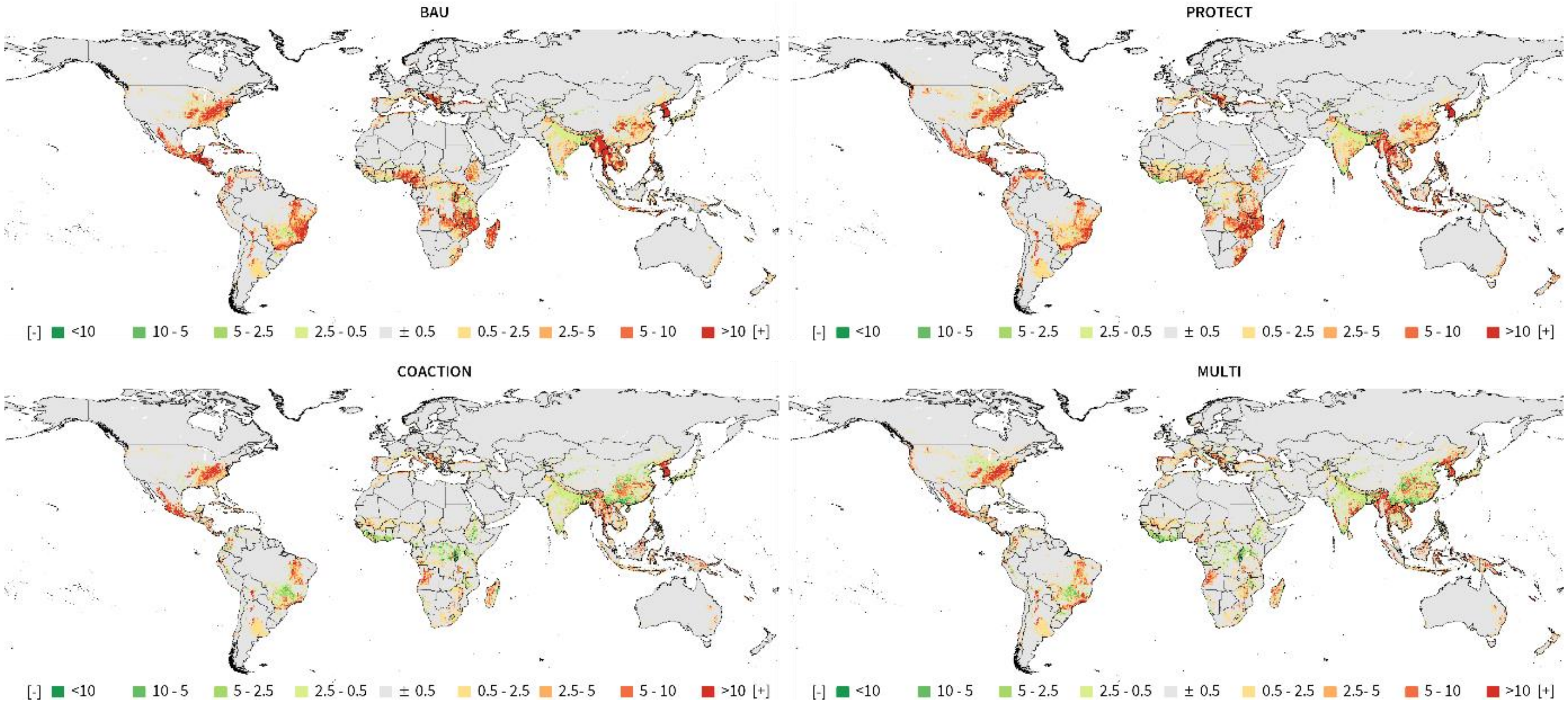

Supplementary Fig. 8: Changes in soil loss by water erosion between 2015 and 2050 for all modelled scenarios.

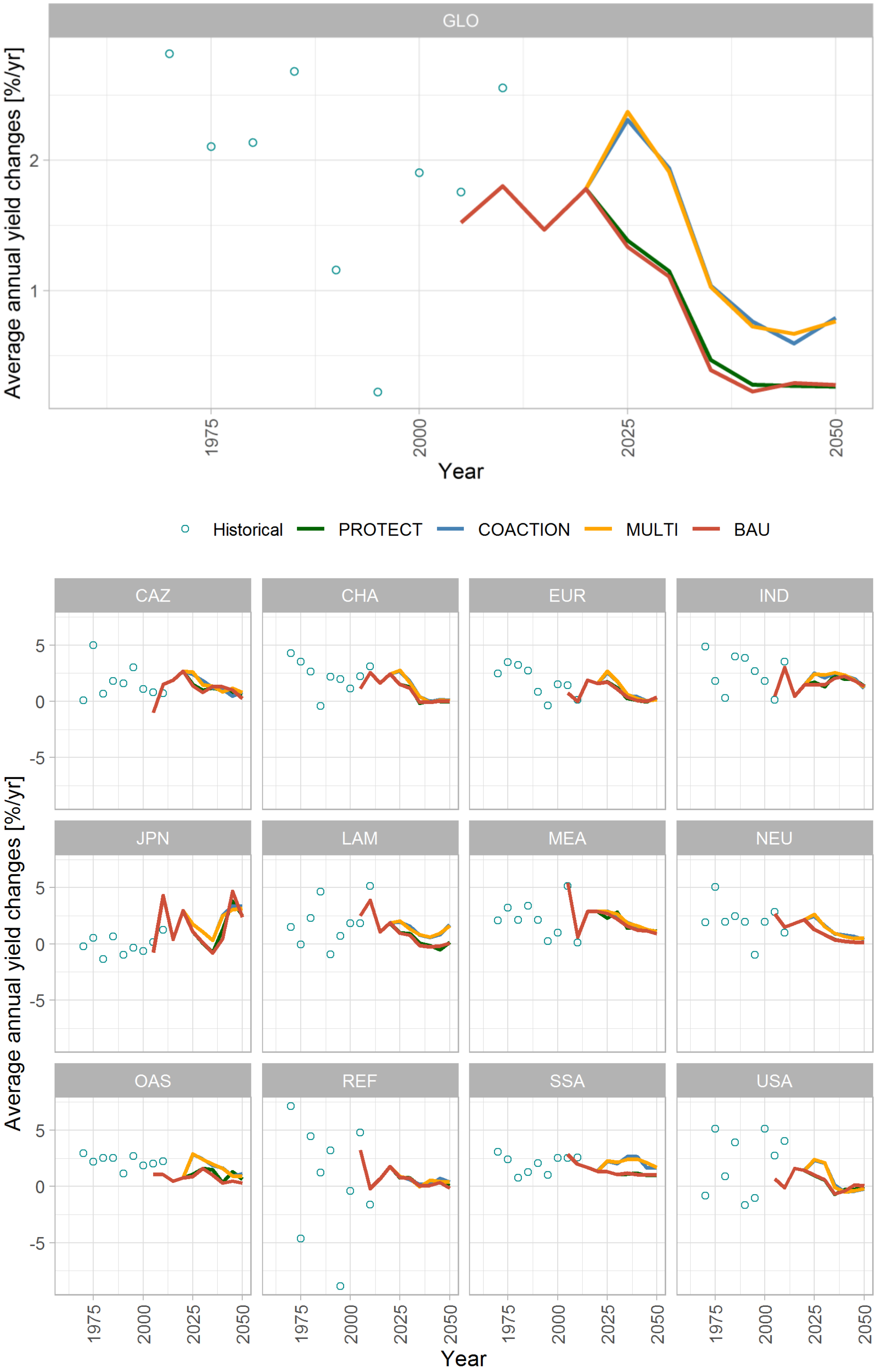

Supplementary Fig. 9: Global (top) and regional (bottom) time-series of projected average annual crop yields changes, compared to historical data from FAOSTAT[17].

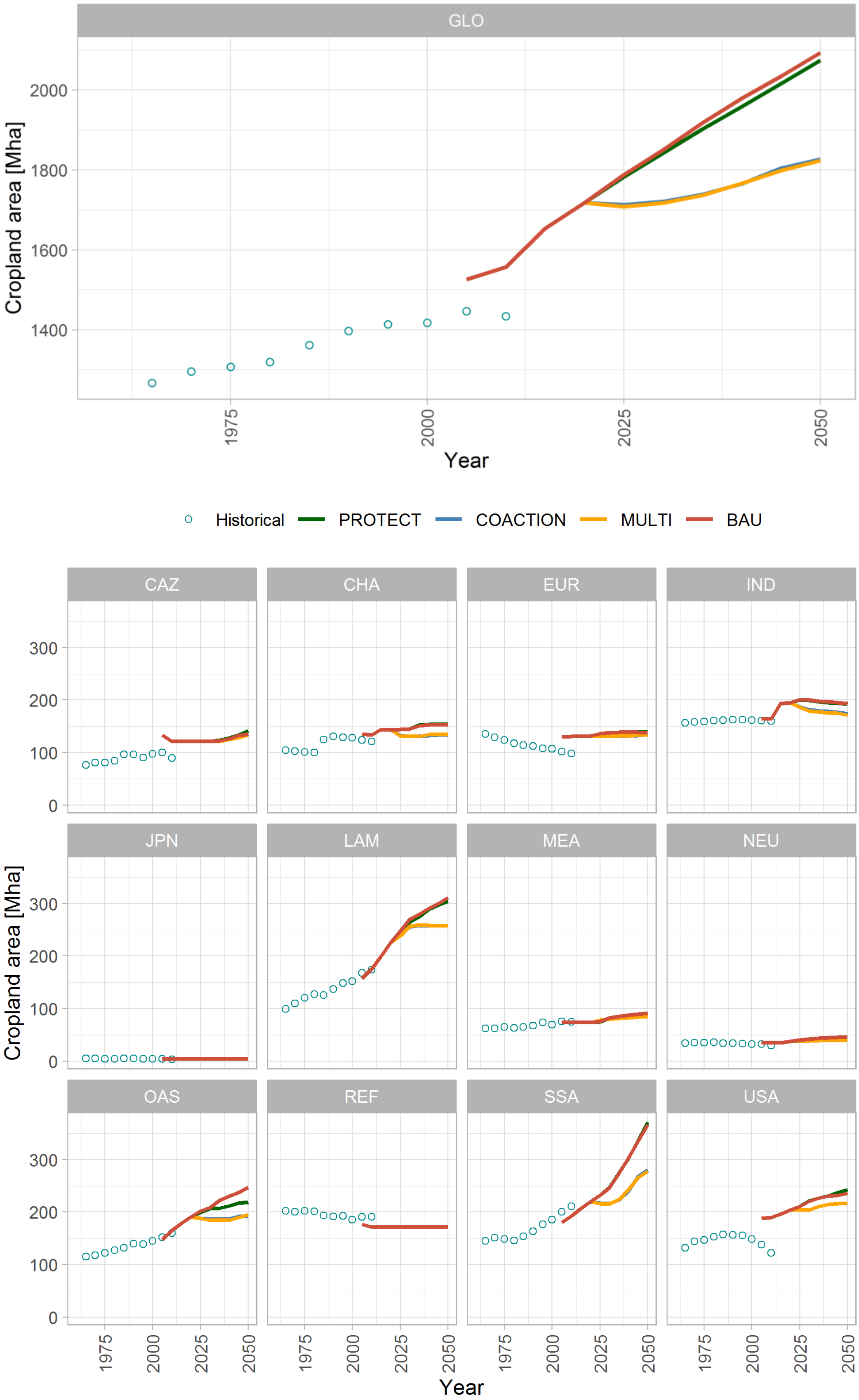

Supplementary Fig. 10: Global (top) and regional (bottom) time-series of projected cropland area, compared to historical data derived from FAOSTAT[17].

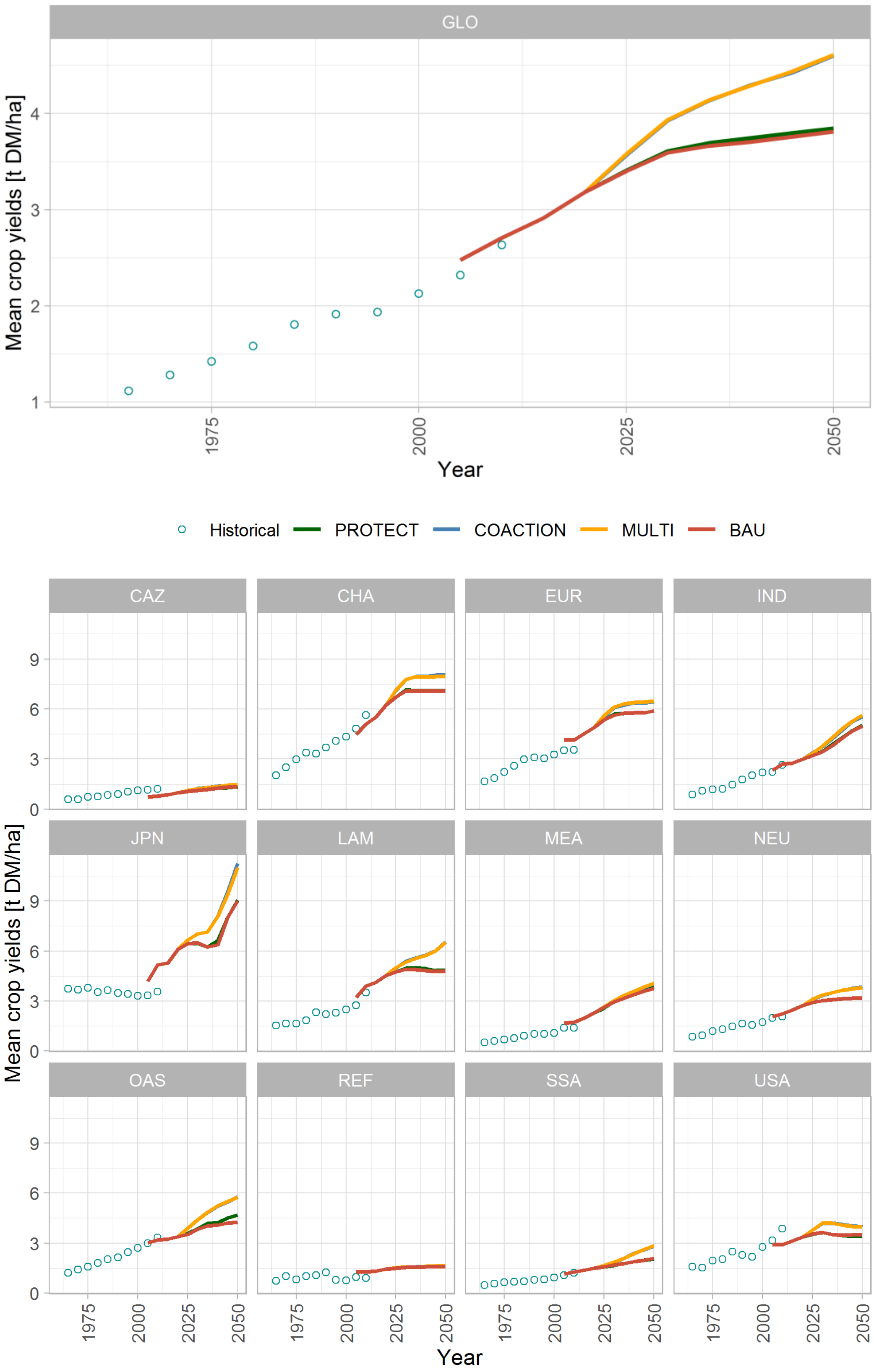

Supplementary Fig. 11: Global (top) and regional (bottom) time-series of projected mean crop yields, compared to historical data from FAOSTAT [17].

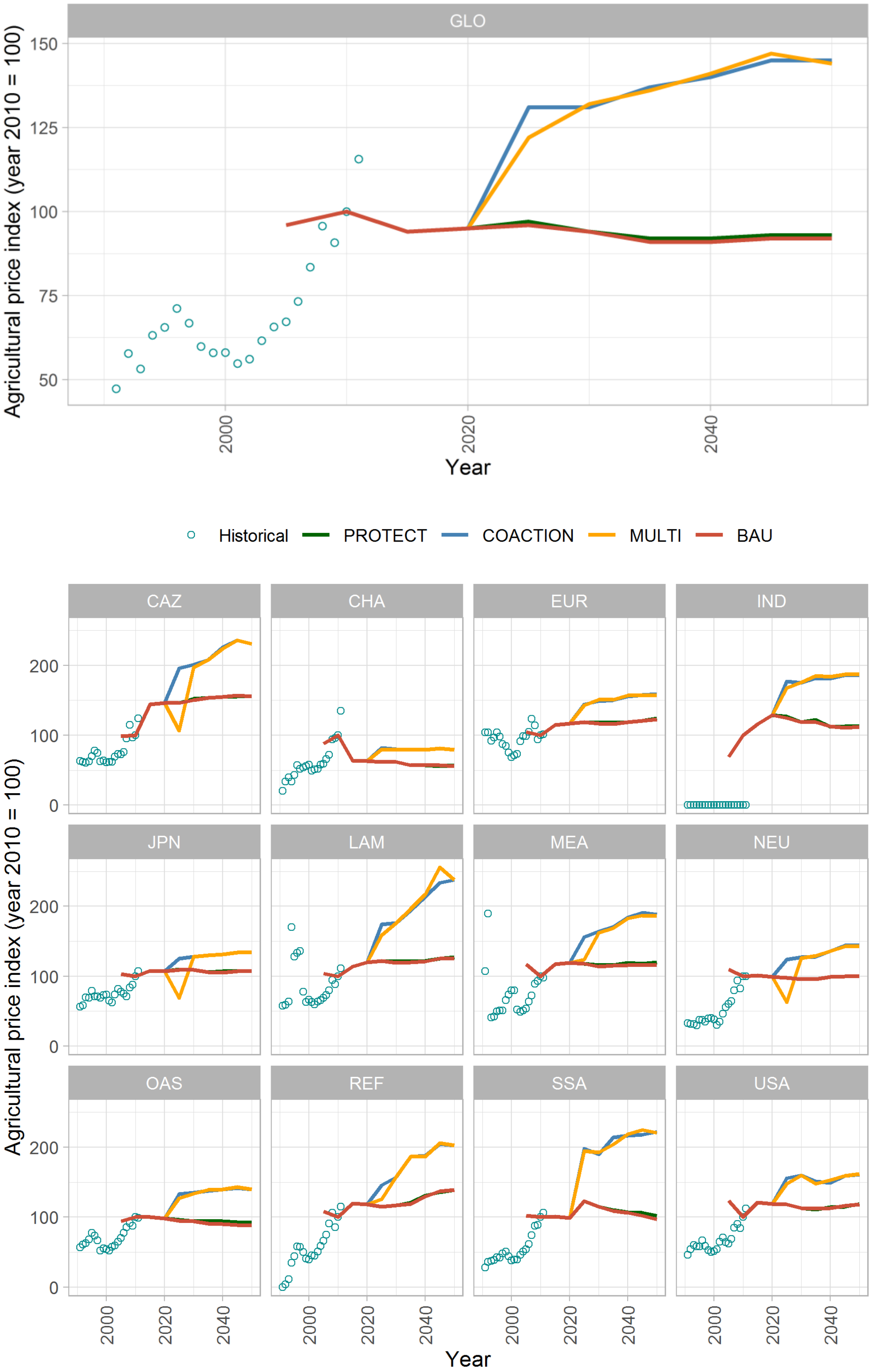

Supplementary Fig. 12: Global (top) and regional (bottom) time-series of the projected agricultural price index, compared to historical data derived from FAOSTAT[17].

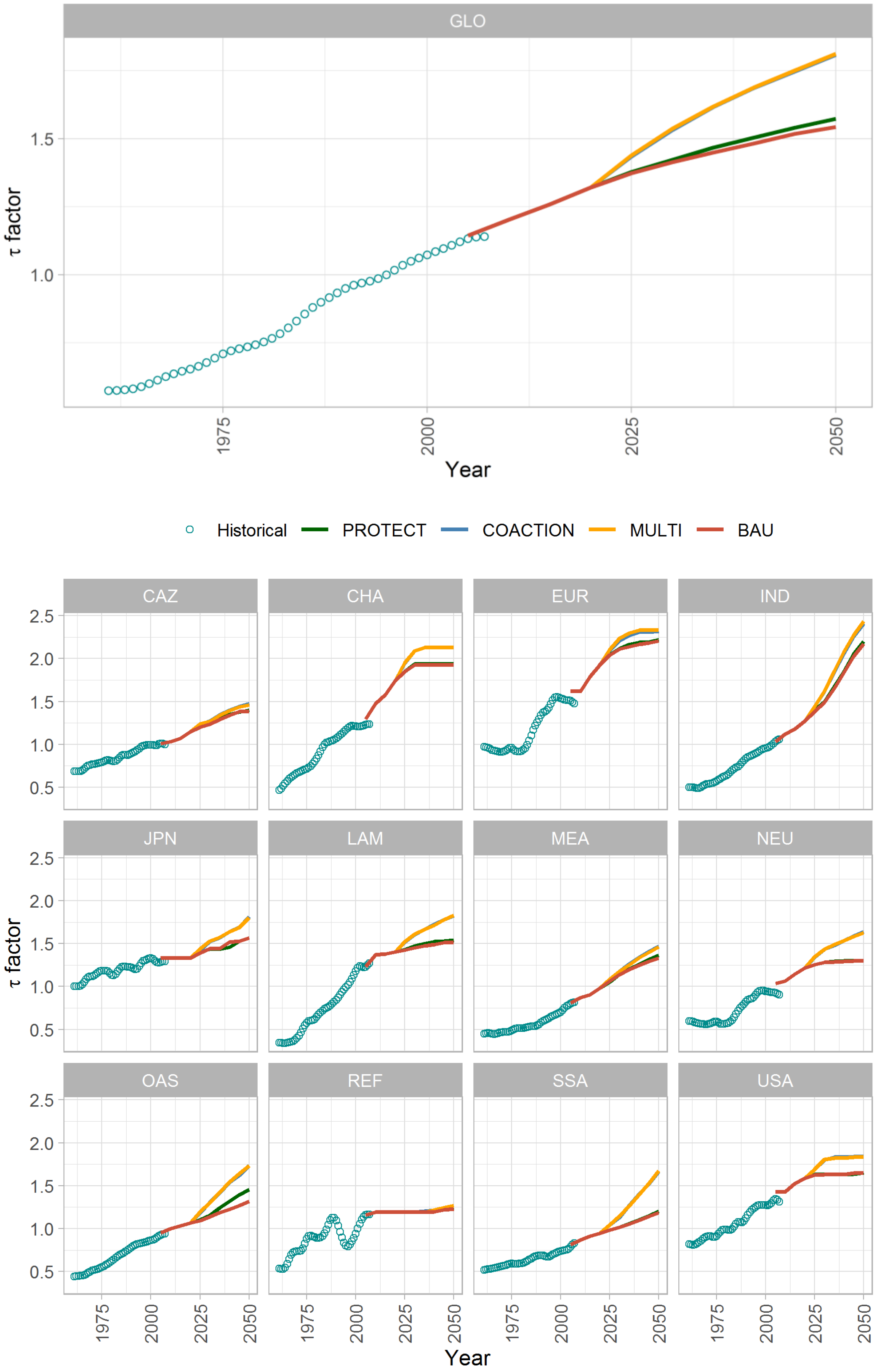

Supplementary Fig. 13: Global (top) and regional (bottom) time-series of the projected land-use intensity indicator τ, compared to historical data from Dietrich et al.[1]. The τ-factor measures technological change (TC) based on the effectiveness of R&D investments on yield changes. Investments into TC induce higher yields, but also increase the intensity of cropland use. This in turn raises the costs for further yield increases.

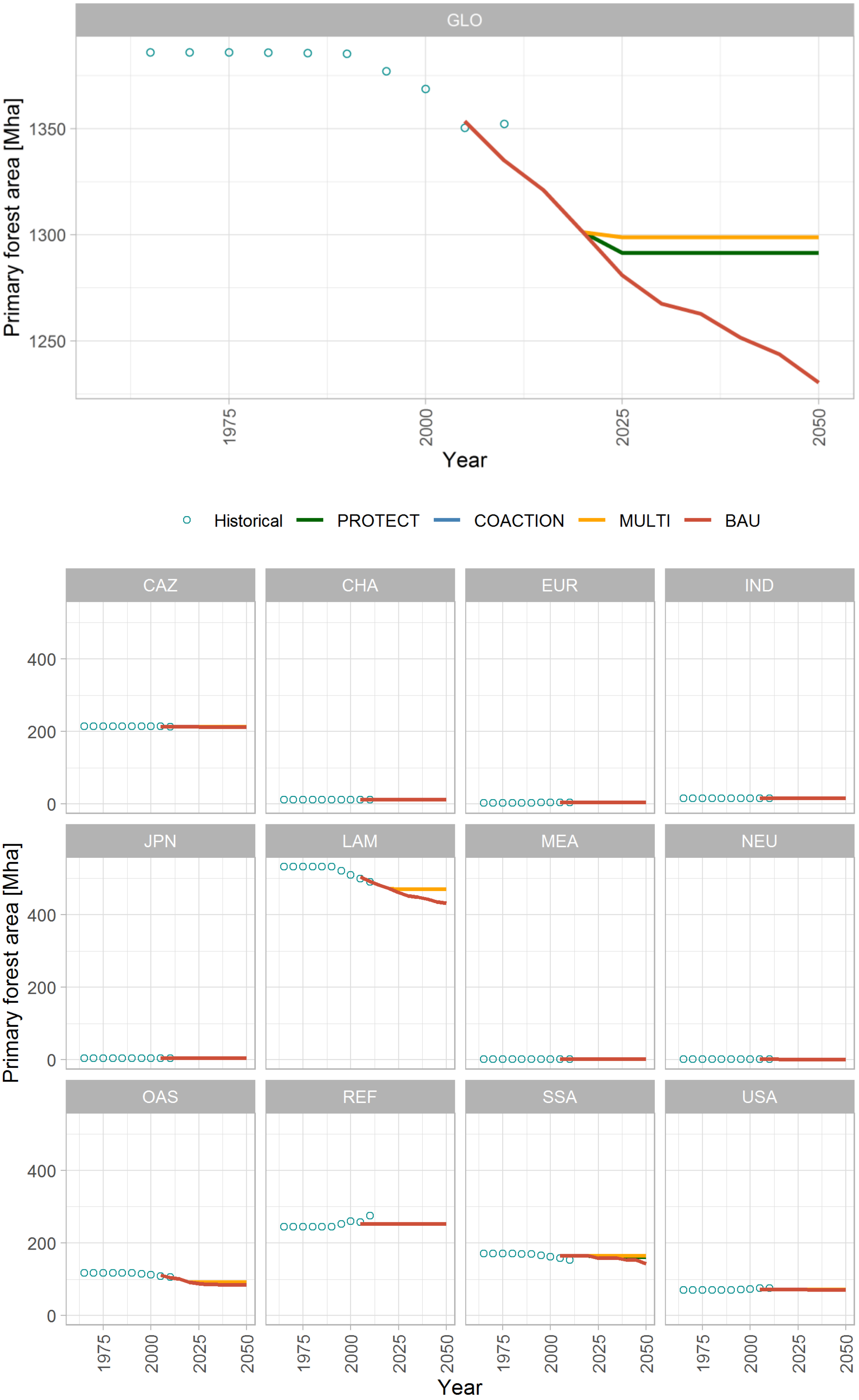

Supplementary Fig. 14: Global (top) and regional (bottom) time-series of projected primary forest cover, compared to historical data derived from Hurtt et al.[9] and adjusted with data from MacDicken[12].

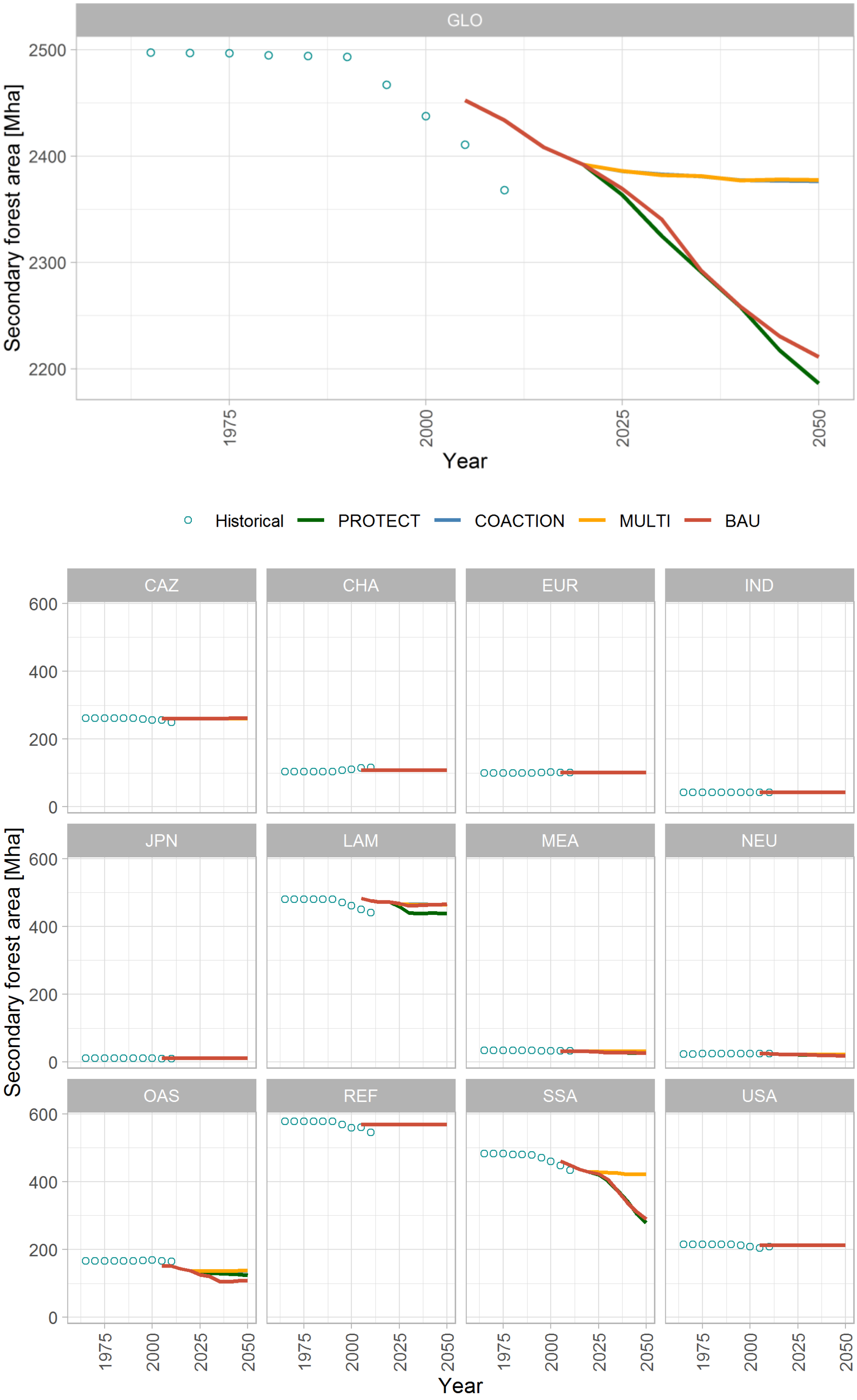

Supplementary Fig. 15: Global (top) and regional (bottom) time-series of projected secondary forest cover, compared to historical data derived from Hurtt et al.[9] and adjusted with data from MacDicken[12].

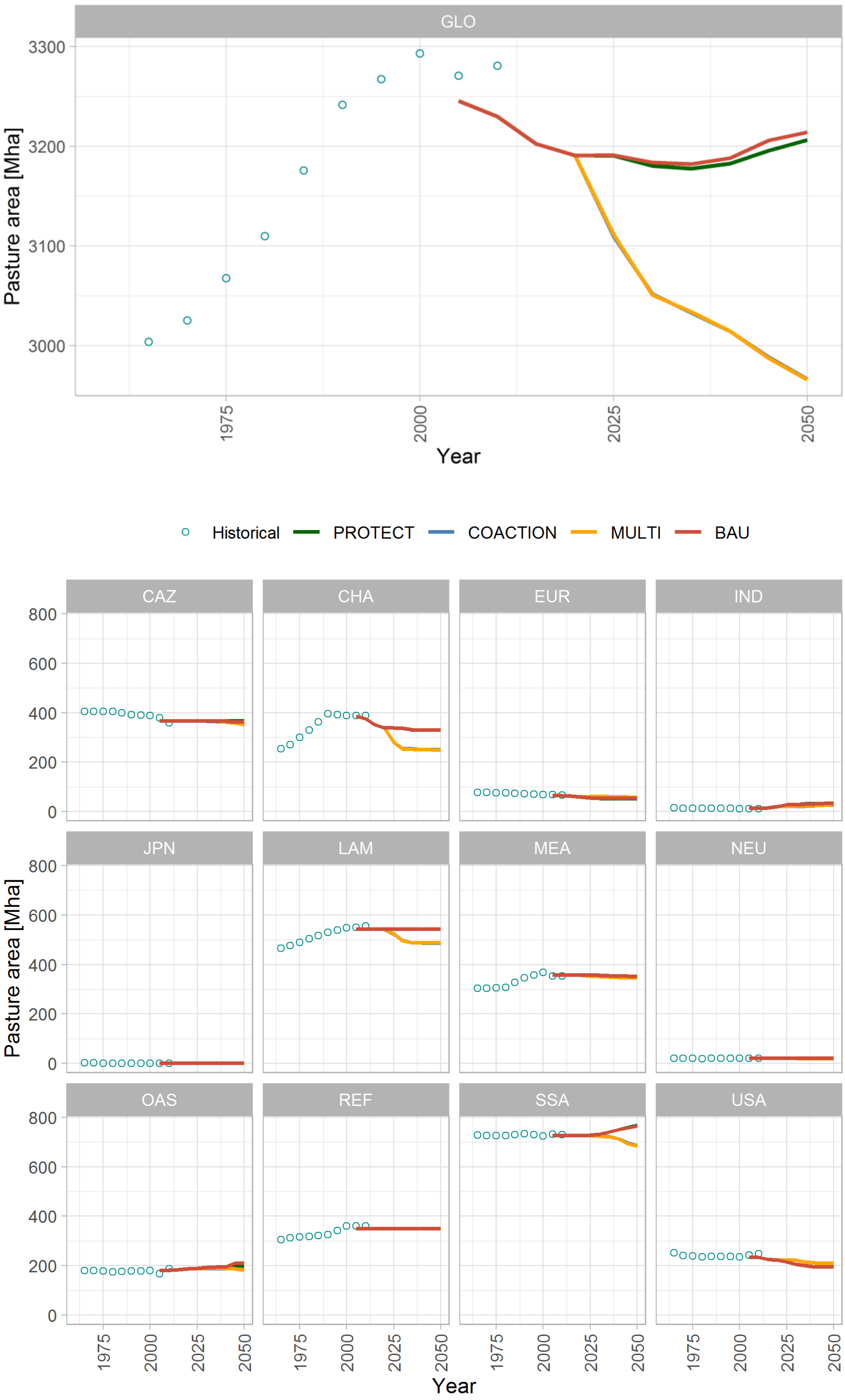

Supplementary Fig. 16: Global (top) and regional (bottom) time-series of projected pasture land, compared to historical data from Hurtt et al.[9].

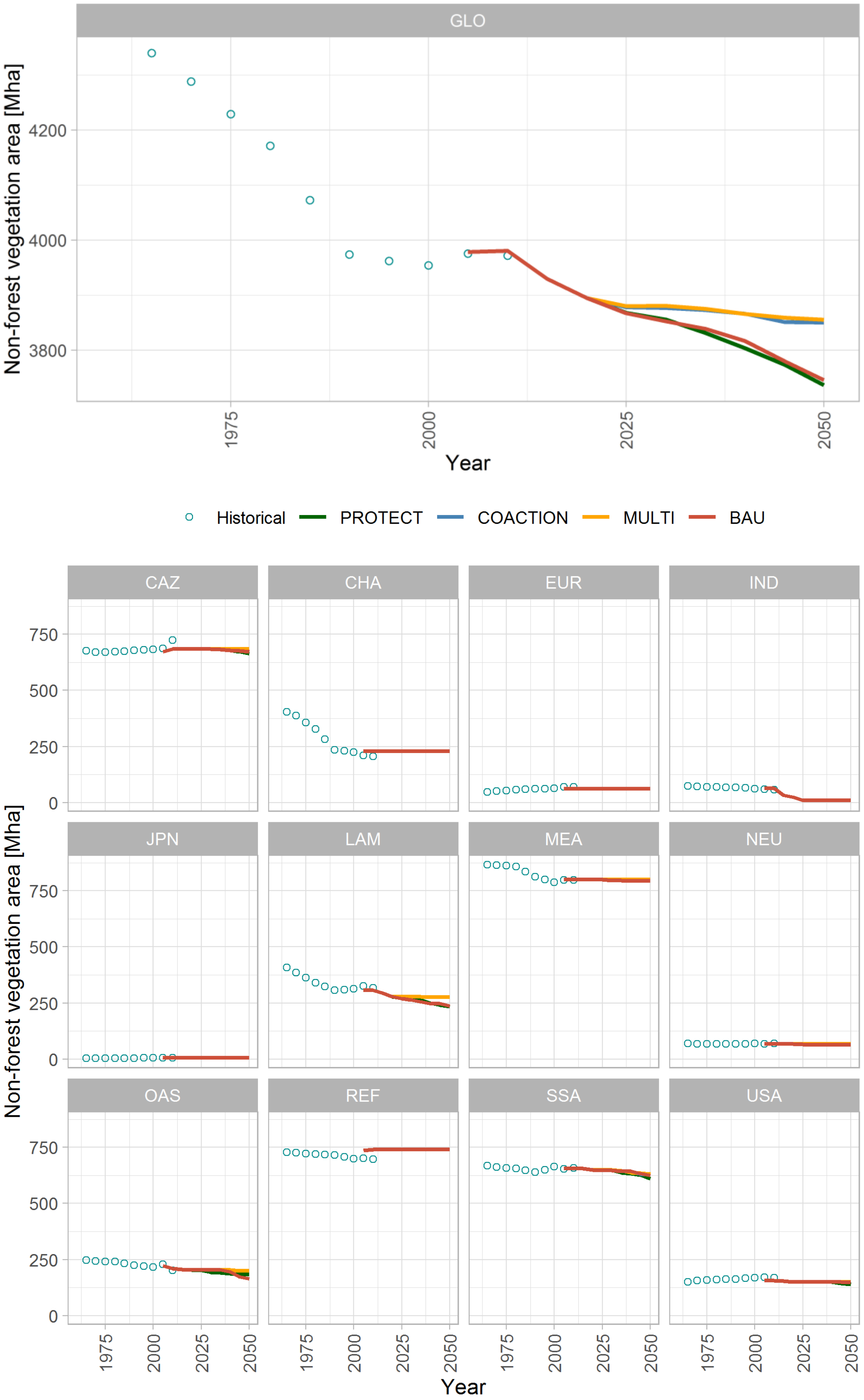

Supplementary Fig. 17: Global (top) and regional (bottom) time-series of projected non-forest vegetation cover, compared to historical data from Hurtt et al.[9].

Supplementary Table 2: Global and regional soil loss estimates for the reference year 2015 and projections for the different scenarios.

	Cropland soil loss [Pg/yr]					Total soil loss [Pg/yr]				
	Ref. 2015	BAU	PROTECT	COACTION	MULTI	Ref. 2015	BAU	PROTECT	COACTION	MULTI
GLO	22.7	43.9	40.8	27.8	30.3	44.1	64.8	61.9	49.3	51.7
CAZ	0.1	0.3	0.4	0.2	0.3	1.9	2.0	2.2	2.0	2.1
CHA	4.2	5.4	5.5	4.2	4.5	7.7	8.8	8.9	7.7	8.0
EUR	0.5	0.7	0.7	0.6	0.8	0.9	1.0	1.0	1.0	1.1
IND	2.0	2.1	2.0	1.7	2.0	2.8	3.0	3.0	2.7	3.0
JPN	0.2	0.2	0.2	0.2	0.2	0.2	0.3	0.3	0.2	0.3
LAM	4.6	11.4	9.9	6.9	7.4	9.5	16.1	14.6	11.6	12.1
MEA	0.3	0.5	0.4	0.4	0.4	0.8	0.9	0.9	0.9	0.9
NEU	0.3	0.6	0.6	0.4	0.4	0.6	0.9	0.9	0.7	0.7
OAS	3.3	9.2	7.7	5.0	5.4	5.5	11.3	9.9	7.2	7.7
REF	0.4	0.4	0.4	0.4	0.5	2.1	2.1	2.1	2.1	2.2
SSA	5.6	10.7	10.4	5.8	5.9	9.8	14.7	14.4	10.0	10.1
USA	1.1	2.5	2.5	2.0	2.4	2.3	3.6	3.7	3.2	3.6

2.1 Sensitivity Analysis

With this sensitivity analysis we address the uncertainty of important exogenous model parameters in the context of our analysis. A key aspect of our analysis are changing land-use dynamics associated with growing demand for land-based products and ambitious targets for biodiversity and climate protection. In this context, the competition for land depends, in particular, on varying assumptions with regard to the implementation of yield-increasing technologies and trade liberalisation, which facilitates land allocation. Land-use dynamics in this study also depend on different levels of ambition regarding land policy interventions, specifically with regard to carbon prices and landscape conservation targets. We therefore varied the default model assumptions of these critical parameters within MAgPIE, in order to test how the changed parametrisation drives our simulation outcomes (Supplementary Figs. 18 & 19). Supplementary Table 3 provides an overview of the parameter changes applied in MAgPIE during the sensitivity analysis.

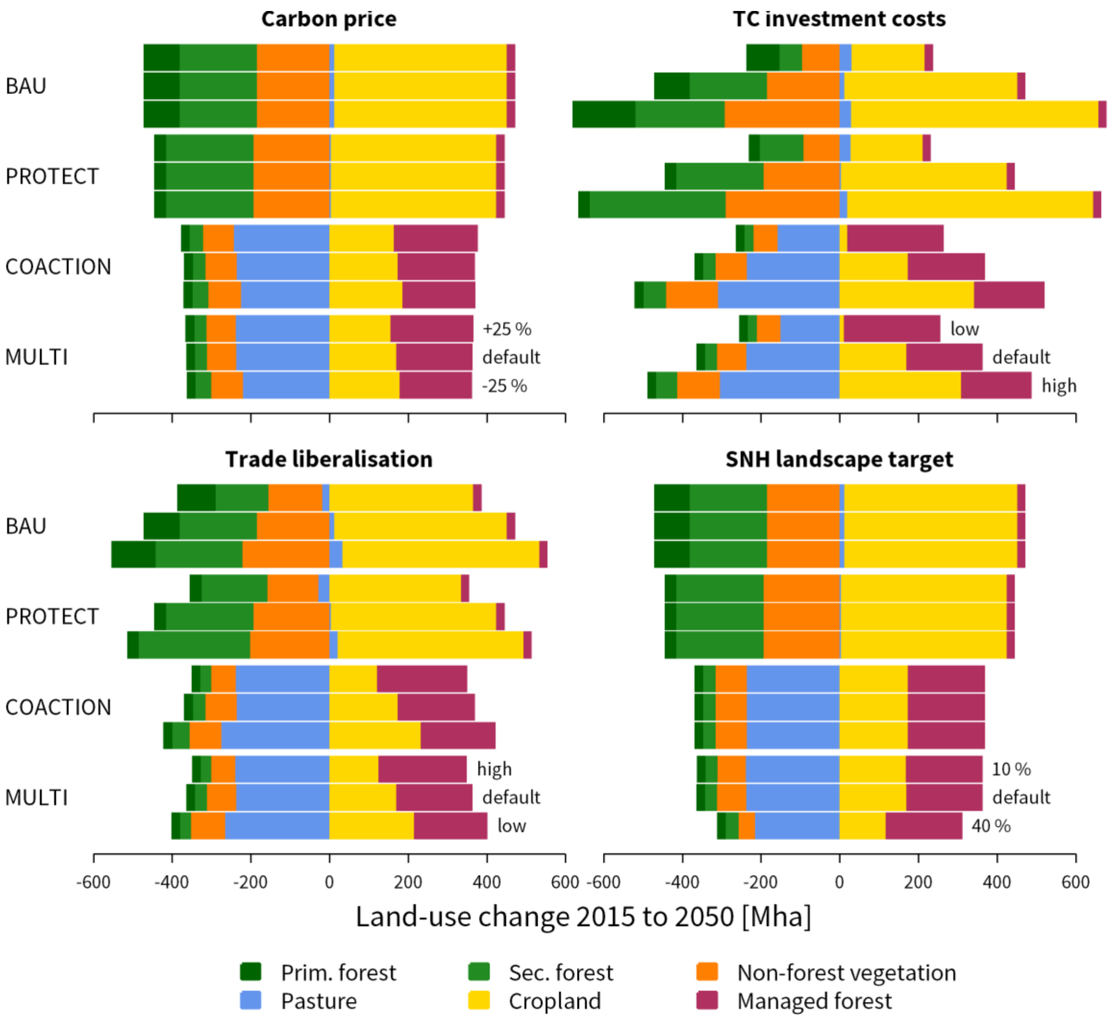

Supplementary Fig. 18: Sensitivity analysis of projected global land-use change between 2015 and 2050. The different parameter settings are described in Supplementary Tab. 3.

Supplementary Table 3: List of exogenous model parameters varied for the sensitivity analysis.

Exogenous model parameter	Parameter setting			Parameter description
	Lower	Default	Higher	
TC investment costs	$\mathrm{IY}(\tau_i) = 1500\tau_i^{1.5}$	$\mathrm{IY}(\tau_i) = 1900\tau_i^{2.4}$	$\mathrm{IY}(\tau_i) = 2300\tau_i^{3.3}$	The costs for increasing agricultural productivity (technological change, TC) are described via the investment-yield relationship IY (USD ha^{-1})[1]. The variation of this relation is based on the standard error of the prefactor and the exponent as given in Dietrich et al.[1]. τ_i describes the agricultural land use intensity in region i.
Trade liberalisation	*2030:* Livestock/secondary products 10 %, crops 20 % *2050:* Livestock/secondary products 5 %, crops 10 %	*After 2030:* Livestock/secondary products 10 %, Crops 20%	*2030:* Livestock/secondary products 10 %, crops 20 % *2050:* Livestock/secondary products 20 %, crops 30 %	Trade barrier reduction assumed over time[2,3]. The shares given denote how much of total production is allocated according to comparative advantage criteria only (deviating from historic trade patterns) by the given year.
Carbon price	- 25 %	*2025*: 109.8 USD *2050*: 371.8 USD	+ 25 %	Global universal carbon tax consistent with the Paris Agreement as derived with the coupled REMIND-MAgPIE modelling framework[4]
SNH landscape target	10 %	20 %	40 %	Global target for conserving (semi-)natural habitat (SNH) in farmed landscapes. The target is expressed in terms of the total potential available cropland (see Methods)

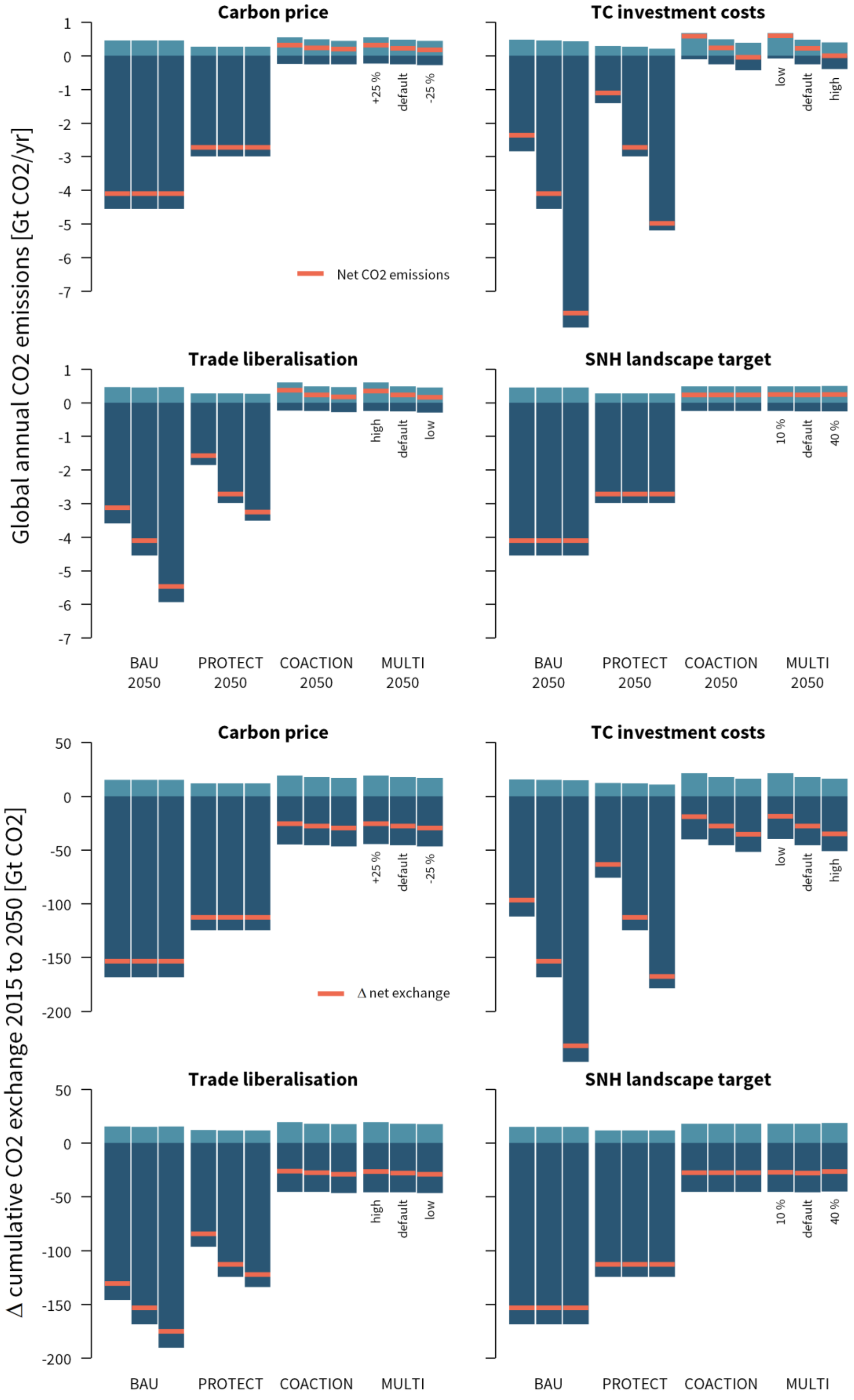

Supplementary Fig. 19: Sensitivity analysis of projected CO_2 emissions from land-use change. Global annual CO2 emissions (top) and cumulative CO2 exchange caused by land-use change between 2015 and 2050 (bottom). The parameter settings of the sensitivity analysis are described in Supplementary Tab. 3.

In addition, we also tested the model sensitivity to our definition of pollinator habitat, which in our default set-up includes any forest, non-forest and grassland vegetation cover in farmed landscapes (see Tab. 1 & Methods in the main article). To assess the sensitivity of our pollination sufficiency estimates to this definition of pollinator habitat, we reran our analysis after excluding grassland areas from our definition of pollinator habitat. The results are shown in Supplementary Fig. 20.

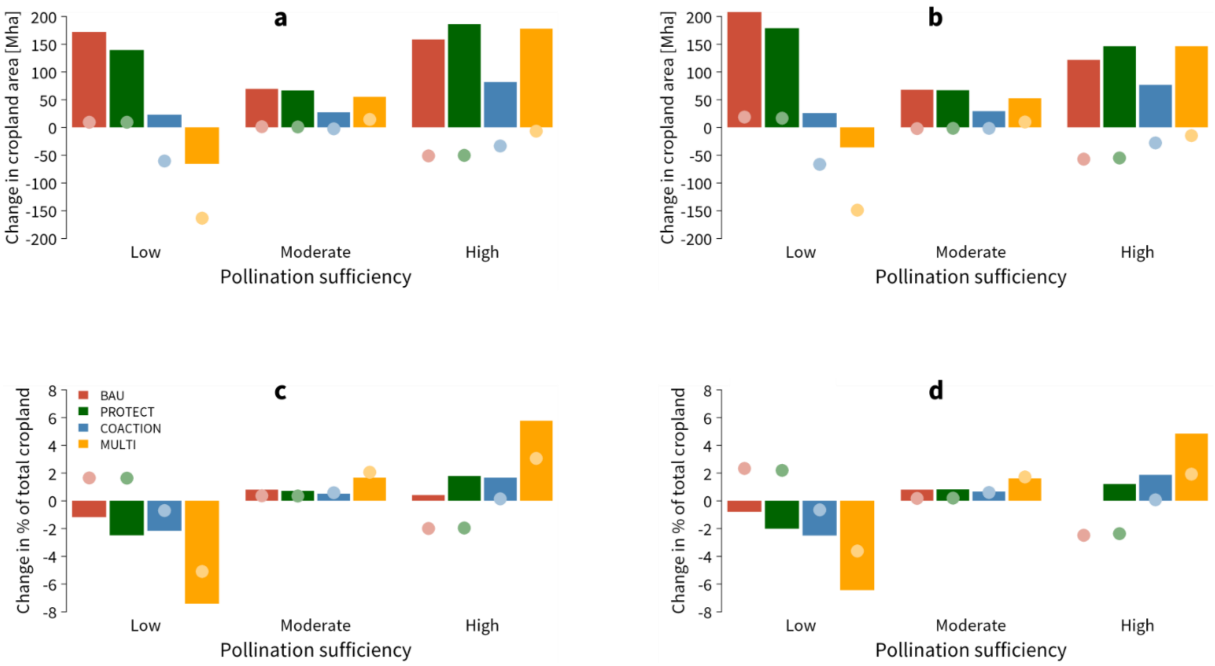

Supplementary Fig. 20: Sensitivity analysis of pollination sufficiency estimates to variation in the pollinator habitat definition. (a) and **(c)** show estimated changes in pollination sufficiency based on our default definition of pollinator habitat, which includes forest, non-forest and grassland vegetation (see also Fig. 4 in the main text). **(b)** and **(d)** in comparison show changes in pollination sufficiency after excluding grassland from our definition of pollinator habitat.

3 Supplementary Methods

3.1 Model of Agricultural Production and its Impact on the Environment (MAgPIE)

3.1.1 Spatial Resolution and Internal Downscaling

The MAgPIE model currently operates at two built-in spatial levels, including a coarse level of twelve model regions (Supplementary Table 4) with similar socioeconomic characteristics and a finer resolution of spatial clusters with similar biophysical properties and suitability for agricultural production (e.g. crop yield potential, irrigation water requirements and travel time to urban markets from Nelson[5]) at the subregional level (Supplementary Fig. 21).

The world regions are used to define socioeconomic constraints, input parameters and variables such as food demand, trade patterns or interest rates. Thereby small economies are grouped together, while larger economies, such as USA, China or India, are resolved individually. Supplementary Table 5 provides an overview of the countries grouped in each MAgPIE region.

Processes like land competition, agricultural production or irrigation are modelled at a finer spatial scale (spatial clusters) that is based on biophysical information at the 0.5 degree grid cell level. This includes crop and pasture yield potentials, terrestrial carbon densities (vegetation, litter and soil) and water availability, which are derived from the crop, vegetation and hydrology model LPJmL[6,7], but also other information, such as land potentially suitable for cropping activities[8] or the land pools in the initial year 1995[9]. All inputs at 0.5 degree spatial resolution are aggregated to 200 clusters (default setup) due to computational constraints. The aggregation is based on a k-means clustering algorithm that considers the similarity of biophysical and other constraints, such as travel time to urban markets[10].

After the optimisation, modelled outcomes at the cluster level are then downscaled back to 0.5 degree using the function 'interpolateAvlCroplandWeighted' that is part of and documented in the in-house-developed R package 'luscale'[11].

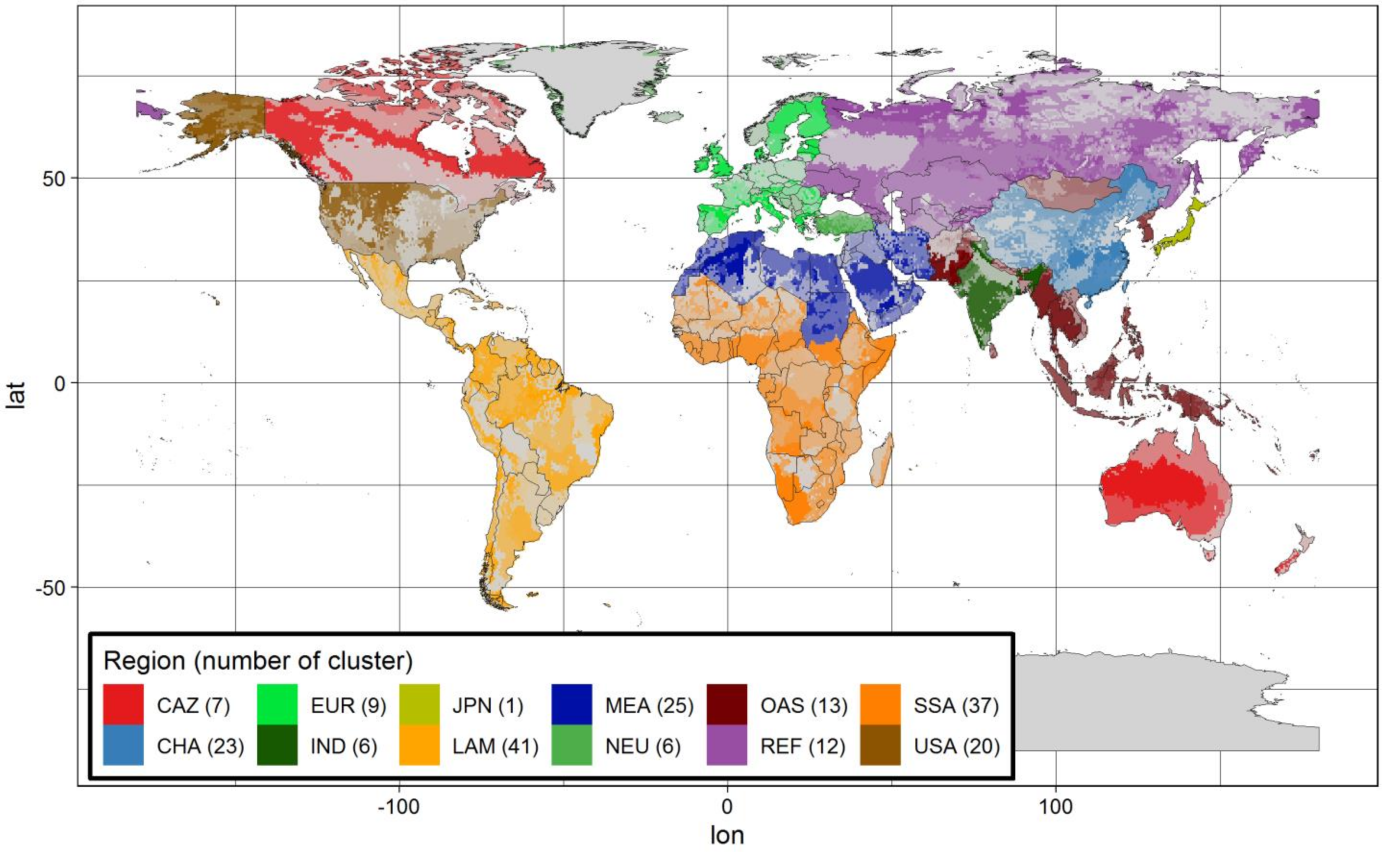

Supplementary Fig. 21: Map of MAgPIE world regions and spatial clusters (number in brackets) belonging to each MAgPIE region. This set-up includes 12 equally treated world regions and 200 spatial simulation clusters.

Supplementary Table 4: List of MAgPIE regions used in this study.

Abbreviation	MAgPIE region
CAZ	Canada, Australia and New Zealand
CHA	China
EUR	European Union
IND	India
JPN	Japan
LAM	Latin America
MEA	Middle-East and North Africa
NEU	Non-EU member states
OAS	Other Asia
REF	Reforming countries
SSA	Sub-Saharan Africa
USA	United States

Supplementary Table 5: ISO3 codes of countries grouped to standard MAgPIE regions.

MAgPIE region	ISO3 country codes
CAZ	AUS, CAN, HMD, NZL, SPM
CHA	CHN, HKG, MAC, TWN
EUR	ALA, AUT, BEL, BGR, CYP, CZE, DEU, DNK, ESP, EST, FIN, FRA, FRO, GBR, GGY, GIB, GRC, HRV, HUN, IMN, IRL, ITA, JEY, LTU, LUX, LVA, MLT, NLD, POL, PRT, ROU, SVK, SVN, SWE
IND	IND
JPN	JPN
LAM	ABW, AIA, ARG, ATA, ATG, BES, BHS, BLM, BLZ, BMU, BOL, BRA, BRB, BVT, CHL, COL, CRI, CUB, CUW, CYM, DMA, DOM, ECU, FLK, GLP, GRD, GTM, GUF, GUY, HND, HTI, JAM, KNA, LCA, MAF, MEX, MSR, MTQ, NIC, PAN, PER, PRI, PRY, SGS, SLV, SUR, SXM, TCA, TTO, URY, VCT, VEN, VGB, VIR
MEA	ARE, BHR, DZA, EGY, ESH, IRN, IRQ, ISR, JOR, KWT, LBN, LBY, MAR, OMN, PSE, QAT, SAU, SDN, SYR, TUN, YEM
NEU	ALB, AND, BIH, CHE, GRL, ISL, LIE, MCO, MKD, MNE, NOR, SJM, SMR, SRB, TUR, VAT
OAS	AFG, ASM, ATF, BGD, BRN, BTN, CCK, COK, CXR, FJI, FSM, GUM, IDN, IOT, KHM, KIR, KOR, LAO, LKA, MDV, MHL, MMR, MNG, MNP, MYS, NCL, NFK, NIU, NPL, NRU, PAK, PCN, PHL, PLW, PNG, PRK, PYF, SGP, SLB, THA, TKL, TLS, TON, TUV, UMI, VNM, VUT, WLF, WSM
REF	ARM, AZE, BLR, GEO, KAZ, KGZ, MDA, RUS, TJK, TKM, UKR, UZB
SSA	AGO, BDI, BEN, BFA, BWA, CAF, CIV, CMR, COD, COG, COM, CPV, DJI, ERI, ETH, GAB, GHA, GIN, GMB, GNB, GNQ, KEN, LBR, LSO, MDG, MLI, MOZ, MRT, MUS, MWI, MYT, NAM, NER, NGA, REU, RWA, SEN, SHN, SLE, SOM, SSD, STP, SWZ, SYC, TCD, TGO, TZA, UGA, ZAF, ZMB, ZWE
USA	USA

3.1.2 Land Pools

Competition between land uses in MAgPIE is based on cost-effectiveness of land-use related activities, such as food, livestock and bioenergy production or afforestation at the cluster level. Land-use types used in MAgPIE include cropland, pasture, primary forest, secondary forest, forestry, other land (terrestrial non-forest ecosystems) and settlements. The spatial distribution of the different land pools is derived from the Land Use Harmonization (LUH2v2) data set[9]. The spatial distribution of forest and non-forest ecosystems, however, is harmonised with information from the Forest Resources Assessment Report[12] (FRA) by spatial relocation. Changes in cropland (rainfed and irrigated), pasture, forest and other land areas over time are endogenously determined during optimisation, while settlement areas are assumed to be constant over time. We also use the dynamic implementation of the forestry sector [13] that features afforestation for carbon dioxide removal (CDR). Afforestation can either be prescribed exogenously, e.g. based on country reports on national policies implemented (NPI) or nationally determined contributions to the Paris agreement (NDC), or modelled endogenously depending on cost incentives for CDR. Vegetation (re-)growth (forest, forestry and non-forest) follows S-shaped growth curves with parameters taken from Braakhekke et al.[14]. Spatially explicit land conservation schemes prevent expansion of cropland, pasture and forestry into natural vegetation (primary and secondary forest as well as non-forest vegetation) in areas covered by predefined conservation templates (see Methods section in the main article and Supplementary Fig. 23 for more information about the conservation templates applied in this study).

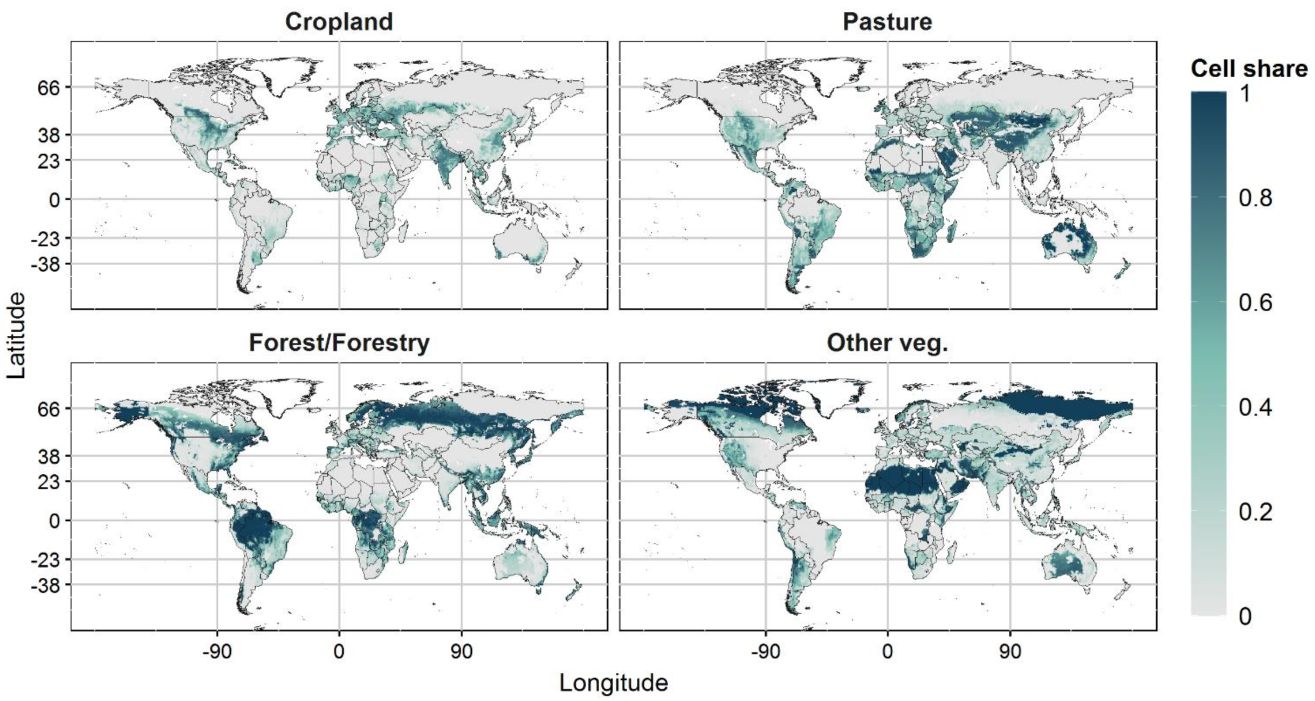

Supplementary Fig. 22: Spatially explicit land use as derived from the LUH2v2 data set[9] and used for the initialization of MAgPIE. Forest areas were adjusted with data from MacDicken[12].

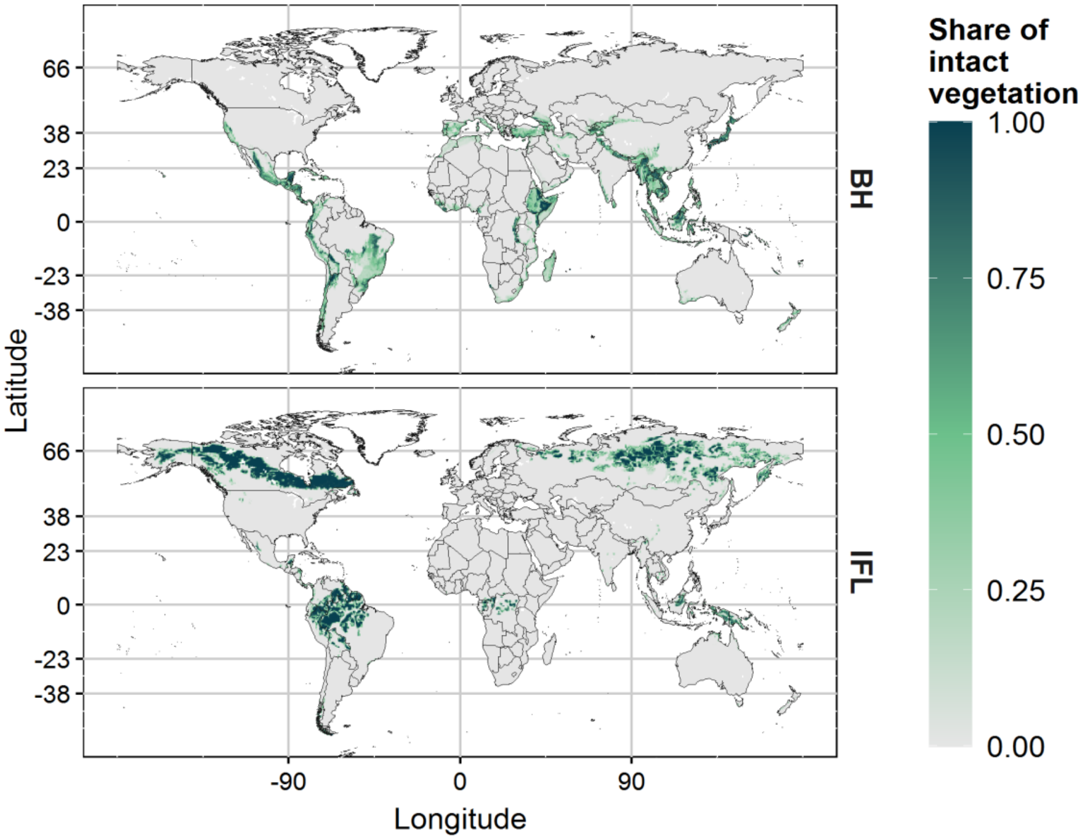

Supplementary Fig. 23: Conservation templates for the enlargement of protected areas. Spatially explicit information for the reactive and proactive conservation schemes are based on the biodiversity hotspots[43] (BH, top) and intact forest landscapes[44] (IFL, bottom) maps (see Methods).

3.1.3 Area Potentially Suitable for Cropland

Data on cropland potential has been derived from Zabel et al.[8] who have assessed suitable areas (suitability index from 0 to 100) for cropping based on climatic, soil and topographic conditions. In this analysis we use potentials for agriculture under current climatic conditions (1981 – 2010), since the evaluation of climate change impacts on suitable cropland is beyond the scope of this study. Yield variations are not directly derived from this data, but derived separately from the crop growth model LPJmL. The data is acquired at a spatial resolution of 30 arc seconds and transformed into a binary map of the same resolution after applying a suitability threshold with suitable (1) and unsuitable (0) pixels for cropland. The suitability threshold is set at a suitability index of 13 (out of 100), which excludes the lowest tertile of total marginal land potentially suitable for agriculture (suitability index between 0 and 33) from cropping activities in our model simulations. By defining the suitability threshold in this way we find the highest correlation (R-squared: 86.8 %) between modelled spatial cropland patterns (1995 to 2015) and cropland reported by the LUH2v2 data set, while ensuring sufficient flexibility for optimisation. In the ensuing processing steps, we transfer the data from 30 arc seconds to 0.5 degree by aggregating suitable cropland pixels at the high resolution to derive the area potentially suitable for cropping at 0.5 degree level (see Supplementary Fig. 24).

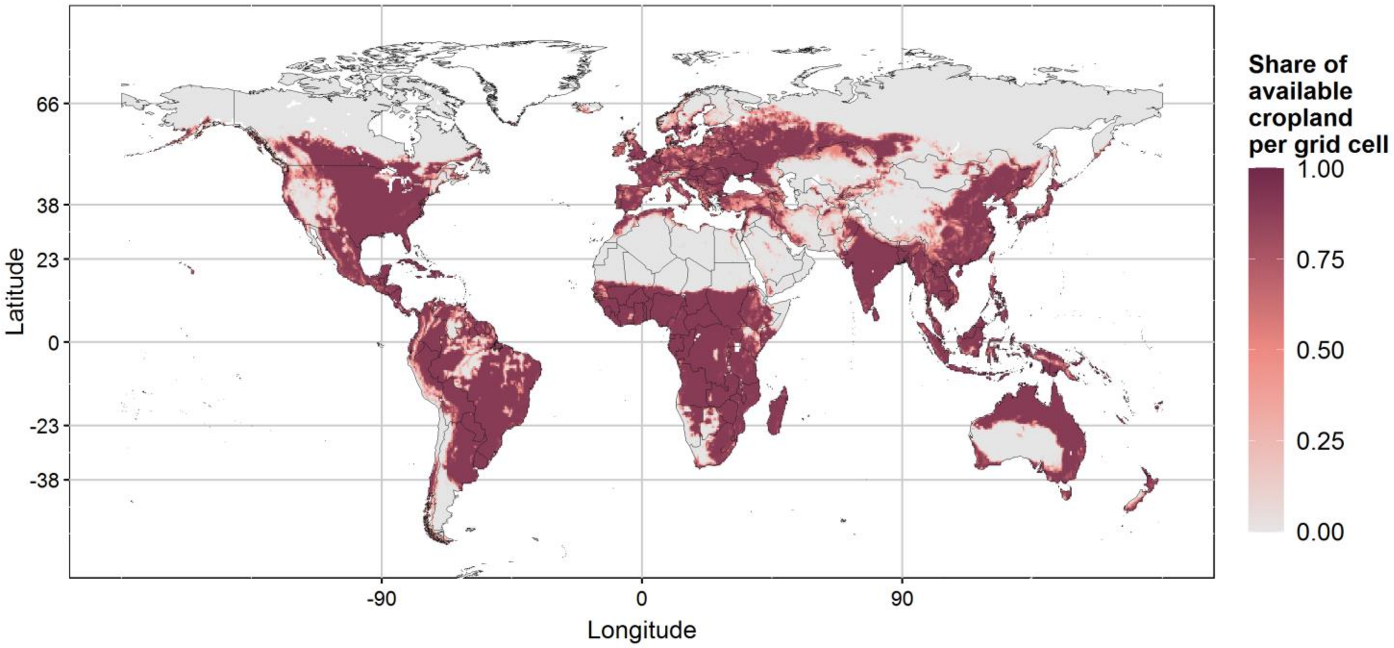

Supplementary Fig. 24: Share of area potentially suitable for cropland per grid cell in terms of total land area. Spatially explicit data on cropland potential was derived from Zabel et al.[8] at a spatial resolution of 30 arc seconds and processed to inform MAgPIE about potential areas for cropland expansion at the 0.5 degree level.

3.1.4 Yields

Crops grown in cropland areas include 19 food/feed crop types (e.g. temperate and tropical cereals, maize, rice, oil crops, roots), both rainfed and irrigated systems, and two 2nd generation bioenergy crop types (bioenergy trees and grasses). Biophysical annual average yield potentials for these crop types are derived from dedicated LPJmL5[15,16] simulations assuming that all crops are grown in all grid cells to determine potential crop productivity, including in areas where they are currently not cultivated, and to inform shifts in cropping areas. While LPJmL provides crop yields with unlimted N-supply, MAgPIE intends to represent actual crop yields. Therefore crop yields are regionally calibrated to match FAO crop production levels[17] in the initial time step using a scaling approach detailed in Heinke et al.[18] (see Supplementary Fig. 25). The temporal development of agricultural yields is modelled endogenously as a results of research & development (R&D) investments in yield-increasing technological change (TC), expressed by the τ-factor (Supplementary Fig. 13). The implementation is based on the effectiveness of R&D investments on yield changes (investment–yield ratio) with a time lag of 15 years, before yield increases are achieved[1]. The investment-yield ratio is empirically derived based on yield trends from FAO [17], and data on public and private R&D investments from IFPRI[19], and from the GTAP data base, version 7[20]. Investments into TC induce higher yields, but also increase the intensity of cropland use. This in turn raises the costs for further yield increases.

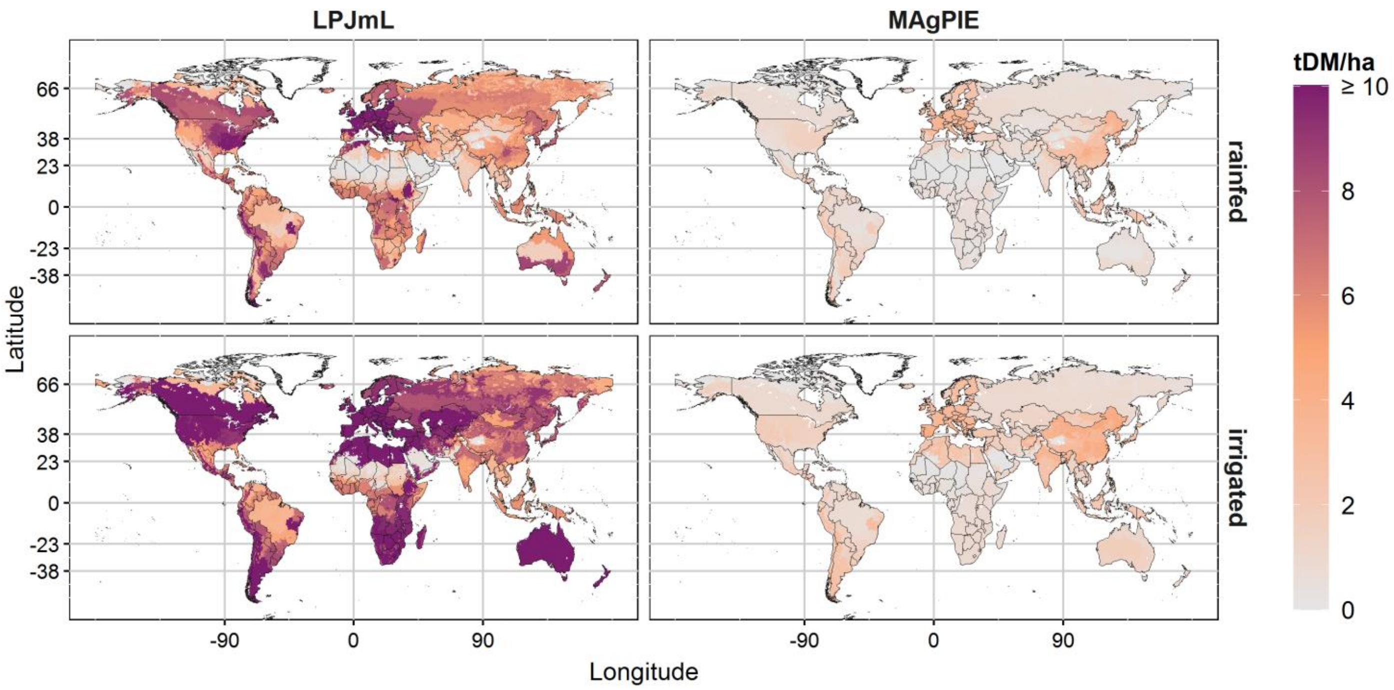

Supplementary Fig. 25: Exemplified temperate cereal yields in rainfed and irrigated production systems. Left hand panels show potential yields based on LPJmL, while the right-hand panels show MAgPIE yields after calibration in the initial time step to match production data from FAOSTAT[17].

3.1.5 Carbon

MAgPIE used grid cell-specific carbon densities for all modelled land-use types (cropland, pasture, primary forest, secondary forest, forestry, other land). The carbon densities for vegetation, soil and litter for each land-use type are derived from LPJmL at 0.5 degree resolution and aggregated to the spatial simulation units (clusters). Carbon densities for pasture, primary forest, secondary forest, forestry and other land are directly derived from LPJmL (Supplementary Fig. 26) based on grid cell-specific environmental drivers such as temperature, precipitation, soil properties or atmospheric CO_2 concentration. Carbon densities for cropland are also derived from LPJmL, but litter carbon density is assumed to be 0. Moreover, cropland soils have a lower soil organic matter (SOM) content after land conversion from natural vegetation. Therefore, carbon densities in cropland areas are reduced in relation to climatic conditions by 20-52 %[21], as compared to simulated soil carbon below intact land cover in LPJmL[22].

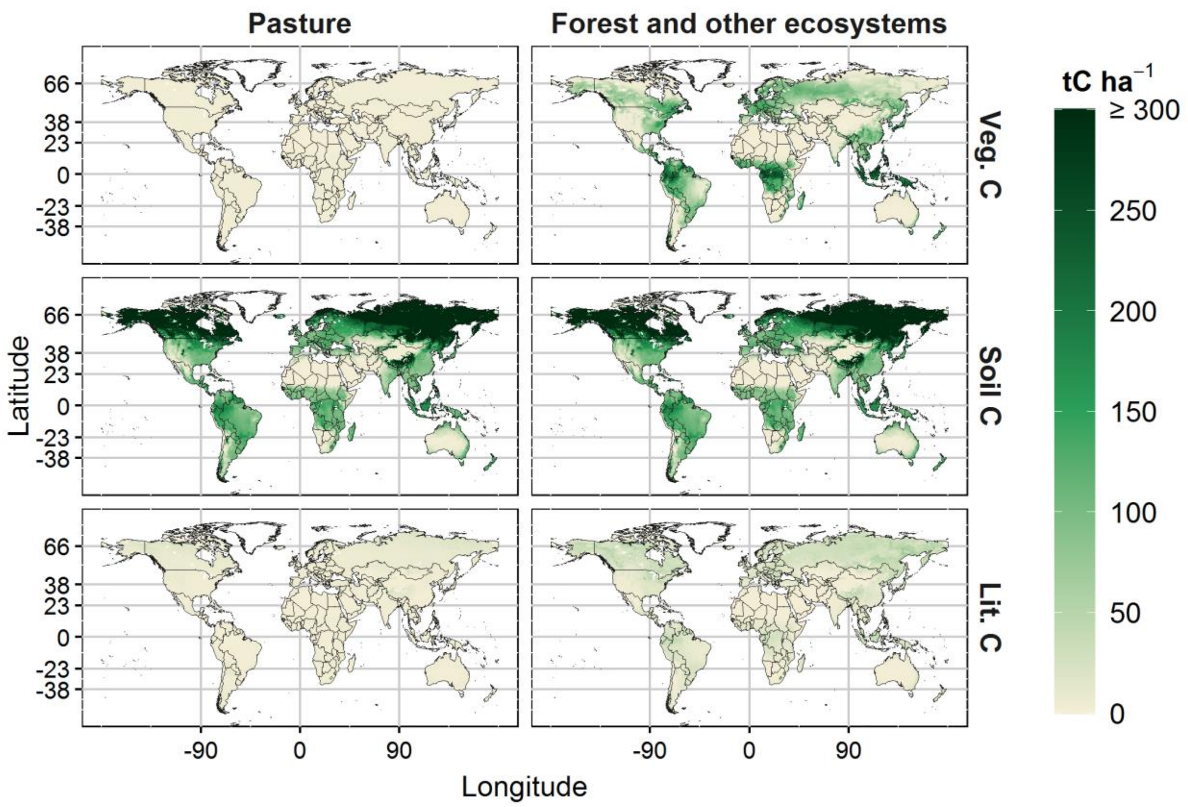

Supplementary Fig. 26: Potential carbon density (tC ha^{-1}) for pasture, as well as forest and other ecosystems as derived from LPJmL and separated into vegetation, litter and soil carbon.

3.1.6 Scenario Inputs

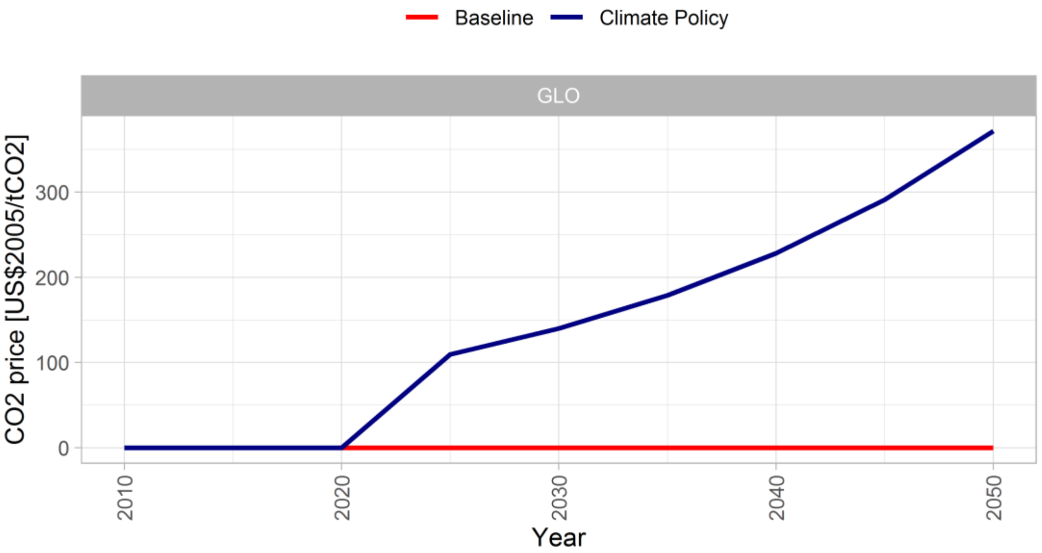

Supplementary Fig. 27: Carbon price trajectory in the SSP2 baseline (red) and climate policy (blue) scenarios as derived with the coupled REMIND-MAgPIE modelling framework [4]. The estimated carbon price trajectory in the climate policy scenario (blue) is consistent with the 1.5 °C target from the Paris Agreement across the global economy, energy and land-use system.

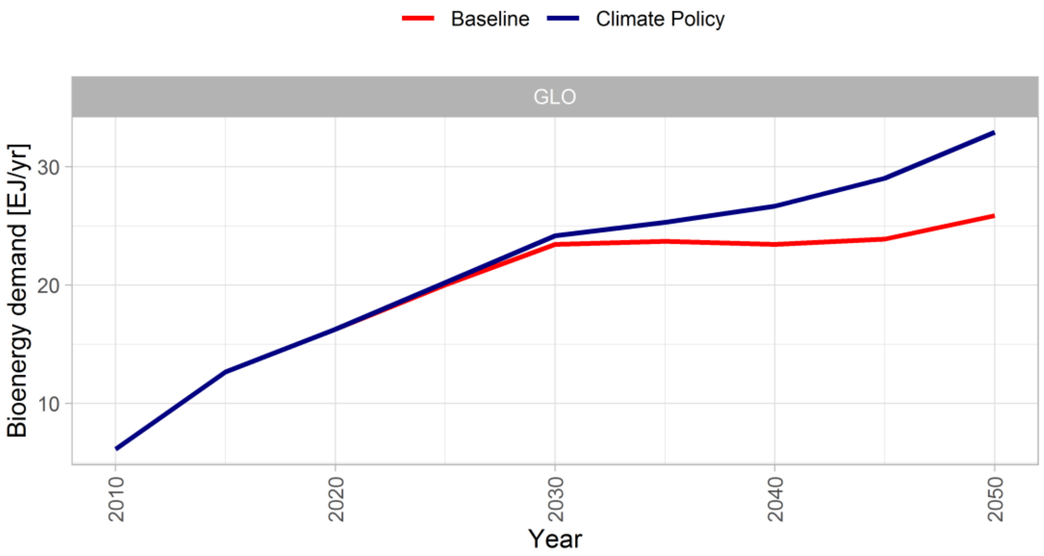

Supplementary Fig. 28: Bioenergy in the SSP2 baseline (red) and climate policy (blue) scenarios. As this study's main scope was to assess the effects of enhanced terrestrial carbon sequestration (NCP 4: climate regulation) we used a conservative bioenergy trajectory as specified in nationally determined contributions (NDC).

3.2 Spatial Economic Allocation Landscape Simulator (SEALS)

The spatial relation between sites of NCP supply and areas where benefits from NCP are realised is a key element in assessing many NCP that are critical to future land productivity and the resilience of (agro-)ecosystems[23,24]. However, at the spatial scales at which MAgPIE operates in its original standalone application, these relations cannot be mapped spatially to the degree necessary for assessing changes in the supply of important NCP that result from global land-use dynamics. For this reason, we employ the SEALS model to allocate land-use changes at 0.5 degree resolution in a globally consistent, replicable and empirically calibrated way to a spatial resolution that is suitable for assessing changes in crucial NCP that require landscape-scale information. As compared to earlier versions of the SEALS model that only modelled the expansion of single land-use types[25], the version used in this study has the capacity to consider changes across multiple land-use types simultaneously, based on advances documented in Johnson et al.[26] and Johnson et al.[27]

3.2.1 Allocation Algorithm

LULC classification. The simplified land-use, land-cover (LULC) classification scheme in the SEALS model is hierarchically defined in accordance with the ESA-CCI LULC types (see Supplementary Table 6). This thematic simplification was done to conform to the LULC classification of MAgPIE, which uses seven land-use types, including three separate forest classes. The MAgPIE forest classes were grouped into one aggregated forest class, because they were not treated statistically different in the SEALS model.

Supplementary Table 6: Relation between MAgPIE, SEALS and ESA-CCI LULC classes.

Id	SEALS LULC types	Combined ESA-CCI LULC types	MAgPIE LULC types
1	Urban	190	Urban
2	Cropland	10, 11, 12, 20	Cropland
3	Grassland/pasture	130	Pasture
4	Forest	40, 50, 60, 61, 62, 70, 71, 72, 80, 81, 82, 90, 100, 151, 160, 170	Primary forest, secondary forest, forestry
5	Non-forest vegetation	30, 110, 120, 121, 122, 140, 150, 151, 152, 153, 180	Other land
6	Water	210	-
7	Barren	200, 201, 202, 220	-
NA	No data	255	-

Land cover allocation. SEALS identifies the net change in each land-use type at the 0.5 degree resolution and produces a net change vector n_c, in which each entry represents the net change of the *i*-th land-use type in the grid cell c. SEALS then uses a m by i matrix of coefficients that describe how each m-th spatial information affects the expansion probability of the i-th land use type in each grid cell. The coefficients in this matrix were empirically derived by iteratively applying the allocation model on a time series of LULC maps for the years 2000 to 2010 from ESA-CCI [28]. The coefficients that were most predictive on withheld data (2011-2015) were then selected to be used for future LULC projections.

SEALS land allocation procedure.

1. Define a vector n_c, which includes the net hectarage change for each *i*-th LULC class that might expand in each *c*-th 0.5 degree grid cell.
2. Define the starting condition of the landscape based on the current 300 meter resolution ESA-CCI LULC map, where $L_{x,t}$ defines the starting condition in pixel x and time t.
3. The spatial allocation algorithm is then defined by $S(n_c, L_{c,x,t}, p_{x,i}, a_{x,i,j}, e_x) = L_{x,t+1}$ which takes the net area change n_c and an existing LULC map ($L_{x,t}$) and produces a LULC map for a future time step based on three factors:
   i. $p_{x,i}$ the physical suitability of pixel *x* to be converted into LULC class *i*
   ii. $a_{x,i,j}$ the adjacency effect on the pixel conversion suitability to LULC class *i* in cell *x* based on the relative adjacency impact of LULC class *j*.
   iii. e_x a binary map that defines which grid cells are eligible (e.g. prevent expansion into cities or water bodies).
4. Combine 3.i - 3.iii to define the weighted suitability map $C_{x,i,j}$
5. Rank all values in $C_{x,i,j}$ (note, this is where much of the computation time happens) into a map of conversion order *R* (lower values denote earlier conversion).
6. Starting with the first conversion in *R*, convert to the target LULC class and reduce the remaining amount of conversion necessary in n_c by the amount converted. Continue until $n_c = 0$ in all 0.5 degree grid cells.

3.2.2 Model Calibration

The SEALS model operates based on spatial relations observed in ESA-CCI's 1992 to 2015 time series of high-resolution LULC maps. The land allocation coefficients were calibrated following an iterative Gaussian L1-loss function minimisation approach. The approach uses the following algorithm:

1. Define a baseline condition (year 2000 for this example).
2. Define a projection year in the set of observed years after the baseline year (2010) and aggregate the net-change between the two years for each coarse resolution (0.5 degree) grid-cell. This defines the amount of change in each LULC class that our allocation algorithm will predict.
3. Allocate the net change of each LULC class using only the baseline map and a spatial allocation algorithm, $S(p1)$, where $p1$ is the parameter set used in the allocation and is initially set to an arbitrary value.
4. Calculate how accurate the projected LULC map for 2010 is compared to the observed 2010 LULC map. Specifically, compute the difference score, which is the summation of five L1-difference functions, one for each LULC transition that calculates how different (in terms of Gaussian-blurred distance) each class is in the projected map compared to the observed map. This generates a score for the quality of fit for the current set of parameters.
5. Iteratively for each parameter in $p1_i$ of the *i*-th land use type, increase the parameter by a given percentage (initially 10 %), rerun step 4 with the new parameter, observe the new similarity score, then decrease it by 10 % and rerun.
6. After calculating the change in fit from each parameter increase and decrease in step 5, identify which change had the greatest improvement in the similarity score. Update the parameter set to include the single best change, and then repeat steps 3-6 until no additional improvements can be made.

3.2.3 Calibrated Parameters

The results from the calibration exercise are reported in Supplementary Table 7. The first column is the name of the input regressor, while the values in columns 2-6 are the optimized values from the calibration algorithm for the five types of observed land-use changes. Of the regressors listed, "presence" variables simply are a 0-1 variable derived from the LULC map on what is the baseline class in 2015. The adjacency regressors are a Gaussian convolution of the presences maps with different sigmas chosen to represent nearby adjacency impact vs. adjacency effects that have impact further away. To make this definition more intuitive, we denote the distance of the adjacency effect by the number of kilometres away where the effect falls below 5%. Finally, at the end of the list of regressors are all the static variables used as inputs to the regression, described in the main manuscript.

Supplementary Table 7: Parameters derived during calibration of the SEALS model.

Regressor Name	urban	agriculture	grassland	forest	non-forest natural
urban presence	0.00000	-0.03222	0.01389	-0.01389	-0.01667
cropland presence	-0.02778	0.00000	0.01667	0.01111	0.00433
grassland presence	0.00556	0.01889	0.00000	0.04167	-0.02611
forest presence	-0.01944	-0.01667	-0.00267	0.00000	0.03344
nonforestnatural presence	0.01000	0.14444	0.06011	0.02000	0.00000
water presence	0.00000	0.00000	0.00000	0.00000	0.00000
barren and other presence	-1.11944	0.00167	0.12667	0.06111	-0.02333
urban gaussian adjacency 1km	1.71389	-1122.23344	-11.13056	0.04167	-1122.24167
cropland gaussian adjacency 1km	0.10556	0.33344	0.02222	0.00000	-11.24444
grassland gaussian adjacency 1km	0.05444	0.00544	0.38000	0.01822	0.08556
forest gaussian adjacency 1km	-0.12222	1111.06556	-0.01100	1.55500	-0.02223
nonforestnatural gaussian adjacency 1km	0.01089	0.00000	0.01944	-0.12222	0.46667
water gaussian adjacency 1km	0.03656	-112.20278	-112.24000	-1.10556	-1133.32233
barren and other gaussian adjacency 1km	-0.12778	-112.25556	-1112.14444	-1111.13333	0.00556
urban gaussian adjacency 5km	-0.07222	-11.52222	-111.26389	-0.09333	-0.08778
cropland gaussian adjacency 5km	0.06889	0.16233	-0.01667	0.12222	0.06556
grassland gaussian adjacency 5km	0.10022	-0.02500	0.43111	-0.02668	-0.04167
forest gaussian adjacency 5km	0.13322	0.36778	0.07633	0.68888	0.11333
nonforestnatural gaussian adjacency 5km	0.00000	-0.07311	0.02444	-0.00556	0.15278
water gaussian adjacency 5km	0.09167	0.00500	-1111.10544	-1.09278	-0.00222
barren and other gaussian adjacency 5km	0.04556	0.15000	-1111.07778	-0.00833	-110.89000
soil organic content	0.02778	-0.15000	110.97778	-111.14000	-0.02778
precip mm	11.11944	-0.99444	1.14000	-1.07500	11.00444
alt m	-0.10444	0.08500	-0.02489	-0.03779	0.01000
temperature c	-0.02211	0.04444	-0.01111	-0.01000	-0.00111
travel time to market mins	0.01612	0.21000	0.00556	10.55500	-0.03433
pop	0.00000	0.00000	0.00000	0.00000	0.00000
soil bulk density	1.15000	1.12222	-0.01667	22.18444	-11.10000
soil cec	0.00000	-0.01667	0.00000	111.07222	0.00000
clay percent	-0.05111	0.02000	-0.18611	-0.04622	1.08433
ph	0.00000	0.10000	0.00000	0.00000	0.00000
sand percent	0.03444	0.01833	-0.03778	-0.04889	-0.00111
silt percent	-0.01278	-0.16500	0.00000	-0.05901	-0.14611

3.2.4 Current Limitations

The implementation of SEALS used here has several limitations. First, it is an allocation algorithm for downscaling land-use change data and is not a full land-use change model such as Dyna-CLUE[29], CLUMondo[30] or Dinamica EGO[31]. This means it ignores certain factors relevant to land-use change prediction, including dynamic agglomeration effects (such as when new growth of certain land-uses dynamically modify the probability of further local expansion), interaction among agents (e.g. as in a cellular-automata or agent-based simulation approach), or more detailed dynamics of supply and demand for particular land-use changes, such as agricultural expansion. However, when linked with a model like MAgPIE, these limitations are greatly reduced insofar as the complex dynamics are modelled at the coarser resolution. The choice of SEALS is also justified by a more prosaic reason: due to computational challenges, few models of land-use change can be calculated at the global scale at sufficient resolution for ecosystem service analyses. Wolff et al.[30] is one of the models that gets close to this scale with 10km resolution results, but this resolution is not high enough for the field-scale analyses we do in this manuscript.

A second limitation is that although SEALS is computationally efficient enough to be calculated globally with sub-hour calculation time, calibration of the model requires running the allocation algorithm thousands or millions of times, which currently presents computational challenges we have not been able to overcome. As a result, the calibration procedure described above was run on a subset of globally-distributed 1-degree tiles of data. The values from the calibration runs were then averaged in order to get a final set of regression coefficients for running the global model. Further research could improve upon this by investing in computing resources, algorithmic improvement, or space-variable coefficients for downscaling.

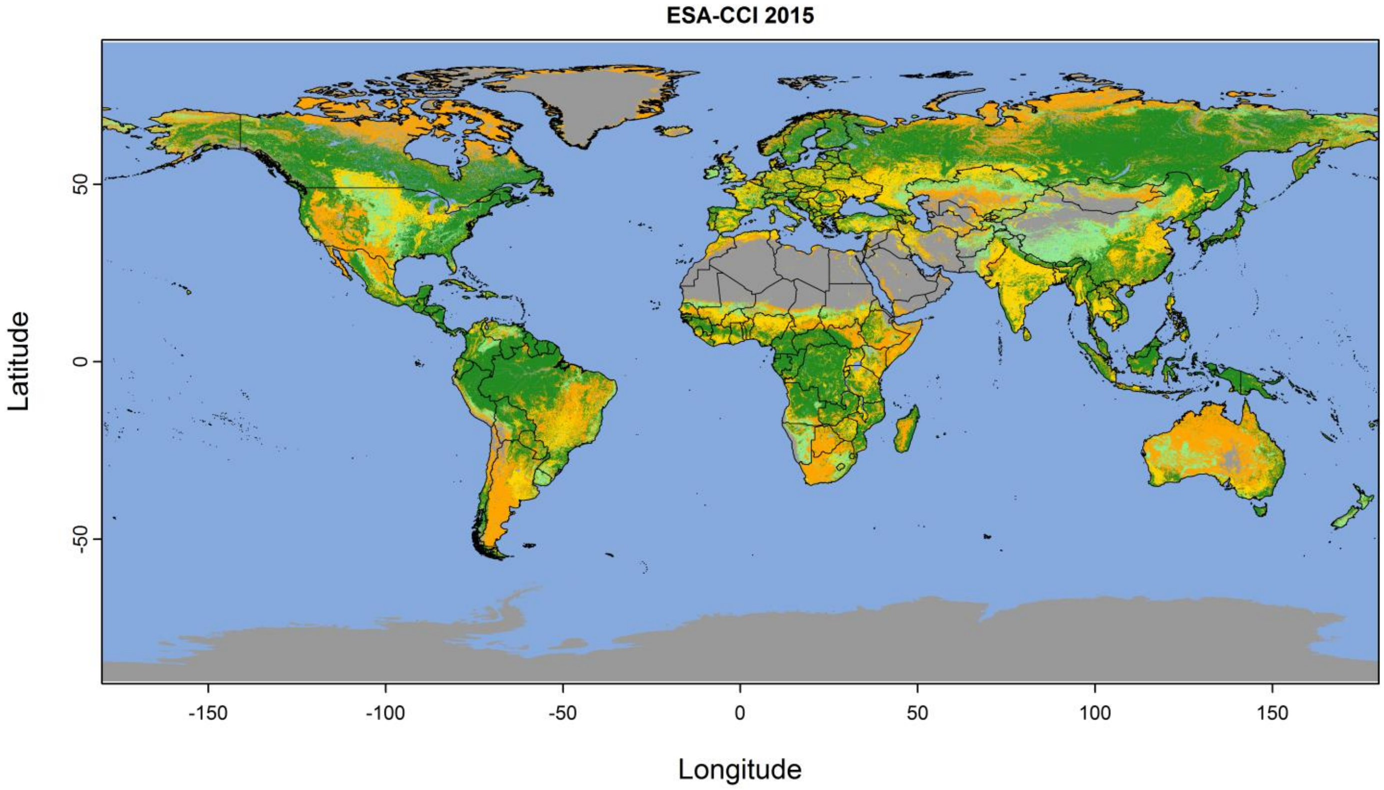

Supplementary Fig. 30: ESA-CCI LULC map (ESA, 2017) for the year 2015. Cropland: yellow, grassland/pasture: light green, forest: dark green, non-forest vegetation: orange, urban: red, water: blue, barren and ice: grey.

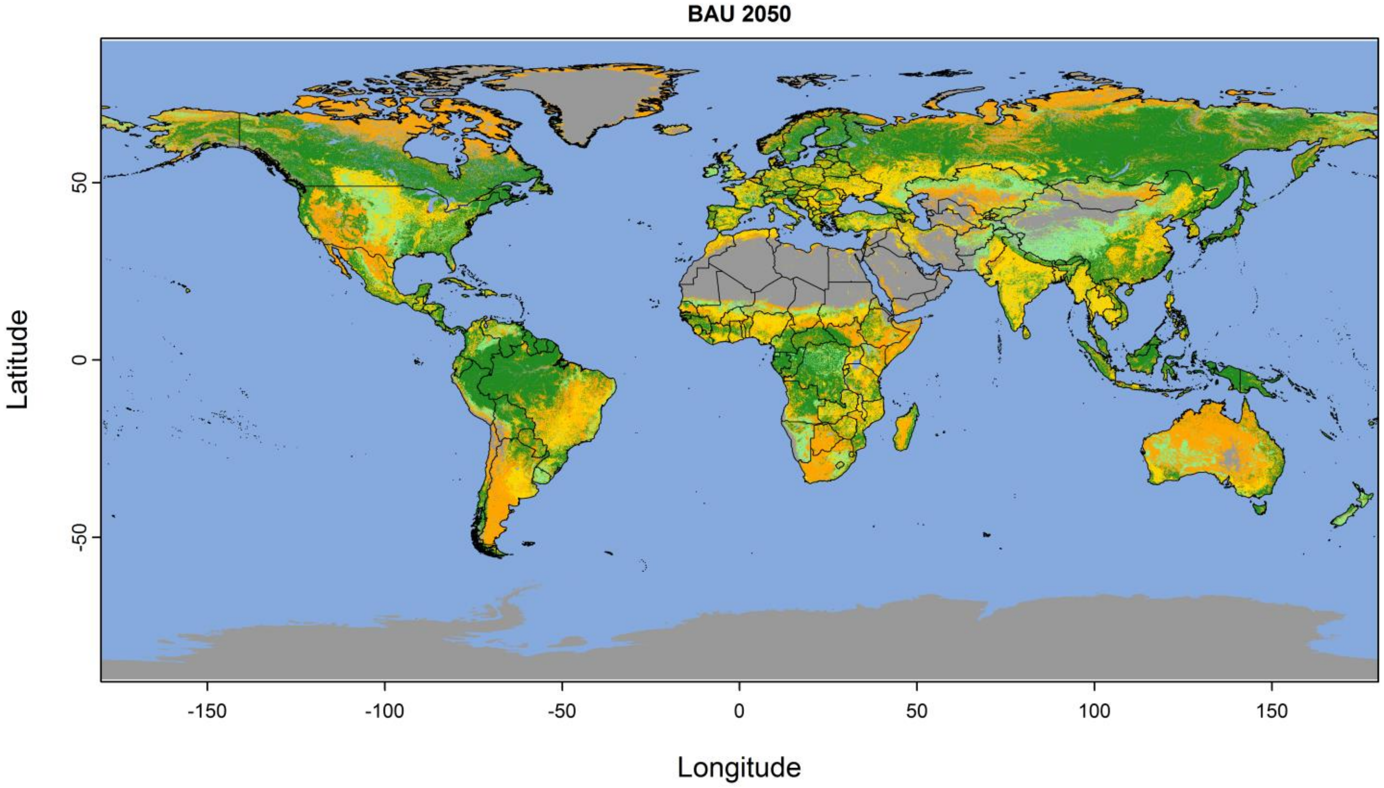

Supplementary Fig. 29: Projected high-resolution LULC for the year 2050 in the BAU scenario. Spatially-explicit land-use projections at 0.5 degree are based on MAgPIE output and transferred to a spatial resolution of 10 arc seconds by the LULC allocation model SEALS. Cropland: yellow, grassland/pasture: light green, forest: dark green, non-forest vegetation: orange, urban: red, water: blue, barren and ice: grey.

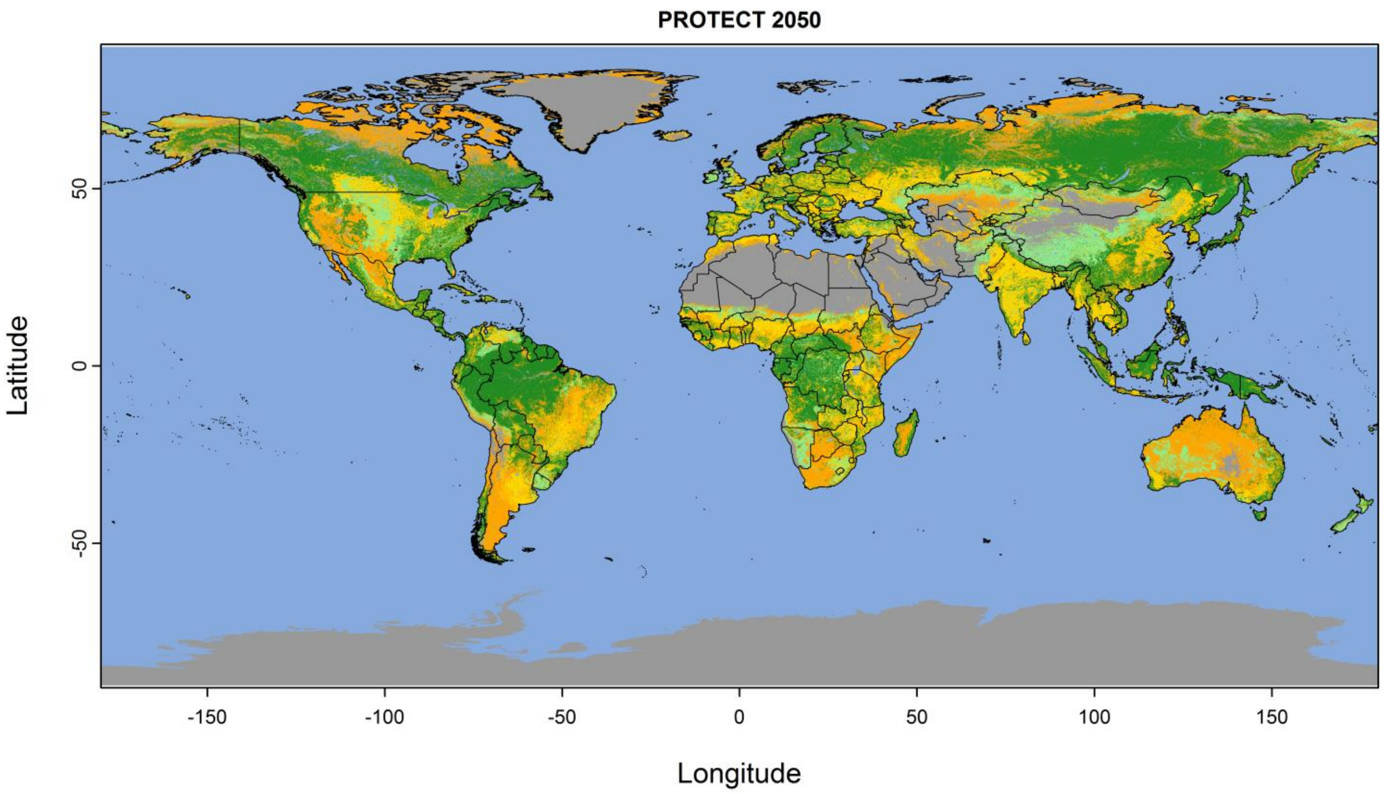

Supplementary Fig. 31: Projected high-resolution LULC for the year 2050 in the PROTECT scenario. Spatially-explicit land-use projections at 0.5 degree are based on MAgPIE output and transferred to a spatial resolution of 10 arc seconds by the LULC allocation model SEALS. Cropland: yellow, grassland/pasture: light green, forest: dark green, non-forest vegetation: orange, urban: red, water: blue, barren and ice: grey.

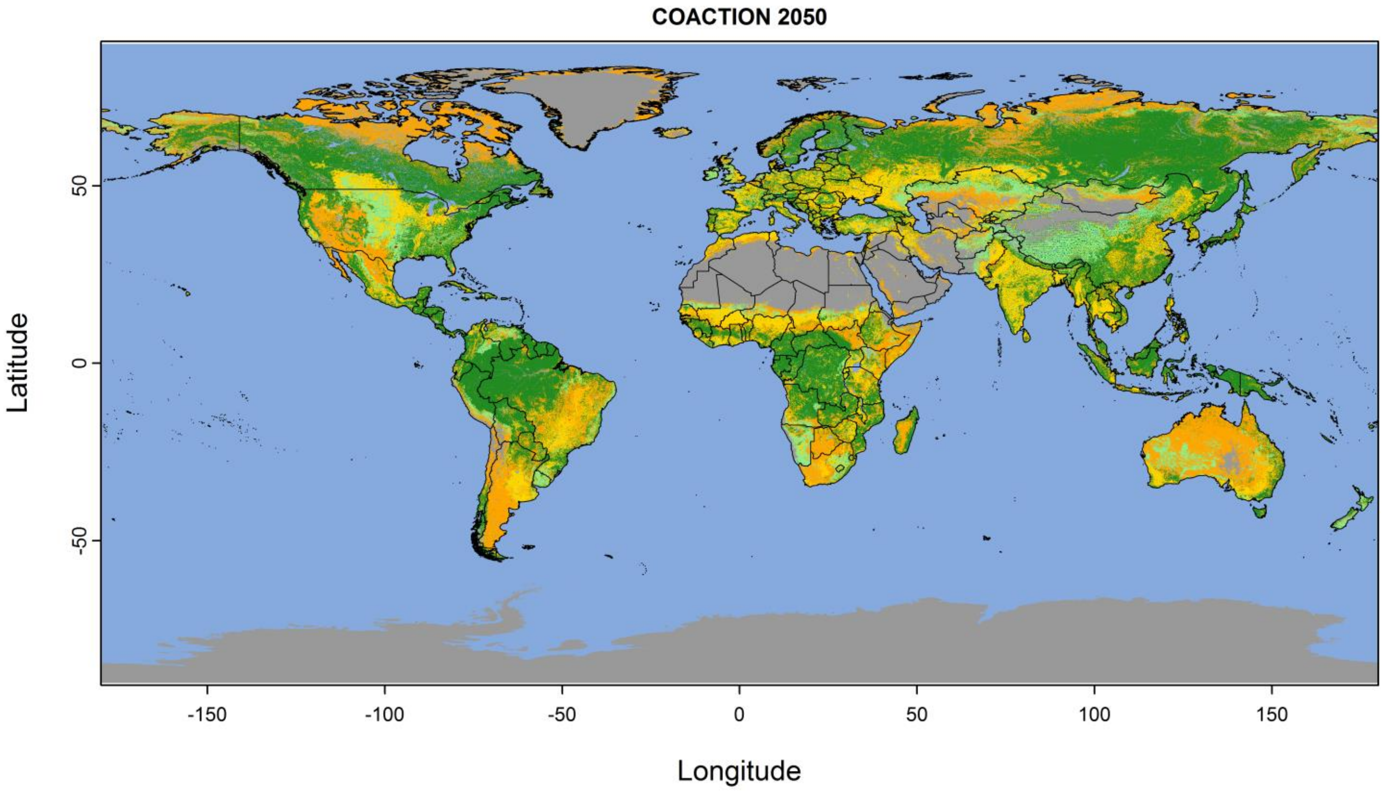

Supplementary Fig. 32: Projected high-resolution LULC for the year 2050 in the COACTION scenario. Spatially-explicit land-use projections at 0.5 degree are based on MAgPIE output and transferred to a spatial resolution of 10 arc seconds by the LULC allocation model SEALS. Cropland: yellow, grassland/pasture: light green, forest: dark green, non-forest vegetation: orange, urban: red, water: blue, barren and ice: grey.

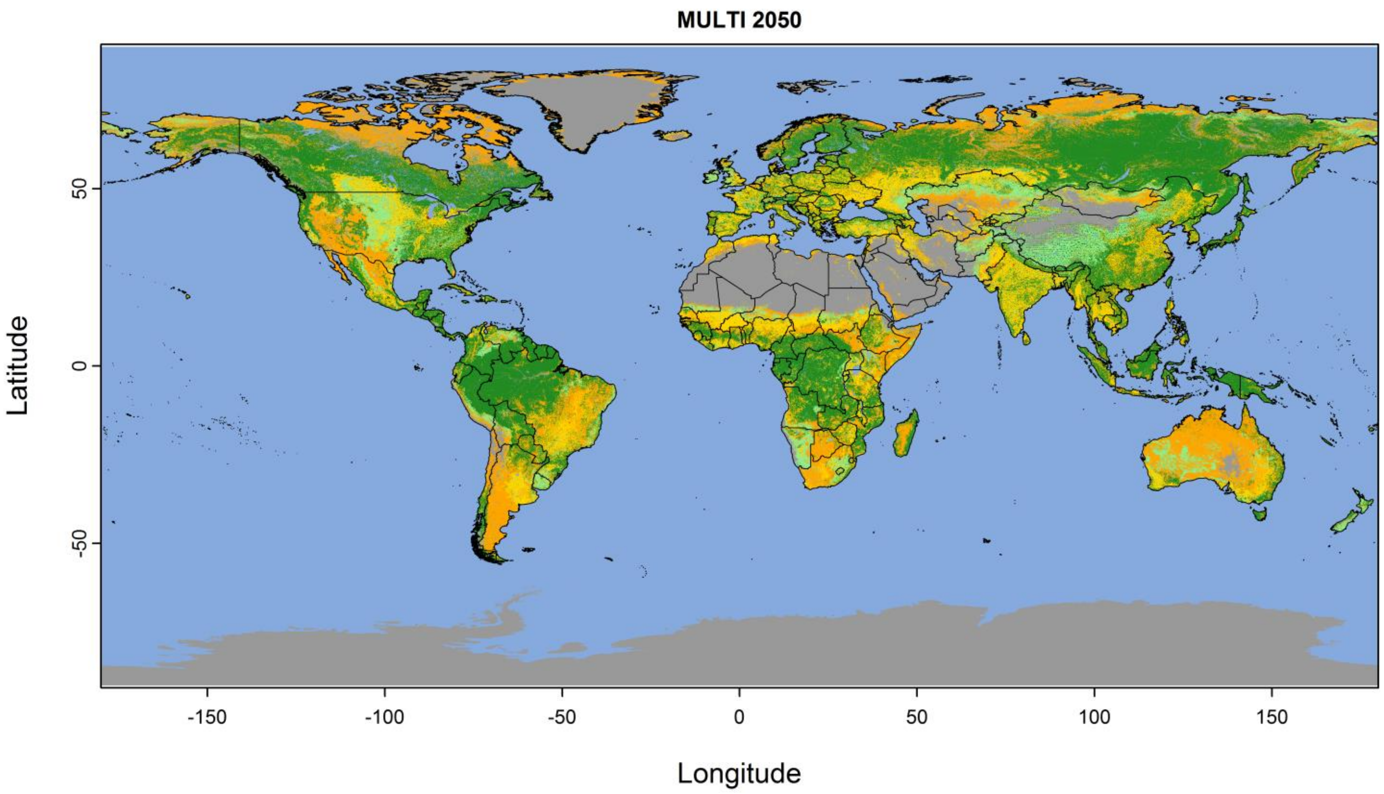

Supplementary Fig. 33: Projected high-resolution LULC for the year 2050 in the MULTI scenario. Spatially-explicit land-use projections at 0.5 degree are based on MAgPIE output and transferred to a spatial resolution of 10 arc seconds by the LULC allocation model SEALS. Cropland: yellow, grassland/pasture: light green, forest: dark green, non-forest vegetation: orange, urban: red, water: blue, barren and ice: grey.

3.3 Global Soil Erosion Modelling (GloSEM)

3.3.1 Revised Universal Soil Loss Equation (RUSLE)

The Revised Universal Soil Loss Equation (RUSLE) is an empirical, detachment-limited model of sheet and rill erosion. In the RUSLE model, the quantity of sediment transport, which theoretically could be infinite, is limited by the soil detachment capacity as a result of the rainfall erosivity. The annual average soil erosion A (Mg ha^{-1} yr^{-1}) is given by

$$A = R \cdot L \cdot S \cdot K \cdot C \cdot P \qquad \text{Eq. 1}$$

where R is the rainfall-runoff erosivity factor (MJ mm h^{-1} ha^{-1} yr^{-1}), K is the soil erodibility factor (Mg h MJ^{-1} mm^{-1}), L is the slope length factor (dimensionless), S is the slope steepness factor (dimensionless), C is the land cover and management factor (dimensionless) and P is the soil conservation or prevention practices factor (dimensionless).

3.3.2 GloSEM Implementation

GloSEM employs a Geographic Information System (GIS) based raster approach of the multiplicative RUSLE model for spatially explicit soil loss estimation. For this study, the spatial resolution (ca. 300 meter cell size at the equator) has been adjusted to match the land cover maps produced by the SEALS land allocation model (chapter S3.2 above). With regard to soil erosion, we assume each cell to be independent from the others and refer to soil loss as the amount of sediment that reaches the lower boundary of the area covered by each cell in a given time.

The derivation of the rainfall-runoff erosivity (R), soil erodibility (K), slope length (L) and slope steepness (S) factors in Eq. 1 is described in detail in Panagos et al.[32], Borrelli et al.[33] and Borelli et al.[34]. In this study we assume no changes over time in the Earth's surface current exposure to the kinetic energy input of rainfall, as this was beyond the scope of this study. We also did not consider changes in soil conservation or prevention practices. Therefore, we assumed the P-factor to remain constant at 1.

Regarding the estimation of the land cover and management factor C, we used differing approaches for cropland, forest and non-forest vegetation cover and modified the procedure detailed in Borrelli et al.[33] and Borelli et al.[34] to conform to the MAgPIE-SEALS framework. These approaches are detailed in the following.

3.3.3 Land Cover and Management Factor (C)

Cropland area. In order to spatially explicitly describe typical cropping patterns, we used simulated patterns at 0.5 degree from MAgPIE for both the reference and future time steps. The MAgPIE output reports cultivated cropland area for 19 food/feed crop functional types in each of the 59199 0.5 degree grid cells. We also separated the cultivated area of the MAgPIE aggregate crop functional type "vegetables, fruits and nuts" into "vegetables" and "fruits and nuts", based on current FAO production data[17]. We derived the share of each of the resulting 20 crop functional types (Supplementary Table 8) relative to the total cropland area in each grid cell and then estimated the overall C-factor value by applying

$$C_{\text{Cropland}} = \sum_{n=1}^{20} C_{\text{Crop}_n} \cdot [\%]\text{Cell}_{\text{Crop}_n} \qquad \text{Eq. 2}$$

where C_{Cropland}denotes the overall C-factor for cropland in each grid cell, C_{Crop_n} is the C-factor of the n-th crop type and $[\%]\text{Cell}_{\text{Crop}_n}$ is the area share of each n-th crop type in the total cropland area of each grid cell.

Supplementary Table 8: Crop types included in the calculation of soil erosion and their respective C-factor values as derived from the literature.

Crop type	C-factor value
Temperate cereals	0.2
Maize	0.38
Tropical cereals	0.2
Rice	0.15
Soybean	0.32
Rapeseed	0.25
Groundnut	0.25
Sunflower	0.25
Oil palm	0.15
Pulses	0.32
Potato	0.34
Cassava sp.	0.34
Sugar cane	0.15
Sugar beet	0.34
Vegetables	0.25
Fruits and nuts	0.1
Cotton	0.4
Fodder crops	0.15
Bioenergy grasses	0.1
Bioenergy trees	0.1

Non-cropland area. The C-factor values for forest, grassland and other non-forest vegetation is derived using a semi-qualitative approach based on a constraining range of C-factor values from the literature and subpixel information on annual vegetation and forest cover for each non-cropland cover pixel. The effect of annual vegetation and forest cover on C-factor estimates is determined based on global FCOVER[35] (fraction of green vegetation cover) data from the Copernicus Global Land Service[36] and tree cover data from Hansen et al.[37]. We downloaded and averaged ten time steps between 2014 and 2016 of the FCOVER information in order to account for the effects of the inter-annual rainfall variability.

Both FCOVER and tree cover data sets represent the current condition of global vegetation and tree cover and include artefacts, e.g. in cropland areas. In order to capture the effects of forest expansion, e.g. due to carbon-price induced afforestation, and to obtain reliable estimates of potential vegetation and tree cover in degraded areas currently not covered by intact vegetation or trees, we applied separate random forest algorithms[38,39] to estimate potential vegetation and tree cover at the global scale. The covariates for the models were downloaded from the WorldClim data

base[40] at a spatial resolution of 30 arc seconds. Modelled potential vegetation and tree cover was therefore initially mapped at 30 arc seconds and subsequently disaggregated to the final spatial resolution of 10 arc seconds. We selected the following environmental covariates for training and model prediction:

- Bio 1: Annual mean temperature (C°)
- Bio 11: Mean temperature of the coldest quarter (C°)
- Bio 12: Annual precipitation (mm)
- Elevation (meters above mean sea level)
- Mean annual solar radiation (kJ m^{-2} day^{-1})
- Mean annual vapour pressure (kPa)

The performance of the random forest models was assessed on a withheld data sample via the root mean square error (RMSE) for FCOVER and tree cover values (0 to 1) and the amount of variation explained by the model (R-squared)[41]. The model for mapping potential FCOVER produced a RMSE of 0.06 and an R-squared of 96.4 %. The potential tree cover model also produced accurate predictions, but had a slightly lower score with RMSE at 0.11 and an R-squared of 91.4 %. We then used these models to fill in degraded pixels in the original mean FCOVER and tree cover data sets. The resulting continuous FCOVER and tree cover layers are shown in Supplementary Figs. 34 and 35.

The C-factor values for non-forest vegetation and grassland cover pixels was then calculated as

$$C_{\mathrm{Veg}} = \mathrm{MIN_C} + \left((\mathrm{MAX_C} - \mathrm{MIN_C}) \cdot \left(1 - \mathrm{FCOVER_p}\right)\right) \qquad \text{Eq. 3}$$

where C_{Veg} is the C-factor value for vegetated land pixels, $\mathrm{MIN_C}$ is the lower, while $\mathrm{MAX_C}$ is the upper bound for C-factor values expected in vegetated surfaces and $\mathrm{FCOVER_{Px}}$ is the fractional vegetation cover within pixel p. $\mathrm{MIN_C}$ and $\mathrm{MAX_C}$ were defined according to values from the literature (Supplementary Table 9).

For forest cover the C-factor value was estimated in a two-step approach. First, a preliminary C-factor value C_{Tree} in forested pixels is calculated by

$$C_{\mathrm{Tree}} = \mathrm{MIN_C} + \left((\mathrm{MAX_C} - \mathrm{MIN_C}) \cdot \left(1 - \mathrm{TC_p}\right)\right) \qquad \text{Eq. 4}$$

where $\mathrm{MIN_C}$ is the lower, while $\mathrm{MAX_C}$ is the upper bound for C-factor values expected in forested pixels and $\mathrm{TC_p}$ is the tree cover share in each pixel. The overall C-factor in forested pixels was then derived by

$$C_{\mathrm{Forest}} = C_{\mathrm{Tree}} + \left(\left(C_{\mathrm{Veg}} - C_{\mathrm{Tree}}\right) \cdot \left(1 - \frac{\mathrm{TC_p}}{\mathrm{FCOVER_p}}\right)\right) \qquad \text{Eq. 5}$$

where C_{Forest} is the overall C-factor in forested pixels. In some cases $\mathrm{TC_p}$ was reported at a higher value than $\mathrm{FCOVER_p}$. In these instances $\frac{\mathrm{TC_p}}{\mathrm{FCOVER_p}}$ was set to 1.

Supplementary Table 9: Lower and upper bounds for C-factor values expected in vegetated surfaces.

**Land-cover type**	$\mathbf{MIN_C}$	$\mathbf{MAX_C}$
Forest	0.0001	0.009
Grassland & Non-forest vegetation	0.01	0.125

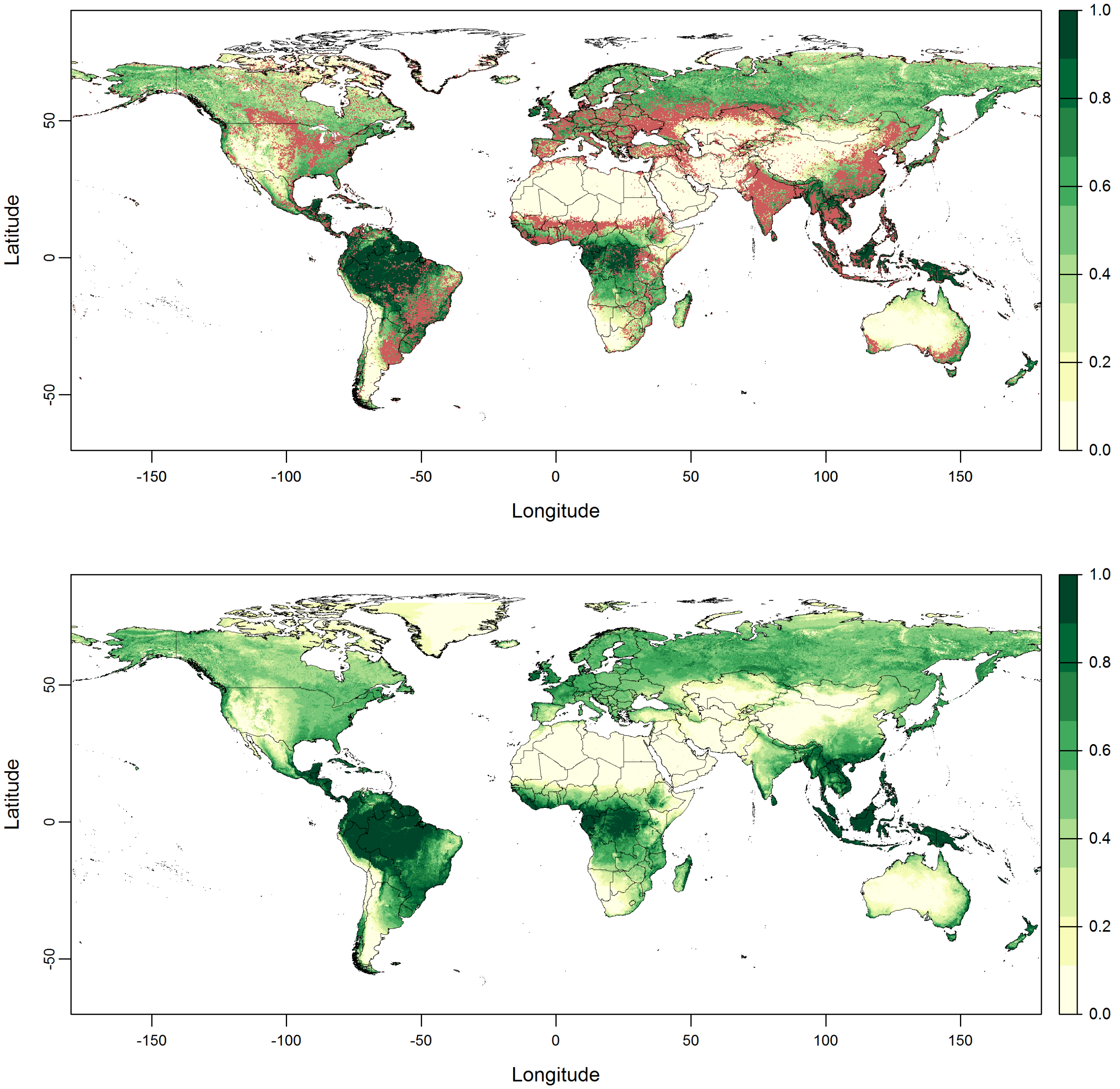

Supplementary Fig. 34: Mean annual fractional vegetation cover (FCOVER) for the reference year 2015 as derived from the Copernicus Global Land Service (top) and modelled potential mean annual FCOVER (bottom). Red areas in the top panel show degraded areas that were filled with modelled values in the bottom panel.

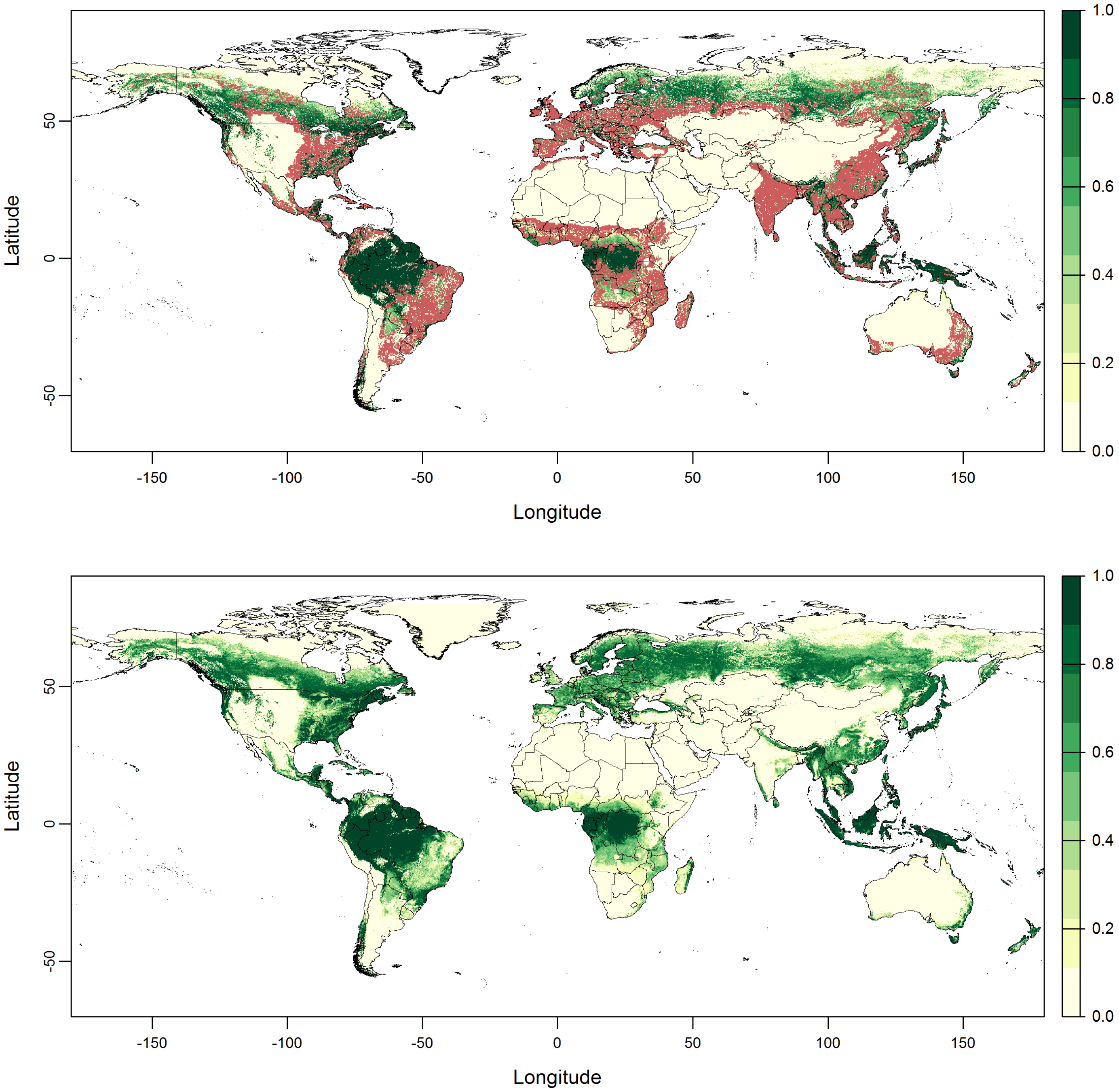

Supplementary Fig. 35: Global tree cover as derived from Hansen et al. (2013) (top) and modelled potential global tree cover (bottom). Red areas in the top panel show deforested and degraded areas that were filled with modelled values in the bottom panel.

4 Supplementary References

1. Dietrich, J. P., Schmitz, C., Lotze-Campen, H., Popp, A. & Müller, C. Forecasting technological change in agriculture—An endogenous implementation in a global land use model. *Technological Forecasting and Social Change* **81**, 236–249 (2014).
2. Popp, A. *et al.* Land-use futures in the shared socio-economic pathways. *Global Environmental Change* **42**, 331–345 (2017).
3. Schmitz, C. *et al.* Trading more food: Implications for land use, greenhouse gas emissions, and the food system. *Global Environmental Change* **22**, 189–209 (2012).
4. Baumstark, L. *et al.* REMIND2.1: transformation and innovation dynamics of the energy-economic system within climate and sustainability limits. *Geoscientific Model Development* **14**, 6571–6603 (2021).
5. Nelson, A. *Travel time to major cities: A global map of Accessibility*. http://forobs.jrc.ec.europa.eu/products/gam/index.php (2008) doi:10.2788/95835.
6. Schaphoff, S. *et al.* LPJmL4 – a dynamic global vegetation model with managed land – Part 2: Model evaluation. *Geosci. Model Dev.* **11**, 1377–1403 (2018).
7. Schaphoff, S. *et al.* LPJmL4 – a dynamic global vegetation model with managed land – Part 1: Model description. *Geosci. Model Dev.* **11**, 1343–1375 (2018).
8. Zabel, F., Putzenlechner, B. & Mauser, W. Global Agricultural Land Resources – A High Resolution Suitability Evaluation and Its Perspectives until 2100 under Climate Change Conditions. *PLOS ONE* **9**, e107522 (2014).
9. Hurtt, G. C. *et al.* Harmonization of global land use change and management for the period 850–2100 (LUH2) for CMIP6. *Geoscientific Model Development* **13**, 5425–5464 (2020).
10. Dietrich, J. P., Popp, A. & Lotze-Campen, H. Reducing the loss of information and gaining accuracy with clustering methods in a global land-use model. *Ecological Modelling* **263**, 233–243 (2013).
11. Dietrich, J. P. *et al.* luscale: PIK Landuse Group Data Scaling Tools. (2022).
12. MacDicken, K. G. Global Forest Resources Assessment 2015: What, why and how? *Forest Ecology and Management* **352**, 3–8 (2015).
13. Mishra, A. *et al.* Estimating global land system impacts of timber plantations using MAgPIE 4.3.5. *Geoscientific Model Development* **14**, 6467–6494 (2021).
14. Braakhekke, M. C. *et al.* Modeling forest plantations for carbon uptake with the LPJmL dynamic global vegetation model. *Earth System Dynamics* **10**, 617–630 (2019).
15. Lutz, F. *et al.* Simulating the effect of tillage practices with the global ecosystem model LPJmL (version 5.0-tillage). *Geoscientific Model Development* **12**, 2419–2440 (2019).
16. von Bloh, W. *et al.* Implementing the nitrogen cycle into the dynamic global vegetation, hydrology, and crop growth model LPJmL (version 5.0). *Geoscientific Model Development* **11**, 2789–2812 (2018).
17. FAOSTAT. Food & Agriculture Organization of the United Nations Statistics Division. *FAOSTAT Database* https://www.fao.org/faostat/en/ (2016).
18. Heinke, J. *et al.* A new climate dataset for systematic assessments of climate change impacts as a function of global warming. *Geoscientific Model Development* **6**, 1689–1703 (2013).

19. Pardey, P. G., Beintema, N. M., Dehmer, S. & Wood, S. *Agricultural Research - A Growing Global Divide?* https://ebrary.ifpri.org/digital/collection/p15738coll2/id/125246 (2006) doi:10.2499/089629529X.
20. Narayanan, B. & Walmsey, T. L. *Global Trade, Assistance, and Production: The GTAP 7 Data Base*. (2008).
21. IPCC. *2006 IPCC guidelines for National Greenhouse Gas Inventories. Agriculture, forestry and other land use (AFOLU)*. vol. Vol. 4 (2006).
22. Bodirsky, B. L. *et al.* N_2O emissions from the global agricultural nitrogen cycle – current state and future scenarios. *Biogeosciences* **9**, 4169–4197 (2012).
23. Chaplin-Kramer, R. *et al.* Global modeling of nature's contributions to people. *Science* **366**, 255–258 (2019).
24. Fisher, B., Turner, R. K. & Morling, P. Defining and classifying ecosystem services for decision making. *Ecological Economics* **68**, 643–653 (2009).
25. Suh, S. *et al.* Closing yield gap is crucial to avoid potential surge in global carbon emissions. *Global Environmental Change* **63**, 102100 (2020).
26. Johnson, J. *et al. Global futures: Modelling the global economic impacts of environmental change to support policy-making - technical report.* https://www.gtap.agecon.purdue.edu/resources/res_display.asp?RecordID=6186 (2020).
27. Johnson, J. A. *et al. The Economic Case for Nature: A Global Earth-Economy Model to Assess Development Policy Pathways*. https://openknowledge.worldbank.org/handle/10986/35882 (2021).
28. ESA. *Land Cover CCI Product User Guide Version 2*. Available at: maps.elie.ucl.ac.be/CCI/viewer/download/ESACCI-LC-Ph2-PUGv2_2.0.pdf (2017).
29. Verburg, P. H. & Overmars, K. P. Combining top-down and bottom-up dynamics in land use modeling: exploring the future of abandoned farmlands in Europe with the Dyna-CLUE model. *Landscape Ecol* **24**, 1167–1181 (2009).
30. Wolff, S., Schrammeijer, E. A., Schulp, C. J. E. & Verburg, P. H. Meeting global land restoration and protection targets: What would the world look like in 2050? *Global Environmental Change* **52**, 259–272 (2018).
31. Soares-Filho, B. S., Rodrigues, H. O. & Costa, W. L. *Modeling environmental dynamics with Dinamica EGO*. (2009).
32. Panagos, P. *et al.* Global rainfall erosivity assessment based on high-temporal resolution rainfall records. *Scientific Reports* **7**, 4175 (2017).
33. Borrelli, P. *et al.* An assessment of the global impact of 21st century land use change on soil erosion. *Nature Communications* **8**, 2013 (2017).
34. Borrelli, P. *et al.* Land use and climate change impacts on global soil erosion by water (2015-2070). *PNAS* (2020) doi:10.1073/pnas.2001403117.
35. Fuster, B. *et al.* Quality Assessment of PROBA-V LAI, fAPAR and fCOVER Collection 300 m Products of Copernicus Global Land Service. *Remote Sensing* **12**, 1017 (2020).
36. Copernicus Global Land Service. https://land.copernicus.eu/global/ (2021).
37. Hansen, M. C. *et al.* High-Resolution Global Maps of 21st-Century Forest Cover Change. *Science* **342**, 850–853 (2013).
38. Breiman, L. Random Forests. *Machine Learning* **45**, 5–32 (2001).

39. Wright, M. N. & Ziegler, A. **ranger**: A Fast Implementation of Random Forests for High Dimensional Data in *C++* and *R*. *J. Stat. Soft.* **77**, (2017).
40. Fick, S. E. & Hijmans, R. J. WorldClim 2: new 1-km spatial resolution climate surfaces for global land areas. *International Journal of Climatology* **37**, 4302–4315 (2017).
41. Hengl, T. *et al.* Global mapping of potential natural vegetation: an assessment of machine learning algorithms for estimating land potential. *PeerJ* **6**, e5457 (2018).
42. IPBES. *Global assessment report on biodiversity and ecosystem services of the Intergovernmental Science-Policy Platform on Biodiversity and Ecosystem Services*. 1148 (2019).
43. Mittermeier, R. *et al. Hotspots Revisited. Earth's Biologically Richest and Most Endangered Terrestrial Ecoregions*. vol. 392 (2004).
44. Potapov, P. *et al.* The last frontiers of wilderness: Tracking loss of intact forest landscapes from 2000 to 2013. *Science Advances* **3**, e1600821 (2017).
