# Supplementary material for: Projected landscape-scale repercussions of global action for climate and biodiversity protection
Source: Nat Commun. 2023 May 16;14:2515. doi: 10.1038/s41467-023-38043-1 (PMC10188494; doi:10.1038/s41467-023-38043-1)
Supplement: Supplementary file 3 — Reporting Summary [file 41467_2023_38043_MOESM3_ESM.pdf]

nature portfolio

Corresponding author(s): Patrick v. Jeetze

Last updated by author(s): Apr 6, 2023

Reporting Summary

Nature Portfolio wishes to improve the reproducibility of the work that we publish. This form provides structure for consistency and transparency in reporting. For further information on Nature Portfolio policies, see our Editorial Policies and the Editorial Policy Checklist.

Statistics

For all statistical analyses, confirm that the following items are present in the figure legend, table legend, main text, or Methods section.

n/a	Confirmed	
x		The exact sample size (*n*) for each experimental group/condition, given as a discrete number and unit of measurement
x		A statement on whether measurements were taken from distinct samples or whether the same sample was measured repeatedly
x		The statistical test(s) used AND whether they are one- or two-sided<br>*Only common tests should be described solely by name; describe more complex techniques in the Methods section.*
	x	A description of all covariates tested
	x	A description of any assumptions or corrections, such as tests of normality and adjustment for multiple comparisons
x		A full description of the statistical parameters including central tendency (e.g. means) or other basic estimates (e.g. regression coefficient) AND variation (e.g. standard deviation) or associated estimates of uncertainty (e.g. confidence intervals)
x		For null hypothesis testing, the test statistic (e.g. *F*, *t*, *r*) with confidence intervals, effect sizes, degrees of freedom and *P* value noted<br>*Give P values as exact values whenever suitable.*
x		For Bayesian analysis, information on the choice of priors and Markov chain Monte Carlo settings
x		For hierarchical and complex designs, identification of the appropriate level for tests and full reporting of outcomes
x		Estimates of effect sizes (e.g. Cohen's *d*, Pearson's *r*), indicating how they were calculated

Our web collection on statistics for biologists contains articles on many of the points above.

Software and code

Policy information about availability of computer code

Data collection

We linked the global land system modelling framework MAgPIE v4.3.5 (Model of Agricultural Production and its Impact on the Environment; written in GAMS and R) with the SEALS (Spatial Economic Allocation Landscape Simulator; written in Python) model. Globally gridded yield, carbon, and water input data was derived from the dynamic global vegetation, crop and hydrology model LPJmL (Lund-Potsdam-Jena managed Land; written in C++ and R). Our soil erosion estimates are based on the Global Soil Erosion Modelling (GloSEM) platform (implemented in R for this study). All models are referenced and described in more detail in the main manuscript. The model code of the MAgPIE model is openly available under the GNU Affero General Public License, version 3 (AGPLv3) and accessible via GitHub (https://github.com/magpiemodel/magpie). The release version (MAgPIE 4.3.5.), on which this study is based, has been archived via Zenodo (https://doi.org/10.5281/zenodo.5394196). MAgPIE 4.3.5. is accompanied by a technical model documentation (https://rse.pik-potsdam.de/doc/magpie/4.3.5/), which has been compiled with the GAMS code documentation toolkit goxygen.

Data analysis

Data analysis during post-processing was done in R by using the libraries 'luscale' v2.22.1, 'magpie4' v1.99.2 (both part of the MAgPIE modelling framework and available at https://github.com/pik-piam), 'terra' v1.7-3, 'exactextractr' v0.9.1, 'foreach' v1.5.2, 'doParallel' v1.0.17, 'ranger' v0.12.1 (references provided in the manuscript).

For manuscripts utilizing custom algorithms or software that are central to the research but not yet described in published literature, software must be made available to editors and reviewers. We strongly encourage code deposition in a community repository (e.g. GitHub). See the Nature Portfolio guidelines for submitting code & software for further information.

Data

Policy information about availability of data

All manuscripts must include a data availability statement. This statement should provide the following information, where applicable:

- Accession codes, unique identifiers, or web links for publicly available datasets
- A description of any restrictions on data availability
- For clinical datasets or third party data, please ensure that the statement adheres to our policy

All model outputs used in this paper have also been archived via Zenodo and can be accessed under https://doi.org/10.5281/zenodo.7804740. All input data used in this study, such as the input data for the GloSEM platform, is fully referenced in the paper.

Human research participants

Policy information about studies involving human research participants and Sex and Gender in Research.

Reporting on sex and gender	No human research participants were involved in this study.
Population characteristics	n/a
Recruitment	n/a
Ethics oversight	n/a

Note that full information on the approval of the study protocol must also be provided in the manuscript.

Field-specific reporting

Please select the one below that is the best fit for your research. If you are not sure, read the appropriate sections before making your selection.

☐ Life sciences ☐ Behavioural & social sciences ☒ Ecological, evolutionary & environmental sciences

For a reference copy of the document with all sections, see nature.com/documents/nr-reporting-summary-flat.pdf

Ecological, evolutionary & environmental sciences study design

All studies must disclose on these points even when the disclosure is negative.

Study description	This study applies a globally consistent, replicable and empirically calibrated modelling approach to examine how changes in the demand for land-based goods and ensuing global land-use dynamics could drive local changes in compositional and configurational landscape heterogeneity, pollination, and soil erosion across different land-use scenarios.
Research sample	We assess four different scenarios to compare the outcomes of different land-use trajectories on a range of indicators for changes in the supply of material and regulating NCP. The scenarios are all based on the 'middle of the road' shared-socioeconomic pathway (SSP2). We contrast a 'business-as-usual' (BAU) scenario (only currently implemented policies) with a scenario set that successively combines global measures for area-based conservation, carbon uptake on land ('climate policy') and a quantitative target for landscape restoration ('landscape policy').
Sampling strategy	No new data was collected for this study.
Data collection	All relevant input data used and processed in this modelling study is fully documented in the manuscript or in openly available online documentations, referenced in the text.
Timing and spatial scale	We mainly provide a global analysis, but also indicate regional- or country-level outcomes. We also map and present spatially-explicit outcomes at an intermediate and a fine-scale spatial resolution of 0.5 degree (55x55 km at the equator) and 10 arc seconds ('field-scale'; 300x300 m at the equator).
Data exclusions	No data was excluded.
Reproducibility	The model code (https://doi.org/10.5281/zenodo.5394196) and the model outputs (https://doi.org/10.5281/zenodo.5897593) are available online.
Randomization	Not applicable as no new data was collected.
Blinding	Not applicable as no new data was collected.

Did the study involve field work? ☐ Yes ☒ No

Reporting for specific materials, systems and methods

We require information from authors about some types of materials, experimental systems and methods used in many studies. Here, indicate whether each material, system or method listed is relevant to your study. If you are not sure if a list item applies to your research, read the appropriate section before selecting a response.

Materials & experimental systems

n/a	Involved in the study
☒	☐ Antibodies
☒	☐ Eukaryotic cell lines
☒	☐ Palaeontology and archaeology
☒	☐ Animals and other organisms
☒	☐ Clinical data
☒	☐ Dual use research of concern

Methods

n/a	Involved in the study
☒	☐ ChIP-seq
☒	☐ Flow cytometry
☒	☐ MRI-based neuroimaging
